# Supplementary material for: Multi-omics and pan-cancer analysis revealed common molecular signatures to disclose multitargeted anticancer agents through network pharmacology approach
Source: PLoS One. 2026 Jun 1;21(6):e0350614. doi: 10.1371/journal.pone.0350614 (PMC13225668; doi:10.1371/journal.pone.0350614)
Supplement: S1 Fig — (A) PPI network generated at a confidence score of 0.7, illustrating the refined interaction landscape after removal of low-confidence edges. (B) Top 10 hub genes ranked by degree centrality, highlighting the most connected and potentially significant nodes. (DOCX) [file pone.0350614.s001.docx]

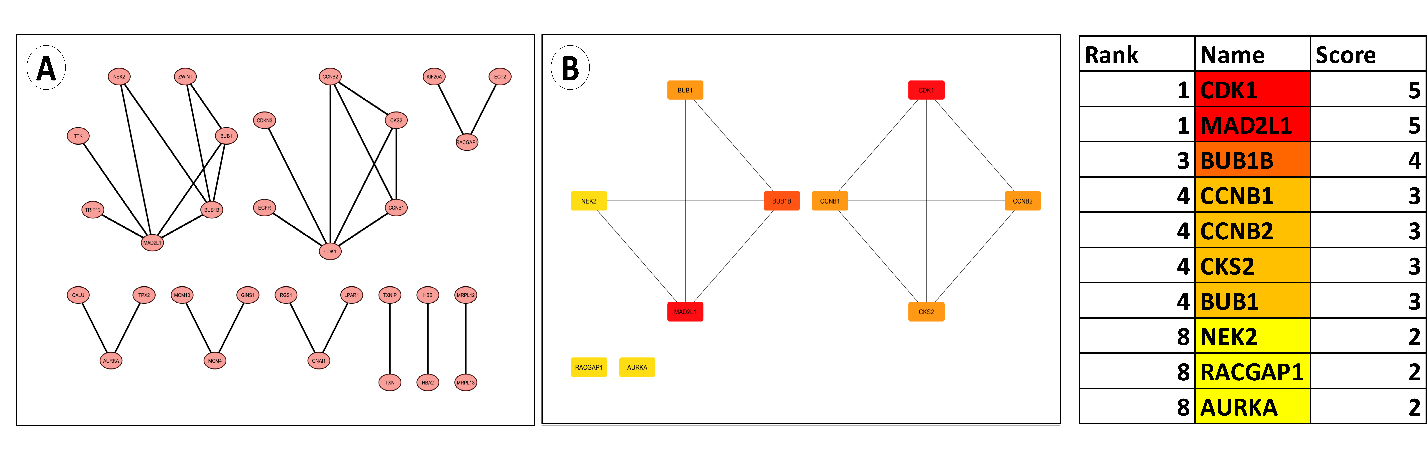


**S1 Fig:** Sensitivity analysis of PPI network using high-confidence threshold. (A) PPI network generated at a confidence score of 0.7, illustrating the refined interaction landscape after removal of low-confidence edges. (B) Top 10 hub genes ranked by degree centrality, highlighting the most connected and potentially significant nodes.
