# Supplementary material for: Multi-omics and pan-cancer analysis revealed common molecular signatures to disclose multitargeted anticancer agents through network pharmacology approach
Source: PLoS One. 2026 Jun 1;21(6):e0350614. doi: 10.1371/journal.pone.0350614 (PMC13225668; doi:10.1371/journal.pone.0350614)
Supplement: S2 Fig — Red boxes indicate tumor tissues and blue boxes indicate normal tissues. Statistically significant differences are marked as *P < 0.05, **P < 0.01, and ***P < 0.001. (DOCX) [file pone.0350614.s002.docx]

**
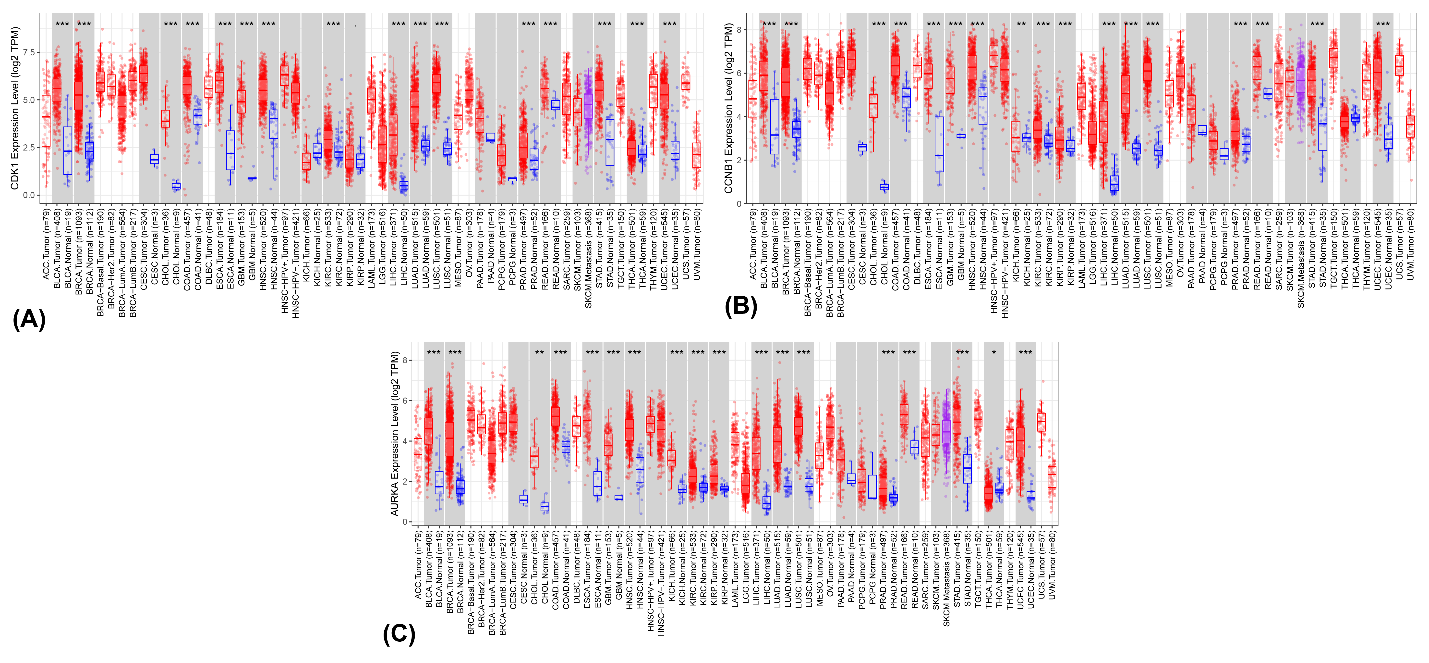
**

**S2 Fig.** Comparative expression profiling through Boxplot analysis of the cKHGs in various cancers and their pathological phases using TIMER2.0. Red boxes indicate tumor tissues and blue boxes indicate normal tissues. Statistically significant differences are marked as *P < 0.05, **P < 0.01, and ***P < 0.001.
