# Supplementary material for: Multi-omics and pan-cancer analysis revealed common molecular signatures to disclose multitargeted anticancer agents through network pharmacology approach
Source: PLoS One. 2026 Jun 1;21(6):e0350614. doi: 10.1371/journal.pone.0350614 (PMC13225668; doi:10.1371/journal.pone.0350614)
Supplement: S3 Fig — (DOCX) [file pone.0350614.s003.docx]

**
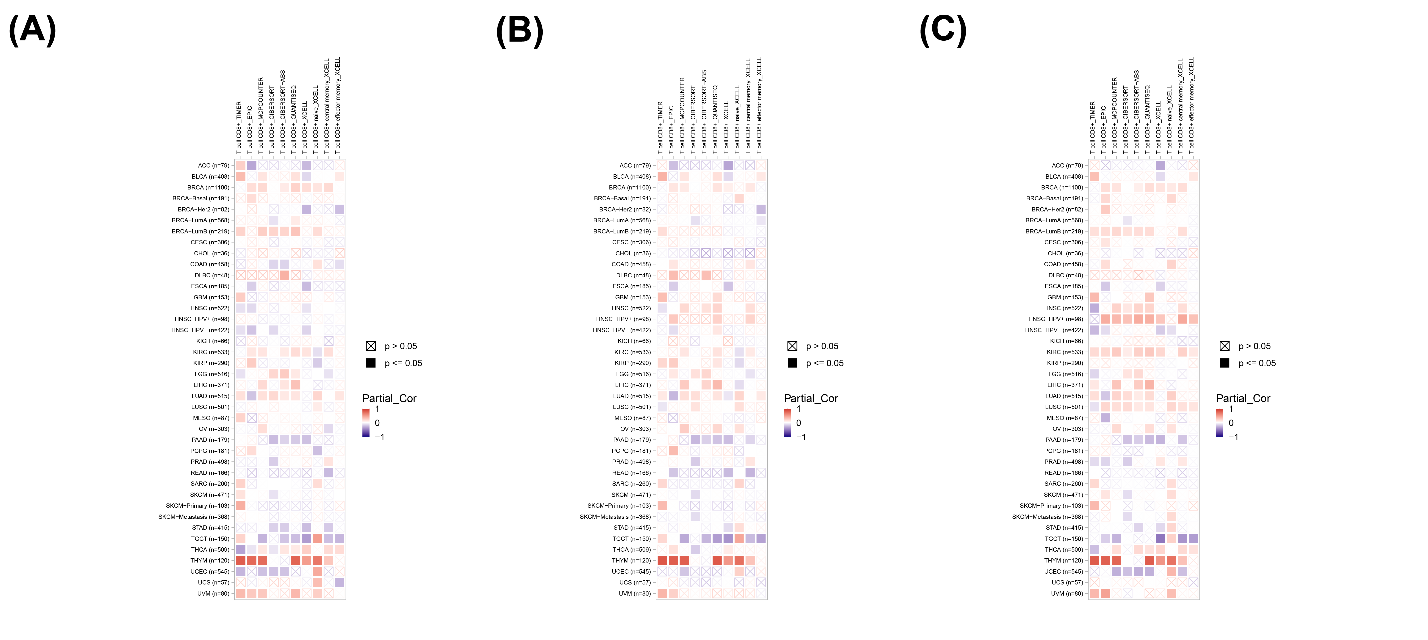
**

**S3 Fig.** Correlation of AURKA CCNB1 and CDK1 Expression with Immune Cell Infiltration Across CD8+ T Cells.
