# Supplementary material for: Multi-omics and pan-cancer analysis revealed common molecular signatures to disclose multitargeted anticancer agents through network pharmacology approach
Source: PLoS One. 2026 Jun 1;21(6):e0350614. doi: 10.1371/journal.pone.0350614 (PMC13225668; doi:10.1371/journal.pone.0350614)
Supplement: S4 Fig — (DOCX) [file pone.0350614.s004.docx]

**
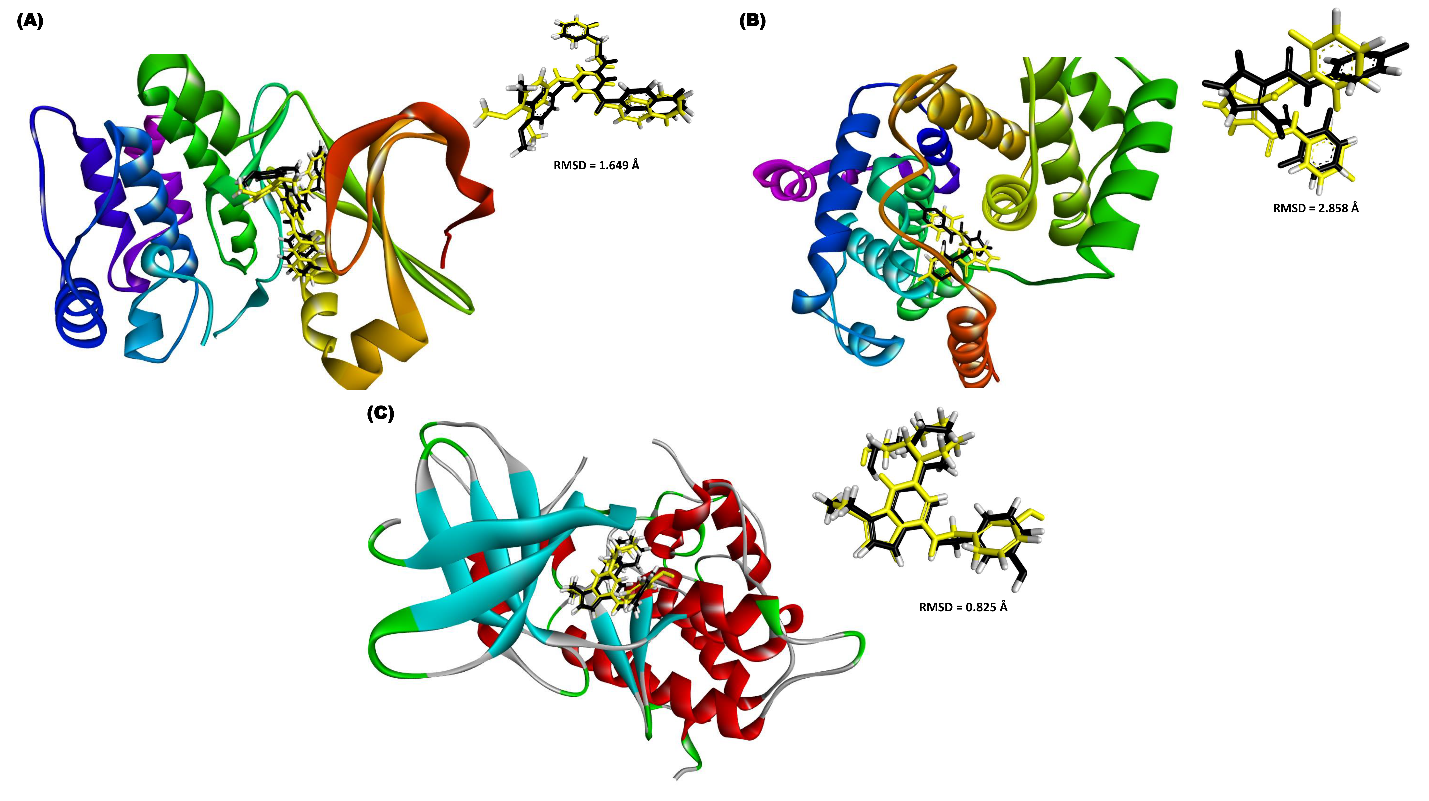
**

**S4 Fig**. Docking protocol validation by re-docking of native ligands (2JZ, LZ9, and 1QK) in (A) AURKA, (B) CCNB1, and (C) CDK1 showing RMSD values of 1.649, 2.858, and 0.825 Å, respectively.
