# Supplementary material for: Multi-omics and pan-cancer analysis revealed common molecular signatures to disclose multitargeted anticancer agents through network pharmacology approach
Source: PLoS One. 2026 Jun 1;21(6):e0350614. doi: 10.1371/journal.pone.0350614 (PMC13225668; doi:10.1371/journal.pone.0350614)
Supplement: S1 Table — (DOCX) [file pone.0350614.s005.docx]

**S1 Table:** Target Protein Informations and Molecular Docking Parameters

| Tragets | Properties | | Grid Box Coordinates | | | Grid Box Dimentions | | | Exhaustiveness |
| --- | --- | --- | --- | --- | --- | --- | --- | --- | --- |
|  | PDB IDs | Resolution | Center_X | Center_Y | Center_Z | X | Y | Z | 8 |
| AURKA | 3HA6 | 2.36 | -29.7514 | 35.1903 | 6.8552 | 54.104 | 52.551 | 55.363 |  |
| CCNB1 | 4Y72 | 2.3 | -1.0398 | -70.5657 | 209.9834 | 58.953 | 43.525 | 53.211 |  |
| CDK1 | 6GU6 | 2.33 | 18.4031 | 16.2649 | 9.9032 | 42.859 | 50.181 | 68.504 |  |
