# Supplementary material for: Multi-omics and pan-cancer analysis revealed common molecular signatures to disclose multitargeted anticancer agents through network pharmacology approach
Source: PLoS One. 2026 Jun 1;21(6):e0350614. doi: 10.1371/journal.pone.0350614 (PMC13225668; doi:10.1371/journal.pone.0350614)
Supplement: S2 Table — (DOCX) [file pone.0350614.s006.docx]

**S2 Table:** List of Unique Differentially Expressed Genes (DEGs) from Three Dataset

| **Unique upregulated Gene List** | | | **Unique downregulated Gene List** | | |
| --- | --- | --- | --- | --- | --- |
| **GSE45827** | **GSE21510** | **GSE26712** | **GSE45827** | **GSE21510** | **GSE26712** |
| UBE2N | MGST3 | CALR | SH3GL3 | DYRK1A | BNC1 |
| EZR | LRRC37A3 | C9orf16 | FRG1BP | FOXN4 | NELL2 |
| PRPF4 | IL1RAP | CD24 | SCARA5 | STAT1 | KDR |
| POLR3K | CT55 | PPDPF | KANK3 | LINC00202-2 | CLEC4M |
| RANBP9 | STAT1 | NOTCH3 | ANKRD20A4 | GH2 | ANXA8L1 |
| P4HB | DYNC2LI1 | RPL37A | SCD5 | TLE1 | PTGDR |
| ACLY | DGCR9 | GSTP1 | GPIHBP1 | SYT9 | CLDN15 |
| GMFB | TRIM65 | CLDN3 | LOC441666 | RUVBL2 | C21orf62 |
| SRPK2 | G3BP1 | SOX17 | DNAH3 | PGD | NKX3-1 |
| PSENEN | MIR4435-2HG | PFN1 | TRIM4 | MYLIP | LGALS8 |
| SPATS2 | CASP2 | HIST2H2AA4 | PIR-FIGF | LOC100287704 | PNRC2 |
| HACD3 | MGC70870 | SNORA68 | RECK | TRABD2A | DFNA5 |
| MIR3620 | FRA10AC1 | PKM | OPHN1 | NYAP1 | S100PBP |
| M6PR | SMC6 | CNOT3 | COLCA1 | CLDN23 | CREBL2 |
| UBXN4 | GPATCH2L | FKBP8 | CA4 | KIF1C | MTUS1 |
| LARP4B | PDCD5 | LOC101929219 | GPD1 | HRASLS2 | HNRNPD |
| KDELR1 | CTC-338M12.4 | PRMT1 | FGF7P3 | UBE2B | RBL2 |
| RAB3GAP2 | FOXP1 | GRINA | CD300LG | CTLA4 | FEZ2 |
| GSPT1 | PTPN4 | MECOM | SCN4B | AK7 | WNT2B |
| PIGC | HEATR1 | MRPL2 | TNXB | NACAP1 | SLC46A3 |
| AKT1S1 | MBD4 | ARF5 | ALDH1L1 | CADM3 | ZNF350 |
| DDA1 | ZNF302 | GANAB | TUSC5 | PFKP | PDE8B |
| WAPL | ZNF19 | MAZ | ARHGEF15 | SYNDIG1L | GCOM1 |
| ARPC4-TTLL3 | CCAR1 | MIR1282 | FXYD1 | LINC01523 | ATM |
| MIR4745 | DNMT3L | BCL2L2-PABPN1 | ANKRD20A12P | ZER1 | PCDH9 |
| RIT1 | METTL3 | SLC52A2 | TESC | LPO | LOC101928635 |
| METTL2A | WDR12 | IGFBP2 | LEP | RBP3 | HSD17B2 |
| MRPL42 | COX17 | ATP5H | CWF19L2 | TBL3 | DSC3 |
| C16orf72 | CLEC4A | S100A13 | RPS11 | TRPM3 | TPD52L1 |
| CALU | NPHP1 | PEA15 | ATP1A2 | ZNF775 | SPOCK1 |
| ATP6V1A | CCP110 | B3GAT3 | LINC00408 | MLYCD | ADH1C |
| ALYREF | AZI2 | KLHDC3 | CLDN5 | CYTH1 | STK26 |
| ENSA | GEMIN2 | PNN | COPG2IT1 | OVOL2 | TTBK2 |
| RAB22A | LOC102724275 | ARPC4 | TCTN2 | VIPR2 | GLS |
| SEC24A | RHOT1 | HMGA1 | CFD | ZNF264 | CALB2 |
| MAPRE1 | SSBP1 | SCAMP4 | LOC100507311 | RAB40AL | LOC101926921 |
| NOLC1 | NCKAP1 | RNF19B | BMPR1A | KRBA2 | RPL23AP32 |
| XPNPEP1 | C9orf3 | PPP5C | MOCS1 | SLC4A4 | SCTR |
| SDHC | BRCA2 | H2AFX | ITIH5 | ELSPBP1 | PLCE1 |
| TSN | FASTKD2 | ELF3 | PEAR1 | FCER2 | REEP1 |
| PPP1R11 | PYROXD1 | CLDN4 | LOC101930100 | GPR161 | USP12 |
| C3orf38 | ZMYM2 | SOX9 | PLAC9 | ARHGAP27 | DPY19L1 |
| SMC4 | MDM4 | DDA1 | NDUFC2-KCTD14 | ENTPD4 | ZNF45 |
| LRRC41 | LINC00869 | CRABP2 | NPR1 | FAM120AOS | NRXN3 |
| CFL1 | GTF2IP12 | MIR7113 | CDC5L | MIR3620 | ZNF175 |
| SAE1 | TRMT10A | NUCB1 | CIDEC | LINC00652 | HEG1 |
| SRSF1 | YTHDC1 | PTK7 | ACACB | ZNF81 | PHLPP2 |
| YIF1B | MYNN | ABCF3 | HLF | CUX1 | ZNF23 |
| SETD5 | CEP44 | FOLR1 | FOXN3 | SYMPK | GPM6A |
| UBE2M | INIP | COPG1 | RBP4 | CFLAR | KIAA0226L |
| RAB5C | SVIP | UBE2C | C21orf2 | TWF1 | ETFDH |
| AREL1 | U2SURP | KDELR1 | ACVR1C | VAMP2 | ATP10D |
| ARFGEF1 | ANKRD10 | WFDC2 | TMEM132C | ATP6V0A1 | PRG4 |
| YWHAH | ZMYM5 | RPS7 | FUNDC2 | ZBTB7B | MARK3 |
| CSNK2A1 | LIN9 | ZNF593 | PLIN4 | DUSP1 | SECISBP2 |
| UBE2H | CEP57L1 | LRP5 | INMT | KLHL14 | ANKRD12 |
| TPD52 | TNPO3 | DBN1 | ABCA9 | TGFB1 | SGCG |
| FYTTD1 | SOX6 | YWHAE | GSTM5 | RS1 | PTGER3 |
| KCTD5 | BRWD1 | BTBD2 | BTNL9 | SCIN | PIP5K1B |
| NEMF | PNO1 | COPE | RELN | MBTPS1 | CDC14B |
| NFYB | MRPL39 | JUP | LIPE | LOC284570 | MCTP2 |
| SEC61A1 | ZNF26 | HSPG2 | TP53AIP1 | ZNF653 | PTPRZ1 |
| PRPF4B | SDCCAG8 | BCL7C | SPDYE2B | FAM107A | SARAF |
| TMEM9B | PDS5B | MAP7D1 | GPR146 | PRKCSH | DPYS |
| AGTRAP | TFG | ANP32A | ROBO3 | LMNTD1 | RNF128 |
| PPIF | ICK | TPX2 | PDE2A | YIF1B | ING3 |
| B3GNT2 | MKLN1 | CORO1B | ADAM33 | LRRFIP2 | MAF |
| GDE1 | ASXL2 | FASN | LPL | PRR13 | MNDA |
| RANGAP1 | LINC00342 | ABHD11 | ADH1B | LOC644172 | ZNF330 |
| LSM4 | OFD1 | JUND | POU6F1 | CCDC106 | MRPS14 |
| PLAUR | EXOSC3 | PAX8 | SLIT3 | CADM3 | SLC4A4 |
| USP38 | NUB1 | RPS10-NUDT3 | SPTBN1 | EXOC3L2 | ST3GAL5 |
| CSNK1A1 | WDHD1 | SCGB2A1 | ITGA7 | SIRT2 | RYR2 |
| ARF4 | ODF2L | HSF1 | RPL37A | KCNIP4 | CXorf57 |
| WAC | MIR6875 | CHD8 | ADH1C | EDN3 | VGLL3 |
| CLPTM1 | CD44 | ACTN1 | OCIAD1 | NR1I2 | PTP4A2 |
| PRPF31 | MRPL50 | SNRPE | ROBO4 | TNKS1BP1 | FGF13 |
| HSPH1 | CCDC150 | INTS3 | TMEFF2 | KCTD2 | AQP9 |
| ELAVL1 | ATP5O | RBBP4 | COL6A6 | CYP2A7 | CFI |
| FUS | CPSF6 | LRFN4 | TIMM8A | GIPC1 | TSPAN5 |
| RAB1A | LOC100506639 | C1orf106 | CPSF2 | USF2 | SLC16A1 |
| KIF2A | TXNDC9 | TUBA1C | TMEM241 | ASXL3 | PSD3 |
| TSEN15 | ARGLU1 | PRKCSH | HOXD9 | PHF23 | ZNF112 |
| TRIM59 | FOPNL | CLPTM1 | ARID5A | PQLC2 | ARHGAP44 |
| CD46 | TAF9B | HGS | ADAMTS9-AS2 | RBFOX1 | LOC101930363 |
| RAD1 | ELOVL5 | NT5DC2 | SNX33 | NFKB2 | SLC31A2 |
| TRIM27 | ZNF780B | TRIP13 | LRRN4CL | LOC105371374 | TMEM255A |
| PPP2R5C | ESD | H2BFS | ADIPOQ | HLA-G | UBL3 |
| XRN2 | GPATCH2 | BANF1 | PRINS | MFSD2B | DAPK1 |
| RPRD1A | CXCR2 | VWA1 | TNFSF12-TNFSF13 | DLG3-AS1 | HNRNPA1P33 |
| DNAJC10 | SNORA72 | UBAP2L | BCORL1 | MBOAT7 | DCUN1D4 |
| MRPL44 | RPL5 | MRPL4 | HSPB2 | UHRF1BP1L | NPY1R |
| NIPBL | SP100 | SYNGR2 | FAXDC2 | FAM129B | TXNIP |
| KIAA0101 | SH3BP2 | S100A11 | MIR6716 | CIC | EP300 |
| TMED2 | ZNF572 | CARHSP1 | CFAP157 | IGHM | CYFIP1 |
| TMEM165 | C5 | COL4A1 | EZH1 | IGH | NBEA |
| CKAP5 | ALDH1B1 | ADRM1 | GSN | ZDHHC5 | PLSCR4 |
| MAPK1 | TMEM39A | RPN1 | WDR86 | SPG21 | SPAG9 |
| SRSF10 | IVNS1ABP | SORT1 | G3BP1 | CCM2 | FAM65B |
| DNAJB1 | CEP295 | DCAF15 | VPS51 | NOP9 | AOX1 |
| MSH6 | STRBP | CDC42BPB | SEMA3G | MYO1C | EPS15 |
| PSEN1 | HNRNPDL | MAP2K2 | SAMD5 | SYDE1 | PLCL2 |
| DVL3 | KIAA1024 | SLC25A11 | SLC4A4 | GAPDH | OGT |
| FAM120A | KHK | PRSS8 | RHOJ | CSF1 | PAXBP1 |
| YIPF5 | PRKRA | SMARCB1 | ADH4 | MUC4 | RGL1 |
| SEC23IP | MIR34A | ACTN4 | TIMP4 | DPT | RAB1A |
| DAZAP2 | ZNF146 | OAZ2 | GPAM | PPP6R1 | PHKB |
| CPSF2 | FNTA | SLC2A4RG | MUC3A | MAVS | SEMA5A |
| PNP | LOC101060604 | CLDN7 | RPS10 | ELMSAN1 | XPA |
| USP21 | MSL3 | HNRNPL | USP7 | STK16 | TMEM9B |
| ABCF2 | ZNF236 | HJURP | PLIN1 | DCLK2 | UHRF1BP1L |
| KIAA1033 | EED | PDLIM7 | SIK2 | ZNF358 | PEX12 |
| ASCC1 | MTHFD2L | GSDMD | MTMR9LP | RING1 | AKAP11 |
| ARL8B | RBBP6 | ARF6 | RAPGEF3 | ERN2 | ZNF37BP |
| ARIH1 | INTS6-AS1 | RAB5B | RPL27A | OCIAD1 | NAP1L2 |
| RFWD2 | FUBP1 | RHOB | PIGR | RANBP3 | WSB1 |
| SNORD14D | RBM12B | KIF4A | CYP1A2 | METTL7A | PIGB |
| SMARCC1 | ICE2 | SET | NRG2 | DRD5 | LOC100506718 |
| ALDH18A1 | CDC27 | OGFOD2 | MYH11 | CAPZB | ITGAM |
| NUP98 | PCNX1 | FOXM1 | LOC55338 | CCL23 | PRDM10 |
| PDAP1 | CD47 | ZER1 | NRIP2 | UBE2J2 | CYP39A1 |
| PPP5C | SRGAP2C | SDC3 | CD36 | GTPBP1 | PCF11 |
| TWF1 | PRKCQ-AS1 | KLK8 | FOXO4 | STRA13 | SNCAIP |
| CACUL1 | OTUD7B | CCT5 | FABP4 | GNA11 | SEC11A |
| STK38 | SMARCA5 | SNRNP40 | FGF7 | LOC100286925 | HAND2-AS1 |
| TMEM245 | TCTE3 | BIRC5 | MCM3AP | SHROOM1 | TNPO1 |
| NDRG3 | PHF3 | ARFIP2 | PNPLA2 | CAPNS1 | ROCK1 |
| MIR636 | CCNL1 | YIPF2 | SNORD23 | SH3BP2 | NFYB |
| SFPQ | YARS2 | CDK16 | NAALADL1 | SKAP2 | PREPL |
| TPM4 | ZNF273 | UBE2Z | LIMS2 | UNC5B | RHOT1 |
| ARFRP1 | CASC15 | OGFR | TNAP | FXYD1 | CIRBP |
| ARPC5L | NBPF20 | CALU | PPM1F | MPZL1 | ALDH3A2 |
| RBM12 | CSTF3 | KLK6 | LTBP4 | LOC102724312 | HBG2 |
| C1orf43 | OGT | SLC39A4 | PPP1R14A | NFE2L1 | SEL1L3 |
| GADD45B | USP37 | ERF | PHF1 | LOC101927137 | MTO1 |
| COPS8 | SFXN1 | GCDH | SLC15A1 | EMC10 | TRIM2 |
| GNAI3 | SSR3 | IDH2 | RPL38 | GRN | CCSER2 |
| ATG5 | MSC-AS1 | NEK2 | RNF180 | PTGER4 | NOL7 |
| TRAPPC11 | FAM76B | UBE2S | MLXIPL | BSDC1 | EML1 |
| ZNF281 | C5orf34 | LOC101929368 | ANGPTL1 | PAK4 | GCNT1 |
| MAPKAP1 | SNORD68 | TTK | ADGRD2 | TAF6 | GAS1 |
| ZNF207 | EIF2AK2 | EPHB2 | EXOSC7 | ARHGDIA | ATF7IP2 |
| MAN2A1 | FAM122C | AAMP | PAMR1 | RPS6KA4 | DSE |
| AKIRIN1 | UGGT2 | SETSIP | ACSM5 | NUCB1 | VPS54 |
| TRAM1 | SASS6 | DFFA | BLOC1S1-RDH5 | EGR4 | IL18 |
| PPP1R2 | ANKRD28 | PPP4C | RPS9 | ARHGAP1 | SETX |
| TOR3A | ZNF10 | PTTG1 | GYG2 | PRELP | NANOG |
| GDI2 | MED31 | ATP6V0C | ECSCR | FABP2 | ALAD |
| FOLR1 | TIA1 | TMEM265 | LOC101930168 | IL17C | SAP18 |
| COG5 | FANCB | AGPAT2 | ADAMTS5 | CYB5R3 | CADPS2 |
| CNPY3 | TMEM267 | ADGRG1 | CDKN1C | TM6SF2 | FYN |
| AP3B1 | GALNT10 | GALNT6 | ZFP14 | CA4 | OSBPL1A |
| BPNT1 | ZNF283 | PTPRU | ZBTB4 | LYPLA2 | PTPRE |
| ATAD2 | GCFC2 | SRRM1 | AOC3 | SLC22A5 | CCDC93 |
| RAB3GAP1 | CBWD7 | HYOU1 | PNO1 | NAAA | GFPT2 |
| DCTN5 | ATP5S | ATP2A2 | DCAKD | GRAMD4 | UBE2I |
| GNB1 | PRPF40A | EIF5A | FUT8-AS1 | RNF167 | PLCG2 |
| TMEM41B | GPR75-ASB3 | LSM4 | CLDN19 | RAB5B | MARCO |
| KPNA1 | FARSB | H3F3AP4 | ADCY4 | IBA57 | HNRNPA1P10 |
| TM9SF3 | MALSU1 | CAPN1 | LOC101927345 | B3GNT6 | GALC |
| PAK2 | TNS4 | ASF1B | C2orf40 | BLOC1S1-RDH5 | FAM134A |
| TMEM206 | ZC3H6 | HN1 | TCF7L2 | SLC30A4 | BCAR3 |
| GFM1 | PGAP1 | CP | LINC01089 | SFRP1 | SLITRK5 |
| ACTR2 | LOC102724985 | UPK3BL | SYDE1 | SDF4 | BTAF1 |
| FGFR1OP | C11orf1 | MCL1 | ACADS | RAB11FIP1 | PDGFC |
| CYB5R3 | SLC25A36 | TUBA1B | PGM5 | PTK2B | RABGAP1L |
| CCT2 | SNORA16A | EPHX1 | MCTP2 | OGDH | UFSP2 |
| DHX15 | CCDC138 | TPI1 | PCNX1 | ARHGAP26 | LANCL1 |
| TAGLN2 | MATN1-AS1 | SLC4A5 | EBF1 | EWSR1 | NR0B1 |
| YWHAE | ITGB8 | TAGLN2 | LYVE1 | UBTF | WRB |
| MTPAP | ATP5E | ZYX | CCDC163 | C11orf86 | OLFML1 |
| ECT2 | MRPL19 | ARID1A | DPT | TMEM175 | SH3GL2 |
| RIOK3 | CEP350 | UCP2 | PTPRS | SPECC1 | ME1 |
| ABHD12 | NDUFB3 | BOLA2B | MGST3 | SPTAN1 | WWP2 |
| MED23 | ASIC1 | PRKDC | GNAL | CLSTN1 | TRIM68 |
| INTS7 | C2orf27A | STIP1 | EDNRB | FAM151A | CSGALNACT2 |
| WHSC1 | HELQ | CIZ1 | SMAD4 | CPM | HERC1 |
| SGPL1 | ZNF708 | G6PD | LOC286437 | MDGA1 | LOC100294033 |
| MAPKAPK5 | FAM98A | LOC100506248 | ZNF236 | LRRK1 | RABGAP1 |
| MPDU1 | MIRLET7D | GPBP1L1 | ANKRD65 | SLC25A34 | ATP8A1 |
| LOC101929368 | GRAMD1A | OGDH | PPP2R1B | APOBR | AKAP7 |
| UBE2G1 | PPP4R3B | BCAM | FAHD2CP | SMURF1 | SEC63 |
| UPK3BL | STK4 | BLOC1S1 | ANKRD53 | LOC101928405 | TACC1 |
| FRYL | NSMCE4A | EIF4G1 | SAA2 | CLDN5 | NEK9 |
| TTC3P1 | USP25 | MCM3 | LOC91548 | MED16 | SNX1 |
| ZNF12 | LRRC69 | ACTB | TPM2 | PLIN3 | ARMC8 |
| CDC42SE1 | FAM200B | SMG7 | SORBS1 | TBC1D17 | MPDZ |
| NIPSNAP1 | TTC28-AS1 | PRAF2 | PNPLA7 | NPLOC4 | TPP1 |
| FBXO21 | INPP4A | COX7B | WDTC1 | ACTR1A | SRSF6 |
| MBD4 | BCLAF1 | EIF4EBP1 | LOC101930064 | ACO2 | IL16 |
| GFPT1 | CHML | BCL11A | KLF15 | NFKBIB | CAPN2 |
| TLK2 | CDH6 | TUBB | SUGP1 | FOSL2 | TMEM50A |
| SLC25A39 | TRMT13 | PUF60 | SMC1A | TRAFD1 | CASP1 |
| HSP90AB1 | ARID4B | NUTF2P4 | C4orf46 | ARPC2 | FGF1 |
| SNHG4 | MIOS | PML | LOC101927104 | TMEM234 | UBXN2B |
| OAZ2 | RICTOR | CENPT | SYF2 | BTNL3 | ASAH1 |
| VDAC1 | SCAF4 | ARHGDIA | WASF2 | IDS | KIAA0368 |
| RAB10 | FXR1 | MCM10 | NOTCH4 | DAP | PHACTR2 |
| C21orf33 | PTPRK | PKP4 | STX12 | YWHAE | TTC28 |
| IGF2R | TANK | FAM171A1 | GGCX | MVP | MAP4K5 |
| H2AFY | CRIPT | UQCRQ | PCK1 | PDZD3 | ALDH9A1 |
| RABIF | LOC101927668 | FLOT1 | MMRN2 | GUCA2B | NF1P9 |
| PTP4A1 | ADAT2 | C20orf27 | LOC105379426 | BMP1 | NT5C1B-RDH14 |
| PIGX | KIAA1107 | ERBB2 | HADHA | HLA-J | CAST |
| TBCC | C11orf80 | TRIM28 | LOC101928635 | GALE | RSRP1 |
| SAR1B | AGBL3 | NES | GOLGA8DP | ZC3H3 | EFEMP1 |
| SRP72 | DNAJC7 | NUSAP1 | ATOH8 | GOLGA2 | VAMP4 |
| RAB5A | LOC105369662 | SLC44A4 | MME | AMN | PITPNC1 |
| LRIF1 | RGS5 | ZFP36L1 | LOC101243545 | PNPLA6 | RPL22 |
| ZNF322 | DAAM1 | PAM16 | SCN3A | CACFD1 | PLPP1 |
| HNRNPC | FBN2 | MFSD10 | ARHGEF40 | MIR6785 | FRY |
| HNRNPF | RPE | DGCR6L | ZNF747 | NUMBL | IP6K2 |
| BRD2 | PNN | MIR6734 | ZNF358 | NIT1 | ARMCX1 |
| STRBP | RNF139-AS1 | SMARCC1 | RPL23AP32 | IP6K1 | ARAP2 |
| DDX3X | LOC284513 | POLR2I | DSPP | GPS2 | MEIS2 |
| SS18 | FANCM | MUC1 | RILP | PI4KB | ADO |
| CLCC1 | SPEF2 | LRRN2 | JADE1 | NELFB | PPWD1 |
| ZNF627 | GFM2 | MIR7112 | LOC102724561 | RUNDC1 | ZKSCAN7 |
| PGK1 | ZNF148 | CDC20 | TAGLN | WDR1 | HOXC6 |
| CDYL | MIPEPP3 | PRKAR2A | WDR89 | FBXW4 | ERCC5 |
| PIK3C2A | WDR53 | TCEB2 | FRG1JP | YBX2 | MSRB2 |
| C14orf1 | CCDC191 | RAD23A | PGM5-AS1 | C6orf47 | AGGF1 |
| NUSAP1 | GART | RAB8A | SCRN2 | CLIP2 | GIPC2 |
| PSMB4 | RBM25 | PLVAP | FAM189A2 | PLEKHB2 | PNISR |
| BCL2L11 | TMEM206 | LAMTOR2 | NAT8L | ACTN4 | RAP2C |
| PPP4C | SUPT20H | SUB1 | SOBP | RCE1 | FYCO1 |
| COA1 | PHIP | MIR4745 | SORBS3 | CDC42EP4 | BRD1 |
| TNFRSF12A | GNAS | LAMC1 | SUGT1 | MAPK7 | WT1 |
| SPTLC2 | KIDINS220 | SIVA1 | NISCH | SRRM1 | TMEM5 |
| RC3H2 | BNIP2 | AGPAT1 | KLB | CIDEC | DAZAP2 |
| USP34 | PANK2 | PRRC2A | CHRDL1 | ARPIN | UBR2 |
| SEC23B | NR2C1 | XAB2 | IL11RA | KRTAP3-1 | WDR37 |
| KDELR3 | AGO3 | CHIC2 | ABTB1 | DIP2A | CHGB |
| PIP4K2A | PRPF4B | OVOL2 | RSBN1 | MIR4746 | FAM13B |
| BAZ1A | TYMP | HIST1H2BE | SIDT2 | KAT5 | GLUL |
| STIP1 | DHX30 | CDC42EP4 | AMT | UNC5C | NBPF20 |
| APPL1 | HNRNPA2B1 | PPP1R14B | RAP1A | BTBD2 | PRKAR1A |
| MAPKAPK2 | OCIAD1 | ERH | SOD3 | FBLIM1 | HSD17B6 |
| SRSF3 | NOL8 | DTL | B4GAT1 | RAB1B | AP1S2 |
| TAF12 | TRAM1 | CDCA8 | UBXN1 | PINK1 | ZDHHC17 |
| POGK | NACA | PEBP1 | ARHGEF28 | CA1 | NCOA4 |
| SRP9 | PSMB7 | RFXANK | PRKD2 | SLC8B1 | SDCBP |
| EZH2 | INTS6 | LOC101927180 | FCRL2 | GTF2F1 | PDGFD |
| HMG20B | SNX5 | STAB1 | CCDC8 | ACTB | CSTF2T |
| OSTC | DOCK9 | SRM | BCL6 | SLC35A4 | RNF103-CHMP3 |
| ARFGEF2 | RBM15 | UBE2M | SPTAN1 | CNNM2 | COL4A3BP |
| TOR1A | AHSA2 | S100A6 | MAP4 | GBA2 | TBC1D12 |
| ZWILCH | DENND4A | HEY2 | MAP2K5 | POLD4 | APPL1 |
| FAM76A | HEATR3 | MCM2 | NPFF | RHOB | ARHGEF10 |
| TIGAR | SNX14 | ESRP1 | CIRBP | ESPN | C7orf55-LUC7L2 |
| TM9SF4 | CREBZF | TMED2 | RPL21P28 | HPS1 | CHRDL1 |
| POLE3 | NSL1 | ISOC2 | LOC100289283 | SCAMP2 | HBD |
| PATL1 | LINC01355 | YWHAH | LMOD1 | C1orf210 | CELF2 |
| NCBP1 | TCERG1 | TSTA3 | ITSN1 | EHBP1L1 | GSAP |
| SNX4 | STAG3L4 | SF3A2 | ZNF219 | RAX | TSPAN13 |
| ARL1 | KLHL31 | CCNE1 | MKL2 | TRIP10 | DHX15 |
| SCYL2 | LOC102723694 | DRAP1 | FKSG49 | PPP1R11 | COBLL1 |
| VTA1 | HSPB11 | AGRN | DHRS4-AS1 | DYNC1LI2 | CACNB2 |
| GEMIN7 | ZNF215 | PKP3 | TNS1 | GUCD1 | HSPA12A |
| ZWINT | UBE2D1 | CAPZB | HAB1 | TBL1X | KBTBD11 |
| CUL2 | RASA2 | ATRIP | SERP2 | RNF125 | MST1 |
| TIMM17A | ATG4C | RUVBL2 | CEBPA-AS1 | CRK | PROCR |
| CNOT3 | GAS2 | PDE9A | PREX2 | MOB3A | TMED10 |
| PRCC | CCNT2 | S100A2 | AK5 | PDK2 | PODXL |
| RSPRY1 | NKTR | AURKAIP1 | RNPEPL1 | RALGDS | RAD17 |
| YWHAZ | ARL6 | FXYD3 | QKI | HNF4A | RUNX1T1 |
| JAK1 | ANAPC10 | CKS2 | CCDC69 | ZDHHC24 | IFFO1 |
| SPAG1 | MSI2 | TOMM34 | IGFBP6 | TNXB | METTL7A |
| DDX59 | ARMC8 | BIK | TGFBR3 | ANAPC16 | FZD7 |
| SLC17A5 | SFT2D3 | LTBR | SNX21 | SZRD1 | TRAPPC11 |
| OSBPL11 | NFXL1 | COL1A1 | HPS1 | DNAJC11 | RASA1 |
| AIDA | LOC102724093 | IL32 | LMNA | B3GALT5-AS1 | UFL1 |
| DPP3 | LCORL | KIF2C | TTC38 | CTBP2 | ATP6V1A |
| CYB5R4 | RPS19 | MMP14 | MINK1 | MOB2 | SLK |
| ATP2A2 | UQCRB | SLC50A1 | ACAP1 | RNF10 | PCSK5 |
| NSUN4 | TMSB15B | ST14 | LINC00266-1 | SH3D21 | CUX1 |
| TLE3 | NIFK | EXOSC4 | FAHD2A | ACP2 | DZIP3 |
| PREPL | KIF23 | PIP5K1A | RPF1 | EGFL7 | SFRP1 |
| ODF2 | DNAH14 | RPL38 | ACCS | FAM160A2 | TRPC1 |
| ZNF148 | ORC4 | GINS1 | G0S2 | CAPN1 | N4BP2L2 |
| WDR1 | LOC105379045 | RAE1 | NAAA | ARPC1A | TAOK3 |
| SLC35E1 | ELK4 | MIIP | SDPR | OS9 | SNCA |
| MGAT2 | RIF1 | ARPC4-TTLL3 | KRAS | TFG | MORF4L1 |
| RBM8A | FAM133DP | CBS | NDRG2 | ARF3 | PEX11A |
| PLEKHB2 | PCNX4 | MARCKSL1 | GTPBP3 | PCSK7 | SLC39A8 |
| MACF1 | FANCL | HIST1H2BD | PI16 | SRPRA | EZR |
| NLN | ERI2 | LAD1 | N4BP2L1 | MIR6791 | RBM5 |
| ZFR | ZBTB44 | GLB1L2 | COX7A1 | TSSC4 | LOC101928830 |
| HPCAL1 | ATR | JUNB | PLCL2 | TADA2B | DDX17 |
| TIMM23B | PIN4 | INHBC | TSHZ2 | SEMA4B | RAB11FIP2 |
| MAP4K5 | URI1 | GDI1 | MYCT1 | TCN2 | EFHC1 |
| UHMK1 | MDN1 | MRPL12 | GLG1 | ACOT11 | FOXJ3 |
| IL13RA1 | CENPQ | SETSIP | FAM162B | ATP13A4 | RTN1 |
| TRNT1 | MINA | LOC101927673 | ADGRA2 | TNIP1 | SH3GLB1 |
| PLEKHF2 | LNPK | NDUFV2 | NSG1 | RHOC | PDLIM5 |
| GORASP2 | SERPINB9 | RALGDS | ANAPC4 | NXPE1 | TPBG |
| RHOA | LOC101927446 | NR2F6 | PEX3 | P2RX5-TAX1BP3 | FRMD4B |
| WDR48 | LOC101929147 | MCM4 | MAGI1 | RNPEP | NSA2 |
| CPEB4 | SETDB1 | UBE2N | HSPB6 | ATG4B | TFPI2 |
| TMEM30A | MIR21 | MMP11 | CXorf36 | CEP170B | MICA |
| ASNA1 | IKBKAP | NDUFB7 | TRMT61A | IGSF9 | RNF38 |
| RAB7A | TRUB1 | HNRNPUL1 | NFIX | SP110 | PCCA |
| KMT5A | SNRPE | MT1X | KLHL13 | DCAF5 | SLTM |
| DCAF10 | RPAIN | SDC1 | TMEM198B | SNAP29 | WDR44 |
| NAA50 | MNAT1 | NOP10 | CRTAP | ADTRP | ANKRA2 |
| RBM15 | DPH6 | TRIM27 | FHL1 | ARPC4 | IK |
| ATF1 | TAS2R14 | PAK2 | RBP7 | KLF13 | CARMIL1 |
| TAB2 | BTF3L4 | CIC | CAPN3 | NADK | TFPI |
| RELA | YEATS4 | ATOX1 | SLC27A1 | STXBP2 | PARVA |
| DCTN4 | SLC4A7 | HOMER2 | SAFB2 | B4GALNT3 | PCNX4 |
| FAM20B | RPL23AP32 | KRIT1 | LHFP | ATG13 | RAC1 |
| MOB1A | ZNF654 | TUBB3 | CYYR1 | PIGT | NEFH |
| FOXO3B | DDX20 | S100A1 | CA3 | PACSIN2 | BICC1 |
| SNX13 | C5orf24 | HIST1H1C | TUT1 | MIR6837 | NSMAF |
| CCNB1 | STX17 | COL4A2 | DNAH1 | UACA | RBPJ |
| PRR14 | LARP1B | GCAT | SLC25A28 | LOC647115 | AKT3 |
| ZDHHC9 | GTF2F2 | TOMM22 | FRA10AC1 | LOC389332 | FCGR3B |
| ANP32A | NUDCD2 | CDKN2A | ACVRL1 | DGKA | MAP3K8 |
| ANAPC5 | RAP1GDS1 | BAG6 | FLJ20021 | SHISA3 | PGRMC2 |
| SELT | PSMD10 | CCDC85B | CHKB | ECE1 | LOC102724200 |
| UBE2S | LOC101930115 | PRPF4 | FZD4 | TMPRSS2 | TMX4 |
| RRM1 | ZNF792 | WBSCR22 | HYI | LAMTOR1 | DPYSL2 |
| SECISBP2 | LOC100996579 | CRIP2 | KCNIP2-AS1 | CRIP2 | RDH11 |
| GALNT2 | LOC101928433 | TRAF4 | RASL10A | NAA60 | PSMC2 |
| DCAF7 | C6orf223 | PKN1 | MAOA | EDC4 | ECM2 |
| RPS6KC1 | NDUFB4 | FIBP | ANKRD20A5P | FAM214B | RAB38 |
| PPP4R2 | DIS3 | CDKN3 | LINC00482 | SSBP3 | STK24 |
| PDE6D | SP3 | CXCR4 | RAD51-AS1 | MAP3K14 | RAB27A |
| TAF11 | SLC16A6 | RALY | LOC90834 | CTDNEP1 | METTL3 |
| ASAP1 | THAP2 | LOC100507855 | ND6 | PEX14 | AGTPBP1 |
| TUG1 | ZNF614 | CDK1 | SNORD68 | CD160 | H6PD |
| MFN1 | FBXO9 | MRPS12 | EHD2 | LOC100287525 | LGALS2 |
| DNASE2 | STAMBPL1 | CHMP1B | TNRC6B | RIPK1 | ACPP |
| ABI1 | SMYD2 | LAMP1 | HAX1 | ASAP3 | HNRNPDL |
| TTPAL | ZNHIT6 | LCN2 | ARHGAP30 | PTGDS | PDPN |
| RAB14 | SNORD77 | PFKP | LOC101930075 | ARF6 | STX2 |
| PSMD1 | LOC114224 | PLD3 | KIZ | CXXC1 | PYGL |
| FAM175B | C8orf59 | ATN1 | PLEKHM1 | NTN1 | KIF13B |
| MAP3K7 | NUTM2B-AS1 | ERI3 | GALNT16 | GDPD2 | IVNS1ABP |
| UBE2T | SCARNA13 | LOXL2 | TECPR2 | SLC25A23 | SLC2A5 |
| APH1A | ACVR2B | REEP5 | ALS2CL | SDHAP2 | TSC1 |
| ESRP1 | PLEKHA8 | ZNF467 | LOC728903 | INF2 | GALNT12 |
| MED6 | CBX3 | POLR2J | RIF1 | AZIN2 | RPL15 |
| MIA3 | RPL37A | ISG15 | ZNF528 | PFKL | ATMIN |
| HTATIP2 | MRPL30 | JAG2 | MAN2A2 | ADD1 | SNORD45C |
| OGFOD1 | ZNF519 | DEFB1 | MUM1 | MTMR14 | CUL3 |
| NRBF2 | PIK3CA | GLDC | GLIDR | TAOK2 | LGALS3 |
| CSTF1 | ZMYM1 | PRKCD | CLTA | CLK3 | ARL6IP5 |
| SYNCRIP | PBRM1 | MDK | OXTR | PADI2 | PAPSS2 |
| SMC6 | PPIL4 | LOC101930405 | LOC102724197 | THSD4 | ACOT9 |
| COPZ1 | ALKBH8 | UQCR10 | CBX7 | CA12 | TVP23B |
| CAPZB | FSD1L | RGCC | ZBTB47 | DCAF11 | NAP1L3 |
| N4BP1 | ZNF805 | HMMR | GRK3 | BOC | MET |
| CENPU | EIF5A2 | FUT8 | SYNM | ADAT3 | CASP7 |
| GTF2H1 | FAM208B | PSMB3 | TSPYL2 | PLP1 | RGS4 |
| NAPA | MIR3671 | LSR | GRAMD3 | TMEM115 | LEPROT |
| SLC30A5 | CCDC15 | ASS1 | ATXN3 | ZFYVE28 | BMP2 |
| UHRF1 | TMF1 | FARSA | RPUSD4 | PPP1CA | PROSC |
| PCMT1 | ATP6V1H | TOP2A | TMEM161B-AS1 | MAP2K2 | ID2B |
| SSR1 | KIAA1033 | SNRPB | GRASP | HAGH | RFC1 |
| HNRNPA2B1 | MFSD14A | DGKZ | CABP4 | TPCN1 | LIN7C |
| EIF2AK1 | SRSF10 | SF3B4 | PARVA | SRGAP1 | PCLO |
| RBM22 | FCF1 | CBLC | TCP11L2 | CPT2 | SNX3 |
| LOC101929597 | ACRC | FLNA | TBX18 | SH3PXD2A | NPTN |
| SMARCA4 | ZNF678 | SSB | ENPP7 | MFN2 | DENND1B |
| CCZ1B | IFT74 | BUB1B | IRF2BP1 | PBXIP1 | MED13 |
| DNAJB9 | KIAA1586 | GPS1 | ST6GALNAC6 | MMP28 | WDFY3 |
| PBDC1 | CHD2 | FAM193B | CYP17A1 | RNF185 | HECTD4 |
| GYG1 | MIR6516 | SHMT2 | TFE3 | SCN9A | ASH2L |
| CREB1 | SUB1 | LRRC32 | RAB2A | FAM83E | SART3 |
| ATP6V1C1 | METTL15 | PYCRL | SOCS1 | LITAF | NCBP3 |
| UBE2J1 | TAMM41 | BRD2 | MIR6883 | RAB7A | TXNRD1 |
| LAMP1 | CAMSAP1 | ADAP1 | SH2D3A | PKP3 | NT5E |
| TMEM167A | STAT3 | CCNB2 | CARMN | FUT2 | SIRPA |
| LYPLA1 | ZNF117 | BGN | SAA2-SAA4 | ACADVL | USP34 |
| SMC3 | OSGIN2 | INHBB | TMEM110-MUSTN1 | PLXNB2 | STXBP1 |
| DNTTIP1 | DNAJC10 | SPON1 | LOC102724870 | RAMP3 | AGA |
| WRAP73 | FGFR1OP2 | BACE2 | LOC101927870 | LPAR1 | NPEPPS |
| TPM3 | MCM10 | THY1 | RPS15A | HS6ST1 | PER2 |
| PRKDC | CACYBP | ATP6V0B | TAT | PXN | PPP3CB |
| NFYC | PLK4 | HMGB1 | TRIOBP | SMPD1 | THOC2 |
| CDC42 | FBXO11 | MFGE8 | HOXA5 | LOC101930168 | CCDC28A |
| MCL1 | BARD1 | RPL36A-HNRNPH2 | FAM219B | LRP10 | SRSF5 |
| INHBA | ZRANB2 | TACC3 | ATG2A | CIDEB | ARFIP1 |
| EPT1 | TTC5 | PRSS2 | C10orf54 | ZNF821 | CDC27 |
| CASP8AP2 | TTLL5 | SMTN | SRSF5 | FLJ42627 | C1RL |
| SQLE | TAF1D | ATG101 | CHRNB2 | AGPAT2 | PIAS1 |
| FBXO22 | ZFYVE16 | VEGFA | ZNF444 | SMAP2 | DPP4 |
| FKBP4 | KLF7 | RHOC | NR3C1 | DPF3 | YTHDC2 |
| TUBGCP4 | ZFC3H1 | FKBP4 | SPTLC3 | LRRN2 | FAM13A |
| DLG1 | ZZZ3 | EPB41L1 | ZNF205 | SYNPO | FAM8A1 |
| TMEM9 | ZNF644 | VWF | LOC102725108 | CEACAM3 | N4BP2L1 |
| CREM | PCMTD2 | HIGD2A | KIF26A | PRB1 | SPCS2 |
| CENPF | KDM4C | WDTC1 | CRY2 | BRMS1 | ACSL3 |
| MICAL2 | CSPP1 | RNASEH2A | MEDAG | PRKCZ | WNT5A |
| TOP2A | ZGRF1 | RRBP1 | CARMN | USP2 | MED14 |
| ETNK1 | METAP2 | IFI6 | SYCE1L | MMP24-AS1 | PTPRO |
| TMED8 | SREK1IP1 | TYMP | NFAM1 | CNOT3 | RUFY1 |
| ARPC5 | ERCC6L | MIR6787 | CEP68 | TMEM129 | BCLAF1 |
| PPFIA1 | YAF2 | RACGAP1 | ZNF554 | SLC27A4 | CLK1 |
| PPP1R15B | IER3IP1 | HIST1H2BC | RAX2 | ALS2CL | UBE2B |
| RRBP1 | MPP3 | PRCC | FAM13A | MYO15B | NBR1 |
| SOAT1 | ERCC6-PGBD3 | CRTAC1 | PELI3 | SPIB | NDNF |
| RAC1 | CDC40 | UBA1 | CTDSP1 | ARHGAP17 | PKN2 |
| FAM118B | ARID2 | EMID1 | MIR99AHG | SECTM1 | CTSL |
| NUCKS1 | ASPM | KRT17 | CCL15-CCL14 | SEMA6A | S100A10 |
| MPV17 | XPO1 | DCXR | PIK3R1 | XDH | ACAA2 |
| RPS10-NUDT3 | ANGEL2 | NCBP2 | DST | ZBTB43 | KAT2B |
| RMI1 | TAF15 | TAGLN | ANKRD29 | HLA-E | ZNF573 |
| LIG4 | ERGIC2 | KIF20A | SUN2 | LNPK | CHST15 |
| PRPF40A | MCEE | CDK2AP2 | CSDE1 | LZTS2 | FBXW11 |
| VAMP7 | MIS18BP1 | PTP4A3 | KIAA1683 | CLDN8 | HS2ST1 |
| SMARCA5 | MIR1248 | FOSL2 | MAGI2-AS3 | NECTIN2 | GCA |
| PRRC1 | LZTFL1 | SNORA21 | MAPKBP1 | LPP | LYST |
| DCLRE1C | BLZF1 | LYPD1 | RBMS3 | GAK | ETS2 |
| SETSIP | CDC14B | ATP6V1B1 | TBC1D13 | NXF1 | SERPINB9 |
| TMEM185B | POC5 | KLF6 | ZNF490 | URGCP | FEM1B |
| TSR1 | ASAH2B | PDIA4 | EPN1 | LEMD2 | KCNB1 |
| SPTLC1 | PDCD2 | NUP62 | ABCA8 | BABAM1 | SVEP1 |
| LLPH | TNNI3K | DLGAP5 | UBAP1L | SH3RF1 | C4BPA |
| UBFD1 | DDHD1 | CENPF | CCDC3 | FASTK | DNAJC7 |
| ACP1 | LRRC8B | PPP1CA | PALM2-AKAP2 | FADD | WDR19 |
| KBTBD2 | NGLY1 | INPPL1 | PDK4 | MYO7B | HADHB |
| PTPN12 | RCN2 | MTHFD2 | C19orf60 | VPS39 | ATP7B |
| NAA25 | DUSP10 | COX8A | HCFC2 | ASB8 | GPR137B |
| EIF4E | CARNMT1 | POR | PPARA | LPCAT4 | LAMA4 |
| RRM2 | NDUFB6 | UGT2B28 | PTRF | DOLPP1 | CD59 |
| ACTR3 | KLHL20 | CKB | BLCAP | EFNB1 | LHX2 |
| UBE2B | ERCC8 | LZTS3 | EDF1 | HGS | SCAMP1 |
| PLEC | ZNF561 | DNAJB1 | DGCR14 | RNF123 | WASF3 |
| PMS2P2 | AJUBA | COL18A1 | SLC25A27 | NAA40 | HBA2 |
| CDK5 | SDHAF4 | TNNT1 | RRP12 | ZZEF1 | TARDBP |
| R3HDM4 | PRPF39 | SCGB1D2 | LOC100507520 | TLCD2 | CPE |
| PRELID3B | TMEM161B | ASRGL1 | TK2 | ITGB4 | KIAA0430 |
| RAB18 | MIR636 | COX6A1 | LOC101929681 | CTDSP2 | APPBP2 |
| FARSA | CEP70 | CAPG | SPPL3 | STMN2 | FBXO38 |
| KDELR2 | PHF20L1 | MFAP2 | WDFY2 | SCNN1B | WHSC1L1 |
| GOLGA7 | NAA15 | TUBA4A | SETDB2 | ZCCHC14 | ANXA5 |
| TMEM189-UBE2V1 | ZNF670 | CTNNB1 | SYNE3 | IDH3G | MORC3 |
| ADNP | DTWD1 | SOX12 | AQP2 | PLEC | LOC145783 |
| BZW1 | NUTM2B-AS1 | BTG3 | PICK1 | CD177 | DIXDC1 |
| HOMER3 | LRCH1 | MCAM | RDH11 | CCDC12 | MAP3K5 |
| TMCO1 | THUMPD1 | CSNK1A1 | SLC12A1 | CTSD | FAM208A |
| API5 | RFX3 | PPIF | TBX15 | EFHD1 | PHF3 |
| ARPP19 | GTF2H2B | SORD | GABARAPL3 | DAXX | SLC35G2 |
| BGN | ZBTB26 | HIST1H2BH | FBXO11 | SP2 | SRSF10 |
| SERINC3 | CCDC122 | HSPD1 | CPED1 | MAFK | HAS1 |
| ME2 | ZNF782 | TNFAIP2 | EXOSC6 | DHTKD1 | MAOA |
| RAP2B | ZNF565 | SLC2A1 | ZNF445 | MAGI1 | VPS13C |
| PLAU | PHC3 | KIAA0101 | ACSS2 | IER2 | CASD1 |
| DCTD | NEBL | PRAME | ZNF436-AS1 | SMIM5 | SSPN |
| THUMPD3 | CCDC112 | MEA1 | TESPA1 | LDLRAP1 | PER3 |
| DCUN1D1 | PAPOLG | L1CAM | SNRNP70 | CFL1 | NR3C1 |
| PEX13 | XRCC4 | MLF2 | VWF | DEDD2 | CYP3A5 |
| PPID | COPS8 | LSM7 | CD59 | PPP2R1A | TMEM168 |
| ATP6AP2 | ZNF431 | IGKV1OR2-108 | DLC1 | NDRG2 | RUFY3 |
| LOC101930112 | DEPDC1B | BUB1 | NFIB | BORCS8 | ANOS1 |
| PRKCI | FNIP1 | PRC1 | DAPK2 | SLC35E1 | SLC25A36 |
| SLC52A2 | ATM | CKS1B | LYRM7 | AQP8 | HSD17B11 |
| ZNF146 | CAPRIN1 | TECR | VAPA | VASP | TBP |
| UBE2C | FRG1HP | PRKCI | PPP5D1 | PRRG2 | FBXO9 |
| TTL | PAXBP1 | MRPL42 | PINK1 | SDHA | MAT2B |
| SP110 | VPS35 | TMEM97 | IRX1 | PAFAH2 | IL6ST |
| CAPN7 | MASTL | CHCHD2 | XBP1 | ABCC3 | HBB |
| MRPL50 | RPS15A | S100A4 | R3HCC1 | DOCK5 | ITM2B |
| CRK | MEX3A | TLN1 | MIR6859-1 | LOC100996792 | PUM2 |
| L3MBTL2 | RSPH10B2 | SERPINH1 | LOC101929500 | SNX17 | PROS1 |
| CDC5L | EIF3M | UNC5B | MEG3 | ATP8B1 | CHN2 |
| KDM5B | HSPE1 | PBK | CCNL2 | TINCR | HERC2P9 |
| FANCI | ZNF292 | TMPRSS4 | ARL6IP4 | UBA1 | CHUK |
| RAB27A | LOC105372255 | CYBA | MB21D1 | BSG | RECK |
| PIK3CA | TRMT11 | SMC4 | CES2 | MALL | KIAA1109 |
| BCLAF1 | GPALPP1 | AURKA | CMTM7 | RELB | CAP2 |
| SFT2D1 | GMPS | SLC25A1 | LOC101929054 | KHNYN | RNF219 |
| CLDND1 | METTL18 | TKT | USP48 | KCTD5 | FAM134B |
| CARMIL1 | MCM3AP-AS1 | CTBP2 | BIN1 | ARHGEF18 | USP3 |
| EPRS | C11orf57 | APOE | ATP5SL | SNX1 | ATXN1 |
| OXR1 | BPTF | PDCD5 | BOC | TRANK1 | DHRS7 |
| LOC101060386 | HAUS6 | KDM6B | ARMC5 | ALPI | KHNYN |
| PCBD2 | ROBO2 | SDHC | ATP2A2 | PPP1R15A | GSPT2 |
| H2AFV | ZNF571 | TWF1 | SMARCC2 | MISP | NUDT9 |
| MIR3658 | RFESD | CLDN10 | LEPROT | MAPK3 | THUMPD1 |
| RAD23B | ZNF281 | NTHL1 | PRICKLE1 | CIRBP | PURA |
| PPP2CA | CEP57 | NDUFB11 | SPPL2B | SLC30A10 | SYPL1 |
| RNF115 | TAF1B | C19orf53 | S100B | LETM1 | EHBP1 |
| WBP11 | COX10-AS1 | SNCG | PTN | RHOG | CMAHP |
| PRC1 | KANSL1L | SCAND1 | PTCRA | PYM1 | MFF |
| LSM10 | HCG18 | COMP | SLC25A45 | PCDHGA1 | DKFZP586I1420 |
| G3BP2 | FGFR1OP | CARM1 | XAB2 | ABHD6 | ST13 |
| HSPA4 | RINT1 | PSAT1 | ITSN2 | UPF1 | ATP9B |
| KNTC1 | ALDH1A3 | H1FX | BCL2L11 | SNRNP70 | IPO5 |
| AGO2 | NFYB | CEP55 | KLHL21 | MYH14 | PAFAH1B1 |
| TK1 | GAS6-AS1 | FAM107A | NUTF2P4 | STAT3 | SNX7 |
| INPP4A | GTF3C3 | DDAH2 | FGF1 | PURA | BCHE |
| CNN3 | ANKHD1 | CHODL | NCR2 | MPND | CAND2 |
| COL12A1 | DCAF10 | BZW2 | HBB | CTNNA1 | SAMD4A |
| ALG13 | ZCCHC7 | MT1G | FOXP2 | RER1 | CPVL |
| NUCB1 | MIR15A | NDUFA3 | SFRP1 | NLRX1 | SRP54 |
| MRPL3 | KCNK17 | TSC22D2 | MYBBP1A | CORO1B | DCAF6 |
| DIEXF | RALGAPA2 | IRF9 | RBM10 | ATM | HNRNPA3 |
| DSG2 | ZCCHC8 | JUN | FBXO31 | LOC100505984 | GMFB |
| UBE4B | NUP58 | SSR4 | ALDH7A1 | PITPNA | RPL37A |
| LOC100506538 | ZNF780A | DUSP5 | STAT5A | ACADS | SACM1L |
| PICALM | VPS13C | COL15A1 | FBXL17 | ATG2A | SPG20 |
| PON2 | WNT2 | PSMB2 | LOC101929583 | PLEKHG6 | EIF4B |
| C1orf131 | CAND1 | GPM6B | LUZP1 | COPA | CDV3 |
| ATF6 | RBM39 | LSM2 | PTDSS2 | WBP2 | SON |
| TBCE | TBC1D8B | TGFB1 | PLXNA4 | CLDN3 | PCOLCE2 |
| SBNO2 | UBAC2-AS1 | COX7C | ATP5D | MIR6727 | MEF2C |
| CDK1 | THAP9 | MPZL2 | KRT14 | LTBP4 | PPM1D |
| DESI2 | NMD3 | GPI | KRT15 | CALM1 | PIK3C3 |
| PDLIM5 | IFNG | EPCAM | SCNN1A | HECTD3 | MPHOSPH6 |
| GALNT7 | LSG1 | DDTL | LOC101060179 | PHYKPL | TOX4 |
| FBXO28 | RBM12 | OASL | ZBTB16 | ENTPD5 | ADAMTS3 |
| DPCD | DIAPH3 | AKAP8L | ARHGAP19 | RPUSD3 | HLF |
| RIPK1 | FAIM | SH3BGRL3 | ESYT1 | MYO18A | BCL2 |
| NREP | OTUD6B-AS1 | SLC34A2 | ACAD10 | CHP1 | HDAC4 |
| PRKAR1A | TOPBP1 | PGLS | VAMP3 | C9orf64 | DNAJC8 |
| FASTKD5 | APOLD1 | AHCY | HBA2 | CHST5 | TSPYL1 |
| HSPE1-MOB4 | SNORD54 | YWHAZ | CIDEA | ZG16 | NAV3 |
| LMBR1 | LOC102606465 | CST3 | LTBP3 | ABHD4 | FCN1 |
| PPA2 | DCP2 | TUBB4B | TDRD3 | ABHD17A | CAV1 |
| PGP | CDK19 | LRIG1 | GNL1 | TNFSF12-TNFSF13 | SH3BP5 |
| EML4 | REV3L | FAXDC2 | ITGA9-AS1 | GDI1 | SAR1A |
| DTL | CELF1 | COL6A1 | SH3GLB2 | DGCR2 | GALNT11 |
| ZNF398 | VWA8 | FZD10 | TMEM72 | MCOLN1 | ADD3 |
| DPY30 | NUMB | HDGF | SPAG8 | MON1B | TUSC3 |
| MIR3652 | BRAF | CCNB1 | LOC401052 | DCLK1 | TNKS |
| HNRNPDL | TPMT | S100A14 | LGALS12 | SPECC1L | COX20 |
| TMEM70 | NUDCD1 | IFIT3 | HDAC5 | MGLL | C6orf120 |
| CCNL1 | PPAT | CALM3 | GNG11 | KDELR1 | ZNF106 |
| CTPS1 | LOC101927137 | MMP7 | ASB1 | NPAS2 | HERC2 |
| POLR2K | SOS1 | SNRPD2 | TCF25 | SH3BGRL3 | TLE4 |
| MIR4723 | ZNF12 | ZWINT | PDE7B | PRKCD | RAPGEF2 |
| UBE2L3 | ALG10B | ATP5I | LMBR1L | MBNL1-AS1 | TTC37 |
| VMP1 | TMEM67 | ATP5G3 | C19orf68 | LRP5 | EID1 |
| STAU1 | MACC1 | CDH6 | FIGN | TMEM222 | ATP2C1 |
| MALAT1 | ATP11A | VCAN | AKR1C1 | ZBTB7C | EPB41L4B |
| HIST1H2BH | TATDN1 | KRT23 | RARA | ARAF | SWAP70 |
| CANX | L3MBTL1 | LMNA | PQLC1 | CCL15-CCL14 | MIR22 |
| SLC33A1 | METTL6 | BMP7 | AGAP3 | TEX11 | TBC1D4 |
| TIMM23B | FRG1KP | SPINT1 | SETD3 | PLXNA2 | MBP |
| INTS6 | MKL2 | IFI27 | WASH1 | ITM2C | RHOQ |
| SIAH2 | IKBIP | LAMP3 | SF3A2 | CCDC61 | TMX2-CTNND1 |
| VAMP3 | CARD8 | THEMIS2 | CD248 | ATP6V0D1 | RBM39 |
| MED17 | C12orf73 | ENO1 | AKAP12 | CLEC16A | TIAM1 |
| PNO1 | ZBTB11 | P4HB | TMEM100 | MLF2 | PLIN2 |
| STX3 | PPA2 | SAR1A | CLU | PHACTR4 | CCNT2 |
| ARF6 | EIF1AX | MAL | RAB11B-AS1 | LOC101060181 | BCL2L13 |
| RARS2 | ZNF674 | COX6C | YY1 | C19orf24 | INPP5F |
| CTNNA1 | SLAIN2 | GAPDH | CAT | SNX9 | DPYD |
| PIGF | KNTC1 | VDAC1 | COQ8A | STAT6 | ANKRD10 |
| FBXO7 | UBR5 | VTCN1 | SAT2 | VGLL4 | VPS4B |
| NUP62 | EPB41L4A-AS1 | COL6A2 | TNMD | SFN | ANAPC13 |
| EBLN3P | CCT8 | CITED2 | MMAB | MAST2 | SPATS2L |
| RYK | ZNF230 | GPX4 | SCGB3A1 | DISP2 | ANKHD1-EIF4EBP3 |
| MIR1248 | NPIPA5 | PLTP | LOC101927137 | C6orf89 | PIK3R1 |
| CNOT8 | SRFBP1 | TRIM29 | RBM6 | TCF25 | VWA8 |
| LOC100506639 | MTCP1 | MEOX1 | NTRK2 | STX4 | FNDC3A |
| NECTIN2 | SHPRH | GADD45GIP1 | CTBP1-AS2 | CEACAM7 | ISOC1 |
| ARMC10 | HAUS5 | PRSS21 | DHDDS | C15orf38-AP3S2 | KIDINS220 |
| COPA | METTL5 | CAPNS1 | NFIC | HLA-C | LXN |
| BUB3 | BRWD3 | NR1D1 | GNG7 | ELF4 | IQGAP1 |
| KLHL12 | PPP1R2 | KLK5 | ECM2 | C8orf82 | ISCA1 |
| LAPTM4B | AHCTF1 | COL9A2 | LOC102724814 | WNK4 | IGFBP6 |
| GSTCD | SIX4 | ECT2 | CDKN2A | RCAN1 | CREBZF |
| RHEB | ZNF207 | ST6GALNAC2 | USF2 | ABHD11 | ZBED5 |
| WDFY1 | IQCB1 | SEPW1 | SPG20 | BEST4 | PRKACB |
| DPH3 | KIAA0485 | ITM2C | LOC100506558 | DHRS4L2 | EVI5 |
| ZNF638-IT1 | UFM1 | TRIB2 | HOTAIRM1 | MYOM1 | KIAA0355 |
| H2BFS | RBM26 | OXTR | ARL4A | ANXA11 | RTN4 |
| NUP58 | SLC25A27 | MIR7703 | FLJ41455 | VSIG2 | AGTR1 |
| RPN2 | RANBP17 | IGLL3P | FMO2 | PRMT2 | NEK1 |
| YKT6 | TNPO1 | RBM38 | ZNF595 | SLC44A4 | NGLY1 |
| KIF11 | BDP1 | FZD2 | COQ8B | ADH1B | ASPH |
| MCUR1 | ARL13B | HIST1H2BK | CD34 | FKBP1A-SDCBP2 | ADH1B |
| PEX2 | CALD1 | THBS1 | GOLGA8N | DDA1 | HEBP1 |
| PROSC | NADK2 | LOXL1 | CTSF | TMEM61 | CBX7 |
| SSH1 | MTFR2 | ISYNA1 | GDPD5 | RALB | DCTD |
| CDS2 | LOC101929356 | MT1HL1 | ACAA2 | OGN | IFI16 |
| C6orf62 | TPP2 | ZFP36L2 | LOC100133089 | MAEA | MDFIC |
| HNRNPAB | CEP83 | CEBPB | PCDH12 | FAM222B | DNAJC16 |
| LARP1 | EMC3 | COX5B | SYNPO | RNF213 | ZC3H7A |
| NCBP2 | C8orf37 | SPINT2 | LOC283788 | FUS | KPNB1 |
| NDC1 | UBE2T | ITGB4 | NDFIP1 | CES3 | SEPP1 |
| MCMBP | CDH11 | PTH2R | MIR6756 | BAG6 | NSG1 |
| MIR6787 | SRSF3 | IGLL5 | SRPX | TCF7L2 | IBTK |
| KIF1BP | NEK2 | RGS1 | IGF1 | TECPR1 | NR3C2 |
| TMEM183B | F2R | RPL10 | SNCA | RRAS | USO1 |
| XIAP | PPIL6 | IGFBP4 | ARMCX5-GPRASP2 | HES1 | NCOA1 |
| MRPS12 | LRRCC1 | MT1H | CDK10 | MICAL2 | TNFRSF10B |
| PEA15 | ALG13 | LAPTM4B | CRYAB | AAK1 | AFF1 |
| SLC25A32 | ANKIB1 | IGHA2 | JAM2 | CHGA | CERK |
| WDR26 | CEP128 | PART1 | RASIP1 | PLCD1 | DIAPH2 |
| KRIT1 | ZNF84 | CYAT1 | WDR81 | VPS13D | DCP2 |
| CGGBP1 | TAF1A | SEC61G | LDB2 | ACKR1 | FAM179B |
| PTK2 | RAB18 | NDUFA7 | ENGASE | TSPAN14 | ZBTB1 |
| ISG20L2 | ATAD2 | CXCL13 | MAPRE3 | LLGL2 | SLC38A6 |
| TMEM248 | RBAK | MIF | ELP3 | TEF | DHX29 |
| EXOC5 | LRRC58 | RPL36 | ZNF133 | SLC38A10 | INPP1 |
| GIT2 | PCMTD1 | INS-IGF2 | ZBTB40 | P2RX5-TAX1BP3 | MAGI2 |
| TP53RK | CUL2 | NME1 | CLDN11 | JOSD1 | FBXW2 |
| NEMP1 | RIPK2 | DNAH3 | EDN3 | RRAGC | MED13L |
| ITCH | MCTS1 | SCRIB | CDK14 | B3GALT4 | CLEC4A |
| PRKCD | TXNRD3NB | MT1F | HEBP2 | EPS8L1 | TRAK2 |
| PPP4R3A | DIS3L2 | KLK7 | LOC101930404 | DMAP1 | FAM120A |
| SMAP1 | HNRNPD | CENPU | BCL6B | LAD1 | ALDH1A3 |
| OSBP | LOC645513 | PGGHG | FAM208A | TPRG1L | SEL1L2 |
| ENO1 | ELAVL1 | INHBA | GLTSCR2 | PLD3 | PRKCA |
| CKS2 | CD86 | RPN2 | PLA2R1 | ADCY6 | SCN3B |
| CALR | SBF2 | MYO10 | LOC102723897 | AKAP13 | SHQ1 |
| LUC7L | CISD1 | PLPP3 | MDGA1 | AURKAIP1 | BDH2 |
| ERMP1 | TRMT10C | MAD2L1 | HSDL2 | NADSYN1 | GPRASP1 |
| PSMC3 | MBNL2 | NDUFA13 | FUT7 | KCNK10 | CSGALNACT1 |
| MYO6 | RIT1 | ACTG2 | MIR4746 | ZDHHC7 | FAS |
| CEP89 | HIST1H4E | SLC6A8 | PSMD6-AS2 | P2RX4 | SYNJ1 |
| STAM | UBA6 | PGRMC1 | ZEB2 | CD151 | DNAJB9 |
| COPB1 | ZBTB1 | HSPA1B | UACA | KAZALD1 | SIPA1L1 |
| STX6 | DCLRE1C | IGK | ARL17B | PDE9A | MAP2K4 |
| KHDRBS1 | NUP160 | MRPL13 | FAM149A | PRRG4 | ATRX |
| ZFAND3 | DCTN4 | PTPRF | SVEP1 | SESN2 | AMIGO2 |
| TIMELESS | USP24 | GSN | ZNF580 | ARFGAP2 | RALGAPA1 |
| ADSS | NDUFAF7 | IGLV1-44 | PELP1 | NAGA | LOC728392 |
| REL | GALNT2 | CD74 | ITM2A | DAGLB | IKBKAP |
| FBXO45 | ZBTB41 | MMP2 | CREBZF | GLTP | TASP1 |
| TPGS2 | SNHG4 | COX6B1 | TCF3 | GSN | THBD |
| CCNB2 | RRP15 | KRT8 | PDLIM4 | AP2A2 | SEMA6D |
| BHLHE40 | MIR17HG | IGKC | LOC100653057 | UBE2L3 | LYVE1 |
| CLINT1 | ANLN | IGLC1 | HEY1 | COG4 | ARL8B |
| DDX42 | C11orf31 | LGALS3BP | KIAA1656 | TAPBP | TGFB2 |
| NDE1 | ZXDC | MFAP5 | RASA4B | TNFRSF1A | LOC102724562 |
| YME1L1 | MYO10 | IGLJ3 | MAST4 | PPARGC1B | SLC16A5 |
| TXNDC9 | PUS7L | ARMC2-AS1 | PDGFA | MIR6734 | DLG5 |
| ADIPOR1 | PHF6 | LGR5 | TLN2 | S100A16 | YME1L1 |
| GSK3B | PSMC6 | IGFBP5 | RHBDD1 | CHMP1B | CCNG2 |
| MTFR1 | ICE1 | CNN3 | GYPC | GUCA2A | C1S |
| SLC11A2 | TIMM8A | IGKV1OR2-118 | C5orf45 | FZD5 | MCUB |
| ANKRD17 | CLASP2 | TPM4 | MCTP1 | LSR | SUPT7L |
| LEPROTL1 | LOC101927204 | TFAP2A | DCX | NFKBIA | GRB10 |
| ZUFSP | EEF1E1 | TXN | TBRG1 | GALM | SPTAN1 |
| LOC101928625 | TRIP12 | HSPB1 | CEP126 | INAFM1 | TJP2 |
| SPIN1 | TNFSF11 | DHCR24 | RAD54L2 | SURF4 | EPB41L3 |
| DNAJC3 | MYSM1 | IGF2BP2 | TEF | LDHD | EIF3L |
| SCP2 | NEMF | SST | PIGT | RXRA | FAM69A |
| RSRC2 | DLEU2 | HMGA2 | GATC | ARID1A | GRAMD1C |
| ANXA4 | WNT5A | CLU | FREM1 | C2orf88 | TOB1 |
| C12orf4 | KRR1 | APOA1 | THUMPD3 | ASCC2 | DMXL2 |
| PRMT6 | DDX50 | ZIC1 | MYLK | TMEM189-UBE2V1 | RBM12 |
| LRR1 | SPRTN | APOC1 | FADS3 | CAPN5 | SMARCA2 |
| SAMD1 | PHKB | OPHN1 | MIR1306 | RETSAT | SFTPD |
| B3GALNT1 | EMSY | IGHM | ZNF426 | MOGAT3 | GTF2H1 |
| LPGAT1 | BOD1 | MIR3620 | ZNF107 | GPRIN2 | PPP1CC |
| PGRMC1 | EXOC5 | ISLR | WIF1 | FAM63A | NTAN1 |
| TMEM216 | DNAJC27 | SFN | RAB3IL1 | C6orf136 | GNS |
| BUB1B | RNF219 | COL6A3 | ARAP3 | TNFSF13 | GOLM1 |
| SREK1 | ZFP30 | CRABP1 | CNPPD1 | TSR1 | MIR5047 |
| NPIPA5 | C12orf66 | RAB25 | PLPP1 | MEF2D | AP3S1 |
| HSPD1 | RC3H2 | FOSB | CCDC107 | SLC9A1 | TCEAL2 |
| SURF4 | REV1 | MUC16 | FRG1KP | PPARD | YPEL5 |
| MIR6741 | ARPP19 | LGALS1 | TNRC6A | LOC100129518 | BMP4 |
| TMEM50B | PATJ | PTGDS | MBD3 | LRRFIP1 | C2CD5 |
| C18orf8 | SENP7 | BST2 | UROD | C15orf39 | HSD17B4 |
| ORMDL2 | RILPL1 | SELT | SCARB1 | RNH1 | HMGCR |
| RPA1 | DDX18 | COL5A1 | MFGE8 | PDE6A | ZBTB18 |
| SCO1 | ZNF709 | MYH11 | CLUH | RNASEK-C17orf49 | TSPYL4 |
| ERBIN | CDC23 | SPP1 | TBC1D4 | FOXK2 | FKBP11 |
| GAPVD1 | MND1 | FBLN1 | PROS1 | RBM42 | RAP2B |
| EIF5A | G2E3 | MIR8071-2 | FAM107A | EXOSC7 | RCHY1 |
| ITPR3 | BRD7 | CXCL14 | SLC13A2 | FUT6 | ECI2 |
| RAP1GDS1 | FAM3C | MYL9 | JRK | HLA-B | ANKMY2 |
| MAGT1 | CDK1 | MLIP | PALM | ACAP1 | ANXA4 |
| GPR89A | ZNF567 | KRT7 | LOC100507547 | P2RY1 | PALMD |
| TM2D1 | ACTR6 | CHI3L1 | CARMIL3 | GADD45B | CAB39 |
| PPM1A | SRSF11 | LOC101060275 | CYP2A13 | BRD2 | PRKAR2B |
| SPHAR | SCAI | GPX3 | MSRB2 | DMPK | FGF9 |
| MAD2L1 | MORF4L2 | SLPI | DPY19L2P2 | PYY | RABGGTB |
| SLC39A7 | AGTPBP1 | CTGF | GFER | ETHE1 | OAT |
| C1orf27 | MTO1 |  | LOC105379655 | DUSP3 | SACS |
| TGIF1 | SEC22C |  | KIAA2013 | MIA-RAB4B | TMED7-TICAM2 |
| CFLAR | USP46 |  | EIF3L | ID3 | PDZD8 |
| FARP2 | C17orf75 |  | CAV1 | CDHR5 | SLC25A44 |
| GON7 | GABPB1 |  | PPIE | OCEL1 | PTGIS |
| MTX2 | LINC00662 |  | ZAK | MIR4640 | DDHD2 |
| ZNF318 | ZNF493 |  | ALG9 | SST | CFH |
| TFDP1 | PSMB1 |  | TBC1D25 | CCDC85C | OTUD4 |
| OAZ1 | AP5M1 |  | WDR48 | LOC101929340 | CALD1 |
| TNKS2 | LSM5 |  | VSTM4 | FRMD3 | ADAM9 |
| IPO7 | RECQL |  | SHE | NMNAT1 | DIRAS3 |
| TTYH3 | SUCO |  | RGL1 | RAPGEF1 | RAB22A |
| ANGEL2 | BBS10 |  | SLC16A7 | NR2F6 | FZD1 |
| MAX | C7orf25 |  | ABHD14B | COG7 | LRRC1 |
| HAT1 | UBE2B |  | LOC100288911 | SEC24C | GRSF1 |
| TBC1D7 | AMMECR1L |  | PIK3CA | BTD | PCDH17 |
| FTSJ1 | GUF1 |  | PRKAR1B | SMPD3 | TXNDC15 |
| RBM12B | GALK2 |  | MTUS1 | PROSC | LIPA |
| SUDS3 | LINC01558 |  | TM4SF18 | FAM219B | PIEZO2 |
| EFCAB14 | NUF2 |  | CHD9 | MARVELD3 | ATXN7 |
| TMEM230 | USP48 |  | IGSF10 | CKB | RNASE4 |
| NUP210 | TOP2B |  | RBL1 | CNKSR1 | PDZD2 |
| NUP153 | IFT80 |  | DDX54 | NUDT16 | TRA2B |
| SMIM7 | SCARNA15 |  | MCAM | ZDHHC12 | TNFAIP8 |
| RITA1 | RFC3 |  | YIPF4 | KLF6 | ROR1 |
| KPNA2 | KIF14 |  | ESAM | ID2-AS1 | PKD2 |
| PREP | NAE1 |  | POLR2J2 | PPP2R3A | PPP2R5C |
| ANP32E | KIAA1524 |  | FAM134C | LGALS3BP | SHTN1 |
| FAM96A | ATG3 |  | LOC643733 | CCDC107 | CAV2 |
| P2RX4 | PTPRG |  | MEOX1 | SGSH | ADCY9 |
| MIR6834 | TIMM17A |  | CMAS | ATP13A1 | LPAR1 |
| MTO1 | EFHC1 |  | RER1 | FUT3 | FBXL7 |
| H2AFZ | ORC3 |  | ELMOD2 | TRAPPC1 | BACH1 |
| EFR3A | SRSF7 |  | SMDT1 | SCUBE2 | FLRT3 |
| CSNK1D | FBXO7 |  | WHAMMP2 | PRDX2 | PMP22 |
| DONSON | CAMSAP2 |  | TPT1-AS1 | ECHDC2 | GABARAPL1 |
| PHF23 | ASAP1-IT1 |  | EXOC3 | TPSG1 | TM2D3 |
| YIPF6 | UBE3D |  | C6orf89 | FHL1 | ABCA8 |
| FEN1 | MITD1 |  | CCL28 | TMEM72 | PICALM |
| ENOPH1 | ABCB10 |  | KIAA0141 | KLHDC3 | SYNRG |
| STRN | CUL4B |  | PAF1 | CLTB | ICE2 |
| PRUNE1 | BUB3 |  | LINC00663 | JUP | TRMT1L |
| CELF1 | CEP78 |  | ACADVL | WIPF2 | LRRFIP1 |
| RNF146 | ZNF107 |  | MRC2 | SLC17A5 | LOC101930416 |
| URI1 | PTTG3P |  | LIFR | TMEM8A | QKI |
| SCARB2 | PRKAA1 |  | PDE3B | ADAP1 | SLC39A6 |
| ZBED4 | LOC102724870 |  | ZBTB3 | DNM2 | SPG11 |
| FLAD1 | ALPK1 |  | EIF4E3 | CIB1 | ADH5 |
| BSG | RBBP4 |  | GPATCH8 | ALAS1 | IL1R1 |
| TACC3 | DEPDC1 |  | HOXD4 | CPNE2 | TBC1D9 |
| PPAT | RLIM |  | MTMR9 | ZNF721 | GHR |
| MIS18BP1 | POLR1B |  | FTO | CTSA | ANXA3 |
| STAM2 | CENPF |  | RUNX1T1 | AKIRIN1 | CAPN7 |
| MR1 | TADA1 |  | MIR205HG | ABL1 | HECA |
| ILF3 | RPL23AP7 |  | NDN | RAPGEFL1 | SOBP |
| COPB2 | RAB3IP |  | TFAP4 | PITPNM1 | GNAI1 |
| TM9SF1 | THUMPD2 |  | EIF4EBP2 | CLN3 | MACF1 |
| PAXIP1 | NEMP1 |  | TXN2 | MRPL41 | ADGRG6 |
| SRD5A3 | CEP152 |  | WDR27 | HEG1 | UBE2G1 |
| XPO5 | ZNF267 |  | DPYSL2 | TMX2-CTNND1 | ZNF302 |
| MED8 | SLC39A10 |  | VIPR2 | SELENBP1 | CROCCP2 |
| VCAN | EFCAB7 |  | LOC729218 | LINC01137 | SORBS2 |
| SLC20A1 | PMS1 |  | IGH | LYPD8 | CDK17 |
| TPRKB | ZNF700 |  | LOC101930531 | SLC25A25 | C3AR1 |
| AP3M1 | ZNF639 |  | HOXA10-HOXA9 | FAM102A | LIMCH1 |
| PTER | SLC35F5 |  | SASH1 | ARL17B | LOC101930531 |
| GJB2 | THUMPD3-AS1 |  | GALNT15 | BCL7B | GNG11 |
| DDX19A | CCT6P1 |  | APCDD1 | ARHGAP21 | ACACB |
| NBR1 | C1GALT1 |  | C17orf51 | BLOC1S1 | DCLK1 |
| SEC61A2 | CDC73 |  | WDR13 | EHD4 | INSIG2 |
| GHITM | NAA25 |  | ZGRF1 | C8G | CYB5A |
| CKAP2 | XPNPEP3 |  | DAP3 | C19orf43 | ARHGAP6 |
| DPY30 | LOC101929777 |  | LOC654342 | KLF4 | OSBPL9 |
| FGFR1OP2 | RSRC1 |  | JAG2 | N4BP1 | HNRNPH3 |
| NET1 | ZNF121 |  | PRB1 | CPTP | SLC30A5 |
| SRP54 | ZNF268 |  | STAT5B | PHLDB3 | SMAD2 |
| CTDSPL2 | ZNF736 |  | FOXO1 | EPHX1 | TRAF5 |
| IRF2BP2 | TFB2M |  | CX3CL1 | APLP2 | SCP2 |
| CAND1 | SRPK2 |  | BIVM | MBD6 | LOC100507577 |
| COQ10B | USP6NL |  | PPP1R1A | DNAJC4 | MIS12 |
| TRA2B | NUP62CL |  | ATP6V0E1 | DDAH2 | ATF1 |
| PSMD10 | TFRC |  | MEGF8 | NSFL1C | DMD |
| TGFB1 | HAUS1 |  | TTLL3 | LARS | BAMBI |
| RNF14 | WDR76 |  | LHX6 | TRIM14 | ANGEL2 |
| ZFP36L2 | PEX13 |  | MRAS | FLII | CCND2 |
| MRPL4 | C1orf27 |  | DICER1-AS1 | RALBP1 | ATP2B1 |
| GOSR2 | POT1 |  | RPL18 | TMEM127 | REV1 |
| TOP1 | MANEA |  | DNAH7 | GBAP1 | HTATSF1 |
| CD58 | DDX21 |  | ZNF337 | ARRB1 | MEOX2 |
| RAB8A | DIEXF |  | FZR1 | DHDDS | IKBKB |
| CD164 | CNTNAP2 |  | ECHDC2 | CCS | PLCB1 |
| UBR5 | TRMT6 |  | LOC101060405 | THTPA | ZBTB20 |
| MCM4 | TRMT5 |  | EHBP1 | GDPD3 | ATP11B |
| CPD | CENPC |  | LINC00893 | SLC10A3 | SEH1L |
| ITGB1 | PIGK |  | MAPK3 | MLXIP | YLPM1 |
| FANCF | ADGRE2 |  | ABCA5 | RHOV | INSR |
| PPP6C | IPO5 |  | RERGL | EHD1 | AKAP9 |
| FLVCR1 | RPL36A-HNRNPH2 |  | THAP6 | TJP3 | SF1 |
| HIP1 | SEMA4D |  | LOC101930400 | MAF1 | ITPR2 |
| TAF4 | NOP58 |  | TEX35 | SH3GLB2 | FANCL |
| SDF4 | NSMAF |  | RBM5 | SULT1A1 | BPTF |
| CAPN15 | NEDD1 |  | FLVCR2 | CDKN2B | MAP4 |
| MAGOHB | SNORD89 |  | SMIM10L2B | FBXO32 | LUC7L3 |
| ATF7 | RPS24 |  | NUP214 | ALDOA | COLEC12 |
| LARP4 | POLK |  | CNN1 | AGRN | TERF1 |
| AZIN1 | OTUD6B |  | XRN1 | KIAA1522 | B2M |
| RBBP4 | SGIP1 |  | NCAPH2 | HSPG2 | SLC7A8 |
| ARPC2 | IRAK1BP1 |  | KLC1 | C2orf40 | SEMA3C |
| BLOC1S6 | DPM1 |  | VAT1 | MFSD5 | STXBP3 |
| MARCKS | ARL1 |  | OSBPL5 | UPP1 | ATP6V1D |
| TIA1 | RASAL2 |  | RPL22 | RAB8A | ATP5A1 |
| SYPL1 | HNRNPU |  | C14orf1 | LOC101927157 | LOC101929500 |
| CNIH4 | GRPEL2 |  | BHMT2 | TRPM4 | HSDL2 |
| SMC2 | CASP8AP2 |  | SLK | MOCS1 | SUN1 |
| WTAP | HAR1A |  | NPR2 | MINOS1-NBL1 | ADRA2A |
| PRRC2C | GTF3C4 |  | APLP2 | CCL19 | ZNF395 |
| ZMYND11 | GCA |  | PAQR7 | DAB2IP | GHITM |
| BICD2 | CCDC82 |  | ZNF579 | SLC4A5 | ATP5L |
| HNRNPR | ZMYM4 |  | WDPCP | B3GNT7 | ANK3 |
| NFATC3 | MPHOSPH9 |  | SMG6 | PGPEP1 | STX12 |
| RASA1 | NFE2L3 |  | GSTM1 | MTHFR | NR2F1 |
| CDS1 | RPL26L1 |  | CHDH | ACAA1 | GCC2 |
| CREBZF | MFN1 |  | FLYWCH1 | PSMD3 | RUBCN |
| SDAD1 | SLC2A8 |  | CACHD1 | FLNB | CHMP5 |
| FH | GPRIN3 |  | KIF1C | MFAP5 | TCF4 |
| BRI3BP | ZNF330 |  | CNPY4 | NR3C1 | SCG5 |
| SAYSD1 | MTMR6 |  | OVCA2 | LOC101930112 | KLF11 |
| DNM1L | HOMER1 |  | PKDCC | TSPAN1 | ZNF226 |
| UBE2W | BBIP1 |  | CLIC5 | LOC100507520 | TCF21 |
| BCOR | HBS1L |  | GTF2IP12 | ILK | SETSIP |
| DLAT | SNRNP48 |  | SNORD32A | F11R | TRA2A |
| TOB1 | C9orf72 |  | GCN1 | CPT1A | GLIPR1 |
| SHC1 | ANKRD26 |  | TF | SPINT1 | C1GALT1C1 |
| AP5M1 | WDR19 |  | RNF150 | NTRK2 | ZFAND6 |
| NME6 | FAM49B |  | PLEKHA4 | GBAP1 | SLC35A1 |
| GMCL1 | CLCN5 |  | STK40 | TMC4 | IMPA1 |
| ERGIC2 | ZNF680 |  | TRIM38 | MKNK2 | MDC1 |
| E2F5 | JMJD1C |  | EPC2 | MEP1B | RCBTB2 |
| TMEM135 | C12orf29 |  | ZCCHC24 | TMEM140 | DOCK4 |
| ATP6V0B | INVS |  | HAUS5 | ATP5D | PCMT1 |
| RAD54B | MIR155 |  | GLTSCR1L | MIA3 | FERMT2 |
| UBE2A | ALS2 |  | LOC101929177 | CA2 | ALDH2 |
| AURKA | TUG1 |  | HP1BP3 | MICALCL | EIF5 |
| BIRC6 | CEP63 |  | RUNDC3B | ATG9A | MYRF |
| NUDCD1 | NHLRC2 |  | CREBRF | MS4A12 | WDR47 |
| PRMT3 | ARL3 |  | LOC101930591 | PTK6 | SRSF1 |
| DAGLB | ARHGEF12 |  | KMT2A | HSD11B2 | SULF1 |
| NFATC2IP | SYNCRIP |  | ARRB1 | DHRS11 | UPF3A |
| ANKIB1 | KIF3A |  | ELMOD3 | HLA-F | PTCH1 |
| HEATR1 | ATL2 |  | LOC647070 | CCL28 | PTGER4 |
| ADPGK | AIMP1 |  | SLC7A6OS | FRAT1 | RBFOX2 |
| SNAP23 | PIBF1 |  | PID1 | TRAF3IP2 | NME7 |
| PARP6 | RAD51B |  | PAPPA | ITGA3 | KRR1 |
| RPAP3 | CENPJ |  | NLRX1 | CENPB | EFNB3 |
| SLC25A43 | C1orf112 |  | NXPH3 | CLIC5 | DPP8 |
| USP14 | PDPN |  | LRRC37A4P | SHB | PIN4 |
| RAD21 | ATXN1 |  | THYN1 | EXT1 | ATP7A |
| UXS1 | GTF2H1 |  | MORN1 | MPRIP | P2RX5-TAX1BP3 |
| ATP6V1G1 | DROSHA |  | PTGDS | HRAS | THAP9-AS1 |
| TBL1XR1 | DIMT1 |  | TMEM91 | CDKN2B-AS1 | FUBP3 |
| KIF23 | TRMT61B |  | RXRB | PARM1 | UPK1B |
| AP2B1 | RB1CC1 |  | NOD1 | TOB2 | REEP5 |
| HNRNPU | PHF14 |  | UGP2 | MIR4680 | FHL1 |
| ELMO2 | NXT2 |  | AUTS2 | SHROOM3 | GABARAPL3 |
| GART | TIGD2 |  | C2orf81 | IL10RB | FBXO3 |
| DERL1 | UMAD1 |  | POLL | CLPTM1 | PSMB1 |
| MRPL13 | CEP95 |  | FAM49A | CITED2 | CYBB |
| TMEM97 | LPGAT1 |  | LOC101927166 | ETFB | STAG2 |
| SRFBP1 | FAM149B1 |  | ISYNA1 | VPS37B | HMGN4 |
| METTL8 | TSEN15 |  | CYB5R3 | RERGL | MGEA5 |
| ARV1 | UBE2V2 |  | MZF1 | GLOD5 | UNC119B |
| SLBP | USP31 |  | UNC119B | SCNN1G | UBE3A |
| GLUD2 | DNAJC2 |  | FBXL5 | ZFP36 | RBM25 |
| LOC101929087 | PREPL |  | TCTN1 | RAB40C | CBR4 |
| AGAP1 | CEBPZOS |  | PBXIP1 | CDK11A | NEK7 |
| PIP5K1A | HNRNPA1 |  | PPT2-EGFL8 | ISG20 | CLIC5 |
| HMGCR | ERCC4 |  | MUC5AC | TEP1 | PIGK |
| KPNA4 | LOC728613 |  | TRIM56 | OAF | DPY19L2P2 |
| C12orf73 | HAS2 |  | EEF1D | HDHD3 | TGFBR2 |
| ARHGDIA | TOMM5 |  | ANKRD35 | GDE1 | SOS2 |
| RNASEH1 | BTAF1 |  | DEFB124 | RDH13 | ABAT |
| NONO | STOX1 |  | KLK3 | MIR6883 | CSF2RB |
| ERO1A | ZNF200 |  | CTDNEP1 | CDC42EP5 | CHMP2B |
| EIF4E2 | PMAIP1 |  | HNF4A | RIPK3 | PBX3 |
| DSCR3 | AASDHPPT |  | WDR6 | AOC1 | ZNF83 |
| MELK | PEX3 |  | WIPI2 | ARPC1B | BTF3 |
| RPP14 | LOC101060521 |  | POLR2F | RABGGTA | KCNJ2 |
| FKBP14 | LOC101929378 |  | CDS2 | MIR6859-1 | FAM129A |
| GRB2 | KIAA1429 |  | LOC101930071 | GAREM1 | UGP2 |
| FAM210A | CNTRL |  | TNPO1 | TLE3 | RRAS2 |
| WDFY3 | PTBP3 |  | MAML2 | SMAD1 | NFIB |
| PTBP3 | GPR19 |  | SGSM2 | ANKRD9 | FAT1 |
| LYN | ARNTL2 |  | LRP1 | EDEM1 | PJA2 |
| COG2 | ESCO2 |  | RIN1 | EMP1 | NCF2 |
| TUBB2B | RPUSD4 |  | GNA11 | ZYX | ZCCHC8 |
| LRRC59 | ARHGAP11A |  | MIR6890 | CYSTM1 | PTPN13 |
| YWHAB | TRNT1 |  | ZNF300P1 | DHRS9 | CAMSAP2 |
| RAB2A | SPDL1 |  | MYZAP | LMAN2 | IRS1 |
| RBFOX2 | DCAF17 |  | KAT2A | SERTAD1 | PPIP5K2 |
| TRA2A | PPWD1 |  | DCTN2 | MIGA2 | DRAM1 |
| PAIP2 | GLIPR1 |  | ZNF471 | EPAS1 | SNORD14D |
| CBWD7 | ZNF204P |  | CDO1 | IL6R | ITGAV |
| VAMP4 | C11orf58 |  | PCNX2 | MUC2 | DAAM1 |
| CCNG2 | SGO2 |  | SPAG7 | UBAP2L | KDM5B |
| PPP6R3 | ZNF37BP |  | STX17 | PSAP | ARID4B |
| MGAT4B | RLN2 |  | FAM184A | HAND1 | EIF1B |
| ZNF746 | LOC100190986 |  | MIR4800 | RHBDD2 | MTSS1 |
| LOC101927673 | MED23 |  | METTL7A | TMEM100 | PPFIA1 |
| TRIP13 | EPM2AIP1 |  | CYP4F12 | TGOLN2 | BTN3A2 |
| MRS2 | ZNF451 |  | FAM228B | NCOA1 | OARD1 |
| CXorf40A | KPNA5 |  | ADAMTS10 | TNFRSF21 | ZNF277 |
| KPNA3 | SLC33A1 |  | ANKZF1 | LMNA | CFDP1 |
| CAPRIN1 | CLINT1 |  | ANGPTL4 | MARCKS | ALG11 |
| TPP1 | FBXO5 |  | SNX1 | LOC729966 | SYNE1 |
| ASPM | TXNL1 |  | RUSC2 | APBA1 | VSIG4 |
| RBM17 | UBA5 |  | CIC | DGKZ | RBM26 |
| RNF126 | ATP5C1 |  | ERCC5 | CSRP1 | CKAP4 |
| C1orf56 | CCT6B |  | LAMA5-AS1 | ABHD14A-ACY1 | SYNE2 |
| IMPAD1 | ZFAND1 |  | GIGYF1 | C16orf58 | BTN3A3 |
| SSX2IP | LEMD1 |  | APOPT1 | MICALL1 | PNMAL1 |
| RPAP2 | FSBP |  | LAMB2 | PTPRF | SERINC5 |
| RCN2 | ZNF891 |  | POLR1B | NAPA | HNMT |
| ZCCHC10 | LRP8 |  | PIGL | TAGLN | ROBO1 |
| TMCO3 | MAP3K8 |  | SKI | VPS9D1 | SFPQ |
| BCL2L1 | SNRPD2 |  | PHLDB2 | USP9X | TGOLN2 |
| UBE2F | NKD2 |  | VRTN | SLC37A1 | IL13RA1 |
| DUS4L | NKRF |  | MEOX2 | ECH1 | MIR4680 |
| LOC101060399 | INTU |  | C2orf88 | COPE | KYAT3 |
| CSRNP2 | LRIF1 |  | TMEM37 | SMIM6 | UTP3 |
| BAIAP2L1 | TTC27 |  | FAM13A-AS1 | MIR6513 | HSPA2 |
| NUTF2P4 | CXCL2 |  | BRWD3 | STPG1 | TIA1 |
| NEK6 | TMA16 |  | MPHOSPH8 | RRBP1 | VLDLR |
| DDX39A | CENPK |  | CEP120 | PRR5L | SDC2 |
| PTPN3 | ABCE1 |  | CC2D1A | ACAA2 | PIK3R4 |
| PGD | ARL17B |  | RAMP2 | ZNRF1 | SEC24B |
| MRPS10 | CENPE |  | FAM109A | DNAJC22 | NFE2L2 |
| NOTCH2 | ESF1 |  | NOXA1 | CDK13 | HPSE |
| AP1AR | CTTN |  | HABP4 | CAND1 | SNRK |
| UBE2Q1 | TRIM24 |  | VAMP2 | B3GALT5 | SKP1 |
| ZNF259P1 | BHLHB9 |  | CSRP1 | PIGZ | MUT |
| FOPNL | MED4 |  | AMOTL1 | XIAP | COL4A5 |
| NEU1 | TRAPPC13 |  | COX11 | MMP15 | EPB41L2 |
| TOR1AIP1 | EXOSC8 |  | CNRIP1 | ANO10 | SYNE3 |
| PCBP1 | MRPS17 |  | HOXA7 | MAN2B1 | CHORDC1 |
| RYBP | CDK2 |  | STX8 | CEACAM1 | TCEAL4 |
| YDJC | SBNO1 |  | PRX | HBA2 | ELOVL5 |
| MTHFD2 | MTF2 |  | KLHL31 | CLCA4 | ANXA1 |
| ASPSCR1 | TGFB2 |  | RAB28 | TMEM120A | PRNP |
| RBBP5 | C12orf60 |  | RPL7AP10 | TOX | KLC1 |
| NBPF25P | WDR11 |  | INF2 | PI4KAP1 | FOXO1 |
| ZNF586 | MTRF1 |  | NUDCD2 | ACVRL1 | MAOB |
| ASF1A | WDR36 |  | AQP1 | ZYG11B | NAT1 |
| CXCR4 | PSPC1 |  | CYGB | DOPEY2 | SMARCA1 |
| C7orf25 | SLC30A5 |  | CBX6 | NBL1 | KLF10 |
| NRBP1 | SETDB2 |  | HSPA12B | PEX26 | UBE2J1 |
| LSG1 | ZNF22 |  | AIFM2 | PPP1R16B | CCDC47 |
| NUBP1 | CHUK |  | SELO | DDX41 | USP7 |
| ZMYM6 | RANBP2 |  | KANSL1 | IL32 | OSTM1 |
| CHD1 | VCAN |  | CCDC50 | MT1X | SAT1 |
| HM13 | TBC1D4 |  | SLC25A26 | RIMKLA | ATP1B1 |
| IER5L | CWC27 |  | ZNF395 | CC2D1A | NEBL |
| AKTIP | SKA2 |  | ZNF287 | CALM3 | AGL |
| ARF3 | TRIM59 |  | HOOK2 | WDR78 | OGN |
| PGGT1B | AKIP1 |  | ZNF37BP | GIT2 | BNC2 |
| DYRK2 | UNC93A |  | HNMT | SLC44A2 | RIOK3 |
| ENC1 | ZNF367 |  | SORBS2 | CCDC124 | RARRES1 |
| PARP1 | ZDHHC21 |  | TM9SF3 | SLC6A10PB | FBXO28 |
| ANAPC10 | TMEM182 |  | ENPP2 | FAM47E-STBD1 | APIP |
| TMEM50A | PPFIBP1 |  | COMMD6 | TP53INP2 | FBXL5 |
| CDKN3 | RPAP3 |  | DTNB | IGHM | CDC42BPA |
| LEO1 | HIST2H4B |  | GAS7 | ST14 | EVI2B |
| ERGIC1 | CSE1L |  | MIR24-2 | CARD10 | GOLGA8N |
| PAK1 | ZNF627 |  | EPB41L4A-AS1 | ARRDC1 | RAP1A |
| CDH11 | FIP1L1 |  | EGFL7 | RHOF | TNXB |
| MARK2 | RANBP6 |  | TNPO2 | PRPH | HS3ST1 |
| DICER1 | HSF2 |  | FOXP1 | TPM1 | SRSF7 |
| SUMO1 | KIAA1549 |  | SH3BP5-AS1 | TMEM54 | SMCO4 |
| ATRX | CCDC14 |  | ITFG2 | FLVCR2 | CDC42EP3 |
| ND2 | STRAP |  | CCM2L | TXNIP | DNAJA1 |
| SMCHD1 | MRPL32 |  | APOD | HNRNPUL2-BSCL2 | ATG5 |
| RANBP2 | AGFG1 |  | DUSP22 | BTNL8 | FGL2 |
| SLC25A11 | TMEM161B-AS1 |  | RORA | SPPL2B | CFHR1 |
| CUL1 | ZFP69B |  | FCF1 | SPATA20 | GATA6 |
| SNX2 | RFX7 |  | TPP1 | NUP62 | HACD1 |
| USP47 | LOC730102 |  | EPC1 | SCRG1 | NAA16 |
| SPCS2 | ATP13A3 |  | NEDD4L | GTF2H3 | SH3BP4 |
| IFNGR1 | MAD2L1 |  | FLJ10038 | DHRS1 | CD53 |
| TFAM | COIL |  | KIT | CES2 | ARMCX3 |
| CCNA2 | MPHOSPH10 |  | MAP3K3 | ACACB | TMOD1 |
| HN1 | HGF |  | ABLIM3 | TMEM171 | ABI1 |
| PBK | PDE7A |  | FAM212A | PDXP | PNMA1 |
| ASCC3 | ZUFSP |  | CCL21 | TRPM6 | LEPROTL1 |
| DEGS1 | ZNF92 |  | LGR6 | UBE2M | GATA4 |
| ERH | SLF2 |  | C16orf89 | MUC12 | MFAP3L |
| NUFIP2 | GINS3 |  | ATG7 | CASQ2 | RDX |
| COMMD10 | CMSS1 |  | ZNF252P | MICAL3 | PTPRC |
| VEGFA | OSBPL8 |  | EHD4 | TUBB3 | NDN |
| VANGL1 | DNAJC19 |  | CTIF | METRNL | TRO |
| PDIA4 | FBXW7 |  | RP9P | SHE | LOC101927705 |
| SPEN | TMEM237 |  | AARS2 | AATF | MEF2A |
| UQCRC2 | GPCPD1 |  | LIPJ | CLCN2 | CYP2B7P |
| PDS5A | PLGLB1 |  | MAPK11 | LOC101927705 | ACSL1 |
| SMS | FIGNL1 |  | VIPR1 | PPP1R16A | RPLP0 |
| CERS6 | POLI |  | HECA | TMPRSS4 | MYCBP2 |
| FKBP11 | SMC2 |  | MMP28 | MIR6824 | AVL9 |
| UEVLD | FKBP7 |  | SAMM50 | HSD3B2 | CHD9 |
| ZC3H7A | RFC5 |  | C10orf128 | FUCA1 | SNORD23 |
| MITD1 | CYP39A1 |  | ATP6V0A1 | MIR6884 | ODC1 |
| NSFL1C | HNRNPR |  | OAF | PTPRH | MFSD1 |
| CDK7 | EIF3E |  | PLCD1 | KIF13B | SQRDL |
| EXOSC3 | TRIM59 |  | SENP7 | YME1L1 | MS4A6A |
| FPGT | RRM1 |  | SCAP | CALCOCO2 | PYROXD1 |
| IL17RC | LSM8 |  | LINC00957 | SGK2 | RWDD1 |
| USP10 | NUP155 |  | TAC1 | RIOK3 | PLA2G4A |
| PIK3R4 | ENPP5 |  | GPC3 | UQCRC1 | RAB31 |
| EBP | CCNJ |  | TAF1C | OSBPL7 | CLIP1 |
| DESI1 | COL3A1 |  | MYLK3 | PRSS8 | C3 |
| PPP4R3B | TRIM5 |  | ZNF592 | TMEM37 | GNG10 |
| HN1L | MAP3K1 |  | PHF7 | MRPL38 | NDUFA5 |
| GLRX3 | TBC1D31 |  | TRMO | MXD1 | UQCRC2 |
| TXLNA | ACTL6A |  | CDH5 | MXI1 | WDR11 |
| ANLN | UTP15 |  | LAMB3 | SMCO4 | CETN3 |
| TM9SF2 | ZFP1 |  | STK11 | RNF126 | ABCD3 |
| ENAH | ZNF518A |  | FBXW7 | OTUD7B | EPS8 |
| OCIAD1 | LINC-PINT |  | NLGN2 | AMIGO3 | SPAG16 |
| TYMS | OSBPL3 |  | POLH | H2AFY | ZNF451 |
| ASAH1 | RPS7 |  | CUL9 | SLC25A11 | TJP1 |
| LINS1 | PHF10 |  | GABARAPL1 | SUGCT | CRNKL1 |
| MLX | ASUN |  | TXNDC15 | SEMA6D | PRRC2C |
| PRPF38A | CHD6 |  | TCF7 | GAS6 | COPS2 |
| ABT1 | SSX2IP |  | ARGLU1 | KIF26A | HEXB |
| TFAP2A | CLCN4 |  | RBM7 | APOL6 | C5orf15 |
| RUFY3 | ASB9 |  | ABI2 | TRIM47 | SYBU |
| PRPF18 | C2orf49 |  | MYBPC1 | SLC9A3R1 | H2AFV |
| GATAD1 | LOC101928291 |  | ATF7IP | LOC102723897 | SLC30A9 |
| CNOT11 | FAM199X |  | CPS1 | VDR | SUCO |
| UCHL5 | PPP2R5C |  | EPM2AIP1 | PTPRO | DYRK1A |
| MORF4L2 | FBXO4 |  | CLEC14A | TTLL6 | PNN |
| CDC73 | RAD1 |  | HOXA4 | CYTH2 | SERPING1 |
| ARIH2 | MAN1A2 |  | EPAS1 | MST1R | GREB1 |
| IPPK | RCBTB1 |  | GNG2 | CLDN7 | TMEM14A |
| GNB2 | ZFAS1 |  | FBRSL1 | IPO4 | NLGN4X |
| OAS1 | SNHG1 |  | JAM3 | PRSS3 | VPS13B |
| NRAS | TMCC1 |  | VPS13D | PARP3 | ZCCHC10 |
| ANKRD16 | MSANTD3 |  | PDE4DIP | ORMDL3 | LOC101060747 |
| PSMD12 | COMMD6 |  | FRG1CP | GCNT2 | FNBP4 |
| DRG2 | CSGALNACT2 |  | HTT | GOLM1 | SLIT2 |
| SRP19 | DCAF13 |  | ADAMTS9 | AGFG2 | TMEM165 |
| NUDT4P1 | KNOP1 |  | COMT | C6orf132 | LDHB |
| SLF2 | SEC63 |  | SART1 | FDCSP | ENTPD4 |
| SLC50A1 | NAP1L1 |  | NPAS3 | MAPK13 | AMOTL2 |
| ABHD17C | SNAPC1 |  | LOC654780 | KIAA1671 | HLA-E |
| QKI | ZNF180 |  | POLK | SDC1 | WLS |
| ARMC1 | HSPA13 |  | GJA4 | JAM2 | RAB21 |
| SETSIP | HAUS3 |  | POLR2A | HBG2 | HERPUD1 |
| RB1CC1 | TMEM154 |  | ZER1 | MIR210HG | SPRY1 |
| TXLNG | ACP6 |  | UBE4B | INSL5 | SERINC1 |
| UBE2D3 | GLMN |  | VPS11 | LGALS3 | CTSO |
| RIOK2 | LINC01315 |  | HEIH | CDKN1A | ZFPM2 |
| SETD2 | COMMD2 |  | ADD1 | POTEKP | PID1 |
| PI4KB | SLF1 |  | SVIL-AS1 | ICAM2 | RCBTB1 |
| APLP2 | ECT2 |  | NAB1 | RPH3AL | GALNT1 |
| UBE2G2 | RBM41 |  | DOCK6 | GSDMB | ASNSD1 |
| CHFR | TXN |  | PALMD | AHNAK | ALDH6A1 |
| HDGFRP3 | SGK494 |  | WDR61 | UGDH | TPM1 |
| CDC27 | PUS10 |  | INAFM1 | SPDEF | ALDH1A1 |
| EDEM3 | ANXA9 |  | NSUN5P2 | WDR13 | STAM |
| HYLS1 | CEP72 |  | TRAPPC2L | SPINK5 | CBFB |
| HLTF | KIZ |  | EHD1 | SLCO2A1 | COMMD3-BMI1 |
| CDCA5 | VRK1 |  | ZBTB46 | ST6GALNAC6 | KIAA0232 |
| SLC35B1 | ANGPT2 |  | ACTG1P4 | PDXK | RB1 |
| CDC20 | PACRGL |  | PLD2 | TRAF4 | RNF111 |
| PCNA | TMED3 |  | MMD | CORO2A | CREB3L2 |
| FAM103A1 | ZNF30 |  | AASS | KIAA1161 | C4B_2 |
| TROAP | ENOPH1 |  | ACKR1 | PEX11A | GOSR1 |
| SCOC | DSG4 |  | PLCD3 | DYRK2 | CD164 |
| PNPT1 | ADD3-AS1 |  | PECR | LMOD1 | EIF4G2 |
| MREG | TBXAS1 |  | RBSN | APPL2 | WTAP |
| TPX2 | ZNF485 |  | DENND2A | LARP1 | PIKFYVE |
| NUF2 | INHBA |  | DMD | MAP3K13 | UBA3 |
| HPRT1 | SLC19A2 |  | TRIM8 | BDKRB1 | HEXIM1 |
| RBM25 | ANKRD36 |  | NFRKB | EPS8L3 | WAPL |
| NAA20 | PRMT3 |  | NAT6 | TKFC | HTRA1 |
| CCNE2 | TRPM7 |  | LOC100507557 | STAP2 | CLK4 |
| CAPNS1 | FBXO45 |  | GSTM2 | MSRA | JAK1 |
| BMPR2 | RAB11FIP2 |  | LOC101928424 | SLC22A18 | TDP2 |
| EIF2S3 | SRP72 |  | ESD | APLNR | CXADR |
| ISCA2 | USP28 |  | RBFOX2 | CAMK2N1 | LAMB1 |
| PSMC2 | DNHD1 |  | PRPF6 | CLU | TOR1AIP1 |
| GRSF1 | PWP1 |  | RFX2 | NEDD4L | RPL31 |
| RAN | IMMP2L |  | SH3BP5 | ATP13A2 | RAB11A |
| DTNBP1 | GPR180 |  | IMPDH2 | UGP2 | DMXL1 |
| MIR21 | PRPF38B |  | PCBP4 | USH1C | SKAP2 |
| PLPP5 | FAM63B |  | CEP164 | HINT3 | KDM6A |
| MRPL35 | RNGTT |  | HDAC7 | PLPP1 | AUH |
| SZRD1 | GABPA |  | GHR | FAM114A1 | HP |
| PLPP2 | HMMR |  | SFPQ | ZBED1 | CAT |
| TJP1 | WDR35 |  | BEND5 | GSTM4 | DST |
| ARL2BP | MAP3K13 |  | D2HGDH | SCARA5 | SLC30A1 |
| GCH1 | AEBP2 |  | RPL36 | MEIS1 | KLF9 |
| CENPK | CCSAP |  | ST5 | ANGPTL1 | EMC2 |
| TMX2-CTNND1 | LOC145783 |  | AHNAK | PFN1 | ID4 |
| CASP3 | PSMA3 |  | MAMDC2 | CYP4F12 | AMMECR1 |
| PSMA1 | CDADC1 |  | ETFB | ENTPD3 | PLXDC2 |
| ZBTB1 | NRF1 |  | USP28 | IMPA2 | SEC14L1 |
| RAE1 | SNTB1 |  | ERCC1 | MPST | MED4 |
| ARL6IP1 | ANKRD46 |  | ANKDD1A | MT1H | RPS6KA2 |
| GTF2F1 | ABHD13 |  | IQSEC1 | MT1M | UPK3B |
| KLHDC10 | SPIN3 |  | BBS10 | PC | MAN1A1 |
| COQ4 | PHTF1 |  | FAM156B | LINC00294 | AHSA2 |
| PLAGL2 | DLGAP1-AS2 |  | MGLL | SERINC2 | ZFYVE16 |
| AQR | ARIH1 |  | DCAF4L2 | MAN2C1 | NOC3L |
| DIDO1 | STRN |  | PCGF3 | PDLIM5 | DEK |
| TSC22D2 | CHEK1 |  | ARHGEF1 | YIPF3 | PI4KAP1 |
| MIF4GD | LTV1 |  | IL34 | MIR5193 | HNRNPA0 |
| GANAB | BAG4 |  | MFNG | BAG1 | RAN |
| IDE | NUP205 |  | NEURL4 | ZNF341 | ATP9A |
| MAP7 | ZFX |  | RAB40B | SSPN | ZFAND1 |
| BCL2L12 | UTP6 |  | NATD1 | HIGD1A | BRD8 |
| TRPM7 | TSC22D1 |  | LOC100129550 | TMEM184A | LAMP1 |
| PSMB7 | MIR1304 |  | MYCBP2 | HEXIM1 | RPA1 |
| TMED10 | RFC4 |  | LPAR1 | AVPI1 | UNC50 |
| INIP | METTL14 |  | CPM | BDH1 | ARID5B |
| PTP4A3 | TDRD3 |  | MADD | MYO1A | NR2F2 |
| GPBP1L1 | SPATA7 |  | C6orf47 | CSTB | LY75-CD302 |
| ARFIP1 | MRPS35 |  | POMT1 | MOGAT2 | DCN |
| PSMA3 | KLHL15 |  | PDDC1 | EYA2 | VAMP7 |
| SPPL2A | GARNL3 |  | IGIP | ADAT3 | ADAM10 |
| ACTL6A | FAM168A |  | IL17RD | STK24 | LOC100507577 |
| SUV39H2 | FLJ41455 |  | LOC400499 | FBXW5 | TLK1 |
| TTC37 | PSTK |  | RASEF | ZNF575 | MS4A4A |
| PNKD | LOC107985971 |  | PDZD4 | TMEM220 | SLCO3A1 |
| GABPB1 | LOC101928747 |  | CASP9 | SEPW1 | SPTLC1 |
| RIDA | WDR89 |  | EIF3G | EGLN3 | PRKD3 |
| KRAS | SNORD50B |  | FLJ42627 | VIPR1 | GJA1 |
| ACOX1 | VCPKMT |  | AP5M1 | MT1G | NUP153 |
| CRTAP | NOC3L |  | RASL10B | CR2 | CDKN1B |
| CBX5 | BRIX1 |  | LRRN3 | SCN7A | CSTA |
| GPATCH4 | ENOX2 |  | DGAT2 | BAK1 | MAP3K4 |
| HIST1H2BE | PLA2G7 |  | AAMDC | BCAR3 | TSPAN8 |
| UTP4 | GOPC |  | PYROXD2 | BAIAP2L1 | PPM1B |
| PASK | BCOR |  | DCLK1 | SLC51A | KIAA0485 |
| MRPL38 | SUMO1 |  | HIPK3 | PDE4D | CX3CR1 |
| CEP76 | ZNF280C |  | ZBTB20 | UGT1A3 | CREBBP |
| RSBN1 | C18orf54 |  | SYNPO2 | IP6K2 | NACC2 |
| CBX3 | WDR5B |  | ZNF862 | ANK3 | AASDHPPT |
| NCAPD2 | MIR4657 |  | DET1 | RELL1 | TUBGCP3 |
| ZNF706 | NKAP |  | LYRM9 | IGFBP6 | CBX1 |
| SPG21 | MSANTD2 |  | LOC101930026 | NEDD9 | LZTFL1 |
| PCID2 | SMIM13 |  | NIPBL | PTP4A1 | MCFD2 |
| AK2 | FAM111B |  | HADH | SMIM22 | MIR4738 |
| POM121C | GSPT1 |  | GAS6 | SORT1 | BECN1 |
| DOPEY2 | LRPPRC |  | SPG21 | SLC6A8 | RBPMS |
| ZMPSTE24 | ZNF227 |  | CHST12 | TLN2 | GATM |
| TMF1 | RAD51AP1 |  | SSC5D | GPER1 | MICAL2 |
| PARP9 | E2F3 |  | SLC38A9 | MIR612 | DYNLT3 |
| PUM1 | PGM2 |  | PHRF1 | RNF19B | SGMS1 |
| PAICS | PARPBP |  | C4orf3 | EZR | SMARCE1 |
| G3BP1 | PSPH |  | CITED1 | PHGR1 | CLIC4 |
| BLOC1S2 | DNAJC21 |  | TPRG1L | VILL | SGCE |
| ANXA7 | MMD |  | PPM1A | CA7 | DYNC2LI1 |
| XRCC5 | BMT2 |  | ZNF25 | ZFYVE21 | CCNC |
| KIF20A | HDAC8 |  | GLI2 | IL2RG | MMADHC |
| TIAM1 | SPC25 |  | NDUFA11 | CAMSAP3 | GLT8D1 |
| SNRPB | BUB1B |  | MAP3K11 | SAR1B | TMEM123 |
| MIR7110 | RB1 |  | ERVH-6 | PRPF31 | SRSF11 |
| ZFYVE21 | PAPPA |  | FAM134A | IFI35 | TDG |
| MFSD14C | CLK1 |  | PCDH19 | GSTZ1 | TTC3P1 |
| BRCC3 | CFAP97 |  | NR3C2 | FBXL20 | KRT19 |
| WSB1 | CLK4 |  | TMEM184A | BTBD7 | LAMP2 |
| SENP3-EIF4A1 | ZNF184 |  | DAB2IP | ATOH1 | NMRK1 |
| TGIF2-C20orf24 | LOC389765 |  | SERINC3 | ACOT7 | LSM14A |
| UBR7 | REL |  | TSPAN4 | SLC16A5 | CRYZ |
| TMEM14B | CTDSPL2 |  | DPH5 | TNFSF10 | BEX4 |
| ABRACL | PHTF2 |  | SNU13 | P3H2 | DENR |
| RNF34 | MYB |  | LOC101927814 | SIAE | SPTBN1 |
| RABL3 | PSRC1 |  | SLC30A3 | SOWAHC | MAP4K4 |
| PANX1 | BUB1 |  | ACTA2 | ACBD3 | RANBP2 |
| DYNC1H1 | HJURP |  | SSBP2 | BEST2 | PLXNC1 |
| MFSD14A | SS18L2 |  | GSTM3 | TST | NAP1L1 |
| RECQL | SF3B1 |  | TTC28 | STARD10 | LOC101928625 |
| ZNF814 | HS3ST3B1 |  | MIIP | CROT | FOXO3 |
| SLC35F6 | PAIP1 |  | TP63 | PCK2 | SH3BGRL |
| NUDT21 | RAD18 |  | IL17B | EGFR | MBD2 |
| TOPBP1 | KIF16B |  | LMF1 | CGN | MKL2 |
| CDK2 | PPIL3 |  | CACNA1G | BDKRB2 | PCDHA1 |
| FAM60A | DPH5 |  | SUPT20H | TSPAN3 | PEG3 |
| UHRF1BP1 | HS2ST1 |  | DOK1 | TRIM15 | HNRNPM |
| METTL21A | SMARCAD1 |  | FAM160B2 | TBC1D1 | C11orf58 |
| MKI67 | TPT1-AS1 |  | DERL2 | SQRDL | SEC22B |
| LGALS8 | GPR183 |  | ZCCHC6 | B3GNT8 | KLF4 |
| PAIP1 | IMMP1L |  | SNTB2 | MAMDC2 | MRC1 |
| MCM9 | MAP4K3 |  | SH3BGRL2 | CASP7 | TOM1L1 |
| FBXW2 | PRIM1 |  | EVA1C | LOC100996506 | GOLGA8A |
| FBXO6 | RUFY3 |  | PPP1R16B | PLAUR | HIPK1 |
| RPEL1 | TGDS |  | PTGER3 | ZNF652 | CNIH1 |
| GJA9-MYCBP | FUT1 |  | HTR4 | AKR7A3 | KDM2A |
| OXSR1 | RBPJ |  | TDH | ATP2A3 | ARHGEF3 |
| FBXO9 | INPP4B |  | LOC101930112 | CCDC68 | CRY1 |
| GPRC5A | LOC105369477 |  | GGTA1P | TTC22 | UAP1 |
| SLC20A2 | ZC3H12B |  | POLR3H | CST3 | TRAM1 |
| HSPE1 | FBXO3 |  | PRDM15 | LGALS9 | HIBCH |
| FAM220A | POLR2K |  | SNHG19 | HAGLR | BIRC3 |
| UBQLN1 | UBA2 |  | COL7A1 | PKM | TM2D1 |
| UNC93B1 | LIPT1 |  | THRA | TP53I3 | CNOT2 |
| PDIA6 | MYCBP2 |  | CDC42EP5 | EMC1 | CCNI |
| SPIRE1 | CETN3 |  | HOXA3 | KCNMA1 | IER5 |
| SCAF11 | ORMDL1 |  | FBXW4 | SNRPA | HERC5 |
| LEMD2 | TPR |  | BLOC1S1 | MIR6787 | ARMT1 |
| FOSL2 | RPL31 |  | NRG1 | GIMAP1 | CUL5 |
| PPP2R1A | SNRPA1 |  | SHANK3 | PMM1 | CD163 |
| DNAJA1 | CBFB |  | FRMD4A | CHCHD10 | TNS3 |
| EPN2-IT1 | UBE2W |  | C8orf88 | ZGPAT | UBB |
| UPF1 | ZNF17 |  | LOC100996792 | KRT19 | SNURF |
| MTCH2 | KRIT1 |  | HGSNAT | MIR6872 | ENC1 |
| ZNF217 | LOC100134445 |  | FLT1 | HMOX1 | SOCS5 |
| KCTD15 | GPSM2 |  | DMAP1 | RAB11FIP4 | SEC23A |
| SULF1 | TIPIN |  | DDX59 | F2RL1 | SELENBP1 |
| GORAB | MRPS25 |  | TNS2 | FABP4 | EGFR |
| GGA2 | IGDCC4 |  | GOSR2 | HOXD1 | HSPA1B |
| MSH2 | DUS4L |  | MSRA | HOXB7 | CCNG1 |
| VPS29 | JADE1 |  | PRR29 | WDR18 | SNAP23 |
| PPP1R12A | BFSP1 |  | TSC1 | ALDH3A2 | ATG12 |
| SERPINE1 | HELLS |  | TRABD | TSPAN18 | FOXN3 |
| MSI2 | NCAPG |  | SPATA24 | HRCT1 | CTNNAL1 |
| SNX3 | RNMT |  | C2orf68 | SLC25A25-AS1 | CXCL6 |
| PROSER1 | COX20 |  | KLHDC8B | PCBP1-AS1 | PPP2CB |
| DHX9 | GTF2H5 |  | KMT2D | SGPP2 | AADAC |
| BAX | MDM1 |  | MIR6778 | POLR2E | PCM1 |
| TMED7-TICAM2 | ABHD10 |  | MEF2D | SULT1A2 | GBP2 |
| STT3A | MIR1204 |  | PLA2G4B | FBLN2 | ACTR6 |
| ATP6V0A2 | HNRNPLL |  | VPS4A | MFSD4B | CA12 |
| STAG2 | LINC00467 |  | LOC100996412 | SLC22A18AS | ANKRD36B |
| PCCB | RNASEH2B |  | CTDSPL | NAP1L2 | ALG5 |
| TIPRL | MIR4453 |  | PLEKHA8P1 | SEC14L1 | PDGFRA |
| SRRT | CYP27B1 |  | FGD5 | HHLA2 | SNAI2 |
| GATC | ARMCX5 |  | DDR2 | SLC51B | PKP2 |
| CMTM6 | FNDC3A |  | OBSCN | MYO1D | TGFBR3 |
| ABCB10 | TBC1D32 |  | TRAPPC12 | MT2A | ATG3 |
| UBAP2L | LOC105379499 |  | ZFYVE21 | CXCL12 | CTSC |
| PEX3 | STX6 |  | BACE1 | NPDC1 | FKBP1B |
| PRKRA | BRIP1 |  | FOLH1B | SPPL2A | KLHL2 |
| RPS6KB1 | TBC1D15 |  | DSTNP2 | TRIM10 | ERLIN2 |
| HMMR | METTL21A |  | SNORD65 | EFNB2 | EMC7 |
| MSANTD3 | LOC100506123 |  | ITGA1 | LMO3 | SMARCA5 |
| HSPA9 | TRIP11 |  | RAB6B | HBB | KLK11 |
| MTF2 | CEP68 |  | PECAM1 | NMRAL1P1 | YTHDF3 |
| RHBDD2 | ZC2HC1A |  | SRSF11 | AK1 | USP33 |
| TMEM106B | BID |  | LINC00184 | UNC13B | CD46 |
| SPTSSA | SFXN3 |  | LAMTOR4 | SMAD7 | RFK |
| SBK1 | CHD9 |  | DNAJB12 | SH3KBP1 | CCDC91 |
| TRMT1L | NUP107 |  | ABLIM1 | CNN1 | C12orf29 |
| DSTN | SLC12A2 |  | PARD3 | HN1 | GSTM3 |
| DAD1 | SCML1 |  | CEP85L | C16orf62 | AP5M1 |
| PTP4A2 | PPM1A |  | CEP162 | KRT20 | LAMTOR3 |
| NFKB2 | KIF18A |  | MIR548F5 | PLEKHA6 | PDIA6 |
| ATL3 | ESM1 |  | ANTXR2 | GAS2L1 | SYNCRIP |
| WSB2 | ADK |  | BDH2 | RNASE1 | SUCLG2 |
| YTHDF3 | ACBD6 |  | SPSB3 | CHP2 | PPP1R12A |
| TRIM59 | ZNF277 |  | LOC105373849 | NR1H4 | ESD |
| FAR1 | LRRC15 |  | ZNF654 | RABAC1 | LOC101930404 |
| RFC5 | ITGB3BP |  | PDGFD | P2RY2 | CD44 |
| XRCC4 | ZNF23 |  | TUBGCP2 | GABARAPL1 | RYBP |
| VRK2 | HIKESHI |  | UFSP2 | ACER3 | DERA |
| UBA6 | VPS50 |  | ZNF502 | STX12 | LEPROT |
| SETX | PPIL1 |  | DHX30 | NR4A1 | HPR |
| SMAD7 | THAP9-AS1 |  | LOC145783 | MT1F | TANK |
| GRPEL1 | ZNHIT3 |  | SMIM7 | SERPINB6 | UBQLN2 |
| CLIP1 | MTFMT |  | MYO1C | CRAT | NOTCH2NL |
| PDE7A | MSH6 |  | MRGPRF | SFRP2 | IGFBP7 |
| MYDGF | CKS1B |  | LOC100506548 | HSPB1 | EDNRA |
| SEL1L | LDLRAD3 |  | ALDH1B1 | SMCHD1 | RNF6 |
| TBK1 | MRE11A |  | NR1D1 | PSMF1 | DICER1 |
| LSM2 | KIF20B |  | TMTC1 | AHCYL2 | HOXD4 |
| CEP350 | CENPN |  | GIMAP1 | IL22RA1 | LOC100129518 |
| CERS2 | ZBTB21 |  | MED28 | XPNPEP1 | ZEB1 |
| ATG16L1 | CPSF3 |  | SIRT3 | FXYD3 | MYH10 |
| SULF2 | DPY19L3 |  | USE1 | DSG2 | IFRD1 |
| PMS2P3 | DSCC1 |  | GPRASP1 | KCNK6 | PDE1A |
| ATG12 | DPY19L4 |  | ZNF791 | S100A14 | DKK3 |
| MAPK13 | MIB1 |  | CACYBP | KLB | CFD |
| ELMOD2 | GTPBP4 |  | ZNF423 | AKR1C1 | MICU2 |
| UBE2D1 | HACE1 |  | CLECL1 | CTSE | VCAM1 |
| DENR | ACSL4 |  | CFH | ACSF2 | CD14 |
| GALK2 | MOSPD1 |  | LOC400927-CSNK1E | DENND2A | PDCD6 |
| HNRNPH1 | NPM1 |  | VSIG2 | CTSS | PSMC6 |
| NCEH1 | MTRR |  | DGCR2 | CPNE8 | TMEM100 |
| TRAPPC3 | NOL11 |  | IGHV4-31 | RPS6KA1 | BNIP3 |
| IMPACT | MTHFD2 |  | LINC00997 | ZDHHC3 | SRGN |
| ZNF124 | SS18L1 |  | AKR1C3 | IRF6 | FBXO21 |
| DUSP5 | DARS |  | LOC101060604 | SIPA1L2 | ABLIM1 |
| TMEM43 | C16orf87 |  | TPGS2 | FAM126B | GPNMB |
| PGM3 | EGFL6 |  | MOB3C | GADD45A | XIST |
| CDK16 | RCOR3 |  | DCHS1 | AKR1B10 | G0S2 |
| PEX1 | APBB2 |  | SNHG17 | WSB1 | OSBPL8 |
| LMNB1 | ASPH |  | NUMA1 | KIAA0513 | TRIM22 |
| LRRD1 | TUBE1 |  | TIE1 | PPP2CB | PEX2 |
| MTA3 | PIWIL1 |  | ZC3H6 | SLC35D1 | NAMPT |
| LOC101930655 | COMMD3-BMI1 |  | ID4 | HIST1H1C | S100A8 |
| ANKRD22 | LMBR1 |  | ASMTL | PLEKHA7 | PPFIBP1 |
| VPS72 | MTAP |  | FGFR1 | ASMTL | TMEM45A |
| TMX1 | NAF1 |  | SRSF8 | FOXA3 | PDGFRL |
| EIF3J | E2F6 |  | PCED1A | ABCC13 | LYZ |
| SUMO3 | RSL24D1 |  | RPL15 | SGMS2 | PSMB9 |
| COL10A1 | OGFOD1 |  | PTPN14 | TMCC3 | SPOCK2 |
| FCGR3B | TM4SF1 |  | RAB4B-EGLN2 | METTL7B | PLS3 |
| RAB11A | CANX |  | HSD17B1 | EMP3 | HLA-DQB1 |
| PLXNA3 | PDS5A |  | RPS6KA2 | FMO4 | BEX1 |
| COCH | PAPD5 |  | ATP7A | C1orf21 | CAPZA2 |
| TBC1D31 | RAD54B |  | NOP9 | ATP5S | FAM3C |
| APOBEC3B | C2orf76 |  | MED16 | EFNA5 | GCH1 |
| TOR1B | ZWILCH |  | NUDCD3 | GSTA1 | HLA-DPA1 |
| MMP11 | ZNF738 |  | SLC30A5 | BMP2 | PLK2 |
| DPM1 | BBX |  | SOD2 | MT1E | CD55 |
| MYCBP2 | AGPAT5 |  | TECPR1 | MGAT4A | PLOD2 |
| FBXO5 | THAP5 |  | GPALPP1 | TUBAL3 | HAT1 |
| MRRF | SNX16 |  | MYO9A | FLNA | CDH11 |
| C12orf29 | HECW2 |  | NSUN5P1 | MUC13 | LOC100272216 |
| SH3GLB1 | STRIP2 |  | CSRNP3 | CNNM4 | EMP1 |
| TMEM251 | TCEAL1 |  | RHOU | ELMO3 | PCK1 |
| HIST2H2BE | UBR5-AS1 |  | TINAGL1 | YIPF5 |  |
| CYP20A1 | GATAD2B |  | TAL1 | INO80C |  |
| ADA | LOC644656 |  | RALGDS | LAMB3 |  |
| SPAG5 | CENPU |  | EMCN | MBOAT1 |  |
| HIST1H2BC | TTC30B |  | ZNF793 | LTK |  |
| ARPC1A | BCCIP |  | RNASE4 | ABCG2 |  |
| SMAD5 | CNOT9 |  | ZNF575 | NAPRT |  |
| TGDS | PLA2R1 |  | HLA-E | SIDT1 |  |
| RER1 | ERLIN2 |  | RBM17 | PHLPP2 |  |
| MIB1 | CPOX |  | LOC101930123 | PNKP |  |
| PDCD10 | RBMXL1 |  | GPRC5B | LIMA1 |  |
| LIMS4 | CCT6A |  | ZMYM3 | PLPP3 |  |
| TBRG1 | DONSON |  | MTMR10 | MPDU1 |  |
| CRYZL1 | GSAP |  | RILPL2 | PAPSS2 |  |
| RRN3 | LOC101928625 |  | SYNGR1 | GPA33 |  |
| SNW1 | APITD1-CORT |  | PTPN18 | ANPEP |  |
| MCM8 | PFDN4 |  | PEX11G | MUC5B |  |
| ZNF200 | MCPH1 |  | FARS2 | PSEN1 |  |
| OSTF1 | DIO2 |  | PURA | LOC102724197 |  |
| MFSD14B | LOC100287896 |  | TNIP1 | CDA |  |
| TMED4 | TAOK1 |  | N4BP1 | ASS1 |  |
| CEP55 | RPGR |  | SLC44A1 | SLC25A20 |  |
| C7orf55-LUC7L2 | ALG10 |  | WBP1L | DPP10-AS1 |  |
| TMCC1 | GGCT |  | CLUHP3 | THBS1 |  |
| C4orf46 | TAF5 |  | LOC100131541 | LINC00483 |  |
| PRDX1 | NUDT21 |  | CD3EAP | FMO5 |  |
| RMDN1 | ZNF420 |  | IFI27L1 | ZNF91 |  |
| ELF4 | KIAA0907 |  | FXYD6 | MUC20 |  |
| EMC2 | UCHL5 |  | PLAGL1 | CNTN4 |  |
| WNK1 | CDC25C |  | PTOV1-AS2 | SLC16A9 |  |
| DGUOK | RGPD2 |  | LOC101930404 | FCGRT |  |
| WISP1 | LOC101060817 |  | DDX24 | GREM2 |  |
| C5orf24 | SNAP23 |  | LDLRAP1 | EIF4E3 |  |
| ARID1A | ZNF25 |  | ALKBH7 | MGAT4B |  |
| ILF2 | CKAP2 |  | EIF4B | PLAC8 |  |
| GGCX | ETV1 |  | PLCG1 | SLX1B-SULT1A4 |  |
| CXorf40B | NDUFAF4 |  | SLIT2 | MIB2 |  |
| PAFAH1B3 | LINS1 |  | LENG8 | SCNN1A |  |
| TMEM209 | SRSF6 |  | ZNF204P | FGFR2 |  |
| POLR1C | ZNF850 |  | IRS2 | KLF5 |  |
| TNPO1 | GPR65 |  | KLHL24 | LIPH |  |
| SERPINH1 | IKZF5 |  | KRBA1 | FAM132A |  |
| FAM83D | PKD2 |  | OPRPN | SLC26A2 |  |
| ERLEC1 | MSH2 |  | PLA2G6 | ASL |  |
| ABHD10 | CLDN1 |  | CNTNAP3B | SLIT2 |  |
| TIMM50 | TMEM41A |  | BNIP2 | STARD5 |  |
| BIRC5 | F2RL2 |  | HDGFRP2 | GGT2 |  |
| PSMD14 | FAN1 |  | CCBE1 | GPD1L |  |
| CRLS1 | MSANTD4 |  | PPM1H | FAM43A |  |
| HSPA5 | KIAA1958 |  | NFATC4 | DGAT1 |  |
| MIER3 | TTC14 |  | RAD50 | FAM46A |  |
| DNAJC9 | CD99P1 |  | MIR6513 | PPIC |  |
| TAOK1 | SLC25A15 |  | TMEM80 | CYP2C18 |  |
| PRR11 | CD46 |  | PHYKPL | SLC9A2 |  |
| USP46 | ARL6IP6 |  | INTS1 | CYP1B1 |  |
| IDH3A | TTC26 |  | TACC2 | AIFM2 |  |
| IQGAP1 | DHX40 |  | C2CD2 | CHRDL1 |  |
| TRIO | PDZD2 |  | EXOC4 | TRIM31 |  |
| KDM1B | EFCAB2 |  | ANXA11 | PKIB |  |
| LAGE3 | NUP35 |  | CYTH3 | MYZAP |  |
| PHF20L1 | TWISTNB |  | ME3 | CYCS |  |
| LOC374443 | ACP1 |  | SREK1IP1 | KRT8 |  |
| HUS1 | ARID4A |  | SLC25A16 | GCNT3 |  |
| CHMP1B | NMNAT3 |  | ANPEP | HOXB13 |  |
| GIGYF2 | PSIP1 |  | SIN3A | ME2 |  |
| NFKB1 | VASH2 |  | STAT2 | ST6GALNAC4 |  |
| CDC23 | TRIM2 |  | NDUFV1 | GPAT3 |  |
| ELP6 | C3AR1 |  | ANKHD1-EIF4EBP3 | WLS |  |
| MEN1 | ZDHHC17 |  | MYL6 | SELO |  |
| CASP8 | WISP1 |  | FGF7P3 | ABHD3 |  |
| BRPF3 | IGIP |  | CXXC1 | PPP1R12B |  |
| PLEKHA8 | AP3M2 |  | HHLA3 | HSD17B2 |  |
| METTL26 | DTL |  | PDE9A | GOT1 |  |
| SP100 | OLFML2B |  | PAK3 | SGK1 |  |
| NUMA1 | HSPH1 |  | INSR | ELOVL6 |  |
| PRMT1 | THAP6 |  | XPA | DES |  |
| ST3GAL1 | PTCD2 |  | TMEM200B | TMEM139 |  |
| FCGR1B | MOB1B |  | OARD1 | QSOX1 |  |
| CITED2 | BICD1 |  | ATP5I | UGT1A1 |  |
| WIPI1 | CD84 |  | B4GALT6 | KLF2 |  |
| PDIA3 | LIPA |  | TSR3 | HGD |  |
| FCGR1CP | GEN1 |  | THRSP | SLC17A4 |  |
| PPP1CB | PROX1 |  | FAM122A | UGT1A3 |  |
| LYRM2 | ALMS1-IT1 |  | ZNF148 | BRINP3 |  |
| XPOT | ABCB7 |  | SPX | TFCP2L1 |  |
| SMIM15 | RPGRIP1L |  | ICAM2 | FCGBP |  |
| KIF2C | POLQ |  | TUSC8 | STYK1 |  |
| KIF26B | SKAP1 |  | RBPMS2 | HTR4 |  |
| RGS19 | TAF2 |  | EEF1A1 | FXYD6 |  |
| HSD17B6 | EARS2 |  | CELF2 | GBA3 |  |
| GAS2L3 | SFXN4 |  | UTRN | GPAT2 |  |
| CTSB | LRP11 |  | MTURN | ST3GAL4 |  |
| KIF5B | SEPT7-AS1 |  | EXOSC10 | OR7E14P |  |
| MTDH | LOC105374366 |  | LOC101060443 | SMIM14 |  |
| ZNF227 | TAX1BP1 |  | TLE4 | MCOLN2 |  |
| C1S | MIS18A |  | CLNS1A | EPN3 |  |
| NSDHL | TEX30 |  | LARP6 | NR3C2 |  |
| ZBTB38 | MRS2 |  | TPCN1 | TMEM35A |  |
| ZXDC | DDX31 |  | IRS1 | C7 |  |
| EXOSC2 | SMIM11A |  | ANK2 | HPGD |  |
| TCTN3 | MBLAC2 |  | GALM | ABCA8 |  |
| WARS | NAMPT |  | GNAI1 | OAS1 |  |
| DAP | CAAP1 |  | PLSCR4 | CCL15-CCL14 |  |
| AGA | TCEB1 |  | STAT6 | FOXO3B |  |
| RFC2 | CDC7 |  | NCALD | TUBB2A |  |
| CALM3 | ZNF573 |  | TP53BP1 | LOC100652777 |  |
| COL11A1 | TMEM126B |  | PPP3CB | AIFM3 |  |
| GTF3C3 | RCL1 |  | TXNL1 | SEMA4G |  |
| BPGM | MBTD1 |  | GPM6B | EPB41L4A |  |
| TMEM2 | DNAAF2 |  | TMEM245 | C1orf115 |  |
| SEC22B | WHSC1L1 |  | PPP1R12B | PRR15 |  |
| UBA5 | XPO4 |  | ERCC6L2 | LOC339166 |  |
| DNMT1 | LINC00998 |  | CRYBG3 | MAOA |  |
| VPS35 | SPICE1 |  | NSUN6 | LRMP |  |
| TMEM57 | CCDC59 |  | NFIA | CCNYL1 |  |
| PSPC1 | TMPO |  | CTDSPL2 | CMBL |  |
| TADA1 | ZNF449 |  | GNB5 | NAT8B |  |
| ORC5 | LOC101930489 |  | RASGRF2 | SLC36A1 |  |
| HMOX1 | ZC3H13 |  | LOC101930115 | SMPDL3A |  |
| MTMR4 | YOD1 |  | FAM160B1 | SLC35A3 |  |
| STAT3 | LZTS3 |  | SDHAP2 | ZNF664-FAM101A |  |
| PIGH | ADNP |  | MSX1 | RSAD2 |  |
| PHAX | MRPL42 |  | ZNF662 | SGSM1 |  |
| APPBP2 | EEA1 |  | PFDN5 | C15orf52 |  |
| GMDS | TGFBR1 |  | DNAJB2 | CDHR2 |  |
| PAPOLG | DAB2 |  | EFEMP1 | KLF9 |  |
| MAPKAPK5-AS1 | PTAR1 |  | C1orf115 | NHSL1 |  |
| NAP1L4 | ITGA1 |  | MIR5193 | FAM107B |  |
| DCAF13 | SPTBN1 |  | FAM3D | NR5A2 |  |
| TMEM14A | FUNDC1 |  | CXCL12 | PBLD |  |
| PAPOLA | TMTC3 |  | AAK1 | LAMA3 |  |
| CORO2A | MIR664B |  | ECE1 | ID1 |  |
| SP1 | KIAA0895 |  | VPS13A | DSC2 |  |
| CHD4 | NEK3 |  | NPRL3 | FAM127A |  |
| COPG1 | TMEM261 |  | STOX2 | PYGB |  |
| CNPY2 | MALAT1 |  | ZNF462 | PAQR8 |  |
| HSPA13 | CELSR3 |  | ZNF667 | PAG1 |  |
| EIF5 | NME7 |  | SLC4A7 | IRF7 |  |
| CNST | XKRX |  | APLNR | FAM127B |  |
| PRKAG1 | MRPL36 |  | HRCT1 | CBLC |  |
| ZNF451 | NUFIP2 |  | GIMAP8 | NLRP2 |  |
| GALNT10 | SRSF1 |  | UBE2G2 | CWH43 |  |
| CACYBP | OR51E1 |  | LOC730268 | MT1HL1 |  |
| PNPLA8 | NAA16 |  | MXRA7 | EPB41L3 |  |
| AGFG1 | SUGT1 |  | KCTD17 | SAMD9 |  |
| CORO1C | HOOK3 |  | PDXK | CFD |  |
| GLUL | HMGB2 |  | GRAMD1C | PRKCB |  |
| UBE2V2 | TOP2A |  | CLK4 | RAB27A |  |
| LIG3 | TBCEL |  | ARID1B | TSPAN7 |  |
| OTUD5 | LOC101928054 |  | SCML2 | FA2H |  |
| NAA15 | N4BP2L2 |  | SNORA17B | ABHD12 |  |
| TAF1A | SLC16A4 |  | ZBED3 | EFNA2 |  |
| RNPEP | DLGAP5 |  | IL16 | SYNC |  |
| UBE2Z | PTPMT1 |  | ZBTB22 | ELP5 |  |
| HMOX2 | AK6 |  | DMTN | OASL |  |
| IPO9 | NDC80 |  | FAM200B | CCDC80 |  |
| ATP6V1B2 | NUTM2A-AS1 |  | C10orf10 | ANTXR2 |  |
| IPO5 | SMC4 |  | PEBP1 | FFAR4 |  |
| AIFM1 | TFDP1 |  | EFEMP2 | ATP2C2 |  |
| DEDD | NDC1 |  | LCAT | C4orf19 |  |
| POLR1B | RPF2 |  | FAT2 | PITPNM3 |  |
| SNRNP25 | THOC2 |  | CD99L2 | PTGIS |  |
| CXCL10 | ZXDB |  | WWP2 | GPRC5A |  |
| PITRM1 | SUZ12 |  | YPEL3 | MYOF |  |
| PPRC1 | USP45 |  | DHFR2 | EPHX2 |  |
| PLA2G12A | GEMIN8 |  | ITPK1 | MYLK |  |
| ARL4C | FAP |  | C21orf33 | SGK223 |  |
| SLC38A1 | LRRC40 |  | RNPC3 | RHOU |  |
| CHCHD1 | AKAP10 |  | ZNF581 | COL17A1 |  |
| MAPK9 | NHLRC3 |  | AKT3 | CDH19 |  |
| SKIL | DCUN1D5 |  | LOC102724093 | NKX2-3 |  |
| CDC7 | SMAD5 |  | KANK1 | ENDOD1 |  |
| EAF1 | KIF15 |  | OSBPL1A | MEP1A |  |
| SPATC1L | SGTB |  | RGS12 | MFSD4A |  |
| SLC35A2 | LOC102724851 |  | S1PR2 | PGM1 |  |
| PSAP | COL4A5 |  | OGN | LRRC19 |  |
| TRIM32 | TMEM5 |  | THSD7A | TPPP3 |  |
| C4orf33 | SIM2 |  | DNAJC4 | FKBP1B |  |
| SNORD89 | SNORD19B |  | SNX6 | CAMK1D |  |
| DAG1 | ZNF322 |  | PLBD2 | CYP2C9 |  |
| SMAD4 | TMEM268 |  | CAMLG | LOC100507472 |  |
| CEP85 | COPG2 |  | MEIS2 | LOC100505501 |  |
| ERI1 | FMNL2 |  | AGPAT2 | CAPN8 |  |
| BORA | EDNRB |  | KRBOX4 | BCAS1 |  |
| GNS | LOC101927391 |  | FAN1 | NFIC |  |
| EIF4G1 | TDRD6 |  | ITGA9 | CKMT1A |  |
| MIR6878 | UHRF1 |  | NLN | RHBDL2 |  |
| KMT2A | PBK |  | IDH1 | SRI |  |
| KIAA0907 | MYBL1 |  | LEPROT | RUNDC3B |  |
| CBX4 | KIF9 |  | LOC286161 | KIF16B |  |
| GOLGA3 | ARFGEF2 |  | TNS4 | IL18 |  |
| RBM26 | DDX10 |  | RCAN2 | LINC01133 |  |
| BLVRA | PPM1H |  | MED6 | LOC102725051 |  |
| LOC100507577 | ZNF711 |  | EML3 | GDA |  |
| PSMD11 | XPOT |  | PARP6 | PTGDR |  |
| CREBL2 | DHFR |  | PDGFRA | LINC00675 |  |
| SIK1 | CCT5 |  | VPS36 | COL6A2 |  |
| VPS37A | CREB5 |  | FBXO16 | TMEM176B |  |
| VPS13A | AKAP11 |  | SEMA6D | BCHE |  |
| RNF19B | SLC35E2B |  | NCKIPSD | LGALS4 |  |
| DNA2 | DRAM2 |  | ZNF540 | SAMD9L |  |
| TMTC3 | CNOT7 |  | WTIP | CD24 |  |
| TRRAP | PDZD8 |  | TMEM246 | DUSP5 |  |
| SLC7A5 | TSGA10 |  | PCDHGA1 | VWA5A |  |
| ANKRD12 | DBF4 |  | KCTD10 | GPM6A |  |
| APOOL | UQCC1 |  | EIF3F | KIAA1211 |  |
| ATP6V1D | GPAM |  | EBF3 | HPSE |  |
| HMGB3 | NEK5 |  | CCDC25 | FOS |  |
| HIST2H2AA4 | E2F7 |  | PLEKHH2 | GCG |  |
| MIR664B | LOC727820 |  | MITF | SYTL4 |  |
| EBAG9 | COL5A2 |  | KLHDC10 | KCNK1 |  |
| PTPN11 | CADPS |  | TMED10 | GLDN |  |
| ITFG1 | RAPGEF2 |  | OLFML2A | PLCL2 |  |
| DCBLD1 | YME1L1 |  | ERF | SLC28A2 |  |
| TRAPPC8 | TFAM |  | PPL | ADH1C |  |
| MIOS | PAICS |  | MGC70870 | SLC46A3 |  |
| MGEA5 | PROSER1 |  | EGFLAM | PLLP |  |
| CNOT6 | CCDC126 |  | LUC7L3 | CAPN9 |  |
| COMT | TMEM167A |  | SH3KBP1 | HSPA1B |  |
| RCOR3 | URB1 |  | ADGRL4 | SLC26A3 |  |
| ABL2 | TBC1D19 |  | HMGB1 | ISX |  |
| RNASEH2A | PTPMT1 |  | RSPO3 | CLCA1 |  |
| IQGAP3 | CASK |  | MON1B | IL1R2 |  |
| DLGAP5 | CEP120 |  | NAP1L1 | TLR3 |  |
| ARID2 | NEIL3 |  | GGA1 | GPX3 |  |
| RINT1 | RAD50 |  | MEF2C | GNE |  |
| C4orf3 | EZH2 |  | SLC35D2 | MYH11 |  |
| SRPRB | LLPH |  | PRIMA1 | GGT6 |  |
| TIMM17B | ATP2C1 |  | MIR4738 | C15orf48 |  |
| SHMT2 | SPATA13 |  | EMP1 | SPON1 |  |
| RBL2 | COPS2 |  | ZFYVE1 | MIR22 |  |
| MIF | CDK12 |  | AHI1 | A1CF |  |
| DHTKD1 | RIOK1 |  | MT1M | RBM6 |  |
| AMD1 | C4orf46 |  | EOGT | TCEA3 |  |
| ZNF654 | CASC7 |  | RERE | CASP5 |  |
| TET3 | ATP11C |  | ZNF506 | PGM5 |  |
| TTK | TGS1 |  | GID4 | PPID |  |
| CHMP2B | PLS3 |  | PCOLCE2 | SLC1A1 |  |
| POLR2D | RPL22L1 |  | DCN | FGF9 |  |
| KRCC1 | MED30 |  | ERG | PLCE1 |  |
| CTBP1 | C12orf4 |  | B3GALT6 | OLFM1 |  |
| PDSS1 | PSME4 |  | DYM | CLDN11 |  |
| XPO7 | LYPLAL1 |  | RETSAT | HSPA1L |  |
| CARS | ZC3HAV1L |  | PLA2G16 | C10orf99 |  |
| GTF2I | FBXO22 |  | ENG | CCL5 |  |
| ATR | GPR34 |  | UBQLN2 | TNIK |  |
| EHMT1 | AGA |  | MKLN1 | RNF186 |  |
| DDX55 | PTBP2 |  | NR2F2 | LGALS2 |  |
| MAVS | BRCA1 |  | LOC101930578 | CHFR |  |
| TFB2M | CEP112 |  | PMP22 | CXCL13 |  |
| NIF3L1 | CCDC50 |  | PTEN | HMGCS2 |  |
| TNFSF12-TNFSF13 | RUNX2 |  | DOCK4 | BHLHE41 |  |
| MSL2 | LUM |  | CTSG | PIGR |  |
| PDHX | GBP5 |  | IQCH-AS1 | PI3 |  |
| COL6A1 | SLC6A6 |  | CKMT2-AS1 | FABP1 |  |
| TSSC1 | STK3 |  | CXCL14 | LAMA1 |  |
| SLC30A6 | RRM2B |  | DTWD1 | FOSB |  |
| C2orf49 | TARBP1 |  | PCDH9 | MAOB |  |
| INTS12 | UBQLN2 |  | MGC27345 | LRRC66 |  |
| APTR | CDKN3 |  | ZCWPW1 | ATP8A1 |  |
| CASK | CEP290 |  | RP9P | PLA2G2A |  |
| TRIM33 | MYCN |  | TSNARE1 | PRKACB |  |
| SAP30 | REPS2 |  | PKP4 | HIST1H3F |  |
| G2E3 | LOC642852 |  | FUZ | PDK4 |  |
| RGS16 | C1orf109 |  | HIVEP2 | NXPE4 |  |
| CLDN3 | SPATA6L |  | SOX10 | SORBS2 |  |
| TRMT6 | SLC38A6 |  | TMX4 | SYNPO2 |  |
| LACTB2 | CCNA2 |  | AP3M1 | PCOLCE2 |  |
| POLR3F | WDR27 |  | ZNF302 | PROM2 |  |
| LOC101930416 | MIR6756 |  | LRRC37A2 | SCG2 |  |
| TRIM38 | SRPK1 |  | POLR3G | WFDC2 |  |
| GTPBP2 | SESTD1 |  | COL21A1 | ADRA2A |  |
| SDC1 | ART3 |  | TTC17 | VLDLR |  |
| TMPO | SCOC |  | RIOK3 | PRSS12 |  |
| PKMYT1 | MYLIP |  | MAP2K7 | ANO5 |  |
| NUS1 | WHSC1 |  | HNRNPDL | MLPH |  |
| RFC3 | TTF2 |  | LOC101930583 | PDE5A |  |
| DIS3 | ADAM22 |  | PIK3C3 | LOC101060835 |  |
| STK35 | SENP6 |  | RASSF6 | MUC1 |  |
| GTF2H2C | RPP40 |  | OFD1 | PCK1 |  |
| GTF2H3 | FAM122B |  | UBE2D3 | ABI3BP |  |
| CSE1L | SNORA21 |  | SSU72 | NELL2 |  |
| C6orf1 | FCGR2A |  | MINOS1 | MYL9 |  |
| PSMA2 | KCMF1 |  | SEMA5A | OSBPL1A |  |
| S100A11 | MRPL17 |  | UCK1 | VIP |  |
| CLIC4 | RHOBTB1 |  | ABCD4 | MB |  |
| RACGAP1 | IRF2BP2 |  | LYRM1 | GNAI1 |  |
| EMC4 | CALU |  | SNHG8 | FAM134B |  |
| SBDSP1 | WRN |  | DPP8 | SEPP1 |  |
| SGO2 | PTCD3 |  | POM121L9P | LIFR |  |
| ARID4B | MRPL15 |  | NSFL1C | NAT2 |  |
| DHX36 | CTPS1 |  | PFKFB3 | SULT1B1 |  |
| FAM126B | CENPI |  | PRELP | FGFBP1 |  |
| HIVEP1 | SACS |  | IGSF9B | MAB21L2 |  |
| TMPPE | MMP7 |  | IFI27L2 | TMEM56 |  |
| DBF4 | RSF1 |  | EWSAT1 | ABCB4 |  |
| RALA | ORC1 |  | ZNF266 | THRB |  |
| SMYD3 | TCEA1 |  | MST1 | DUOX2 |  |
| CBFB | ENY2 |  | SAP30L | C3orf70 |  |
| CA5BP1 | EXTL2 |  | GAS8 | PDE3A |  |
| TOMM22 | POLB |  | KIF1B | GHR |  |
| LDLR | MLLT3 |  | TBC1D22A | CYBRD1 |  |
| EIF1AX | SHISA2 |  | HYMAI | L1TD1 |  |
| F11R | TTK |  | TEN1-CDK3 | ARL14 |  |
| DNAJC7 | FZD3 |  | NIPAL3 | HDAC9 |  |
| MLLT10 | VPS13A |  | LOC100289333 | ITLN1 |  |
| TSPAN31 | MED1 |  | NOSIP | BCL2L15 |  |
| PACSIN2 | AMMECR1 |  | CAMTA2 | SRPX |  |
| MAP3K13 | INTS2 |  | ASXL1 | HYAL1 |  |
| COMMD5 | BIVM |  | FOLR2 | CRIP1 |  |
| PRDM10 | SRD5A1 |  | KCNC4 | HSPA2 |  |
| PEX11B | TXLNG |  | GRAPL | ST6GALNAC1 |  |
| BCAP29 | RFXAP |  | ZNF106 | PP7080 |  |
| MYOF | GDAP1 |  | PCM1 | RBM24 |  |
| PHF19 | GPN3 |  | FCGBP | ABCB1 |  |
| PSRC1 | GKAP1 |  | ZC3H7B | SLC13A2 |  |
| AMMECR1 | LINC01420 |  | TSGA10 | RARRES1 |  |
| HIPK1 | MPP6 |  | ADIRF | MS4A1 |  |
| TMED5 | KIF4A |  | MYL6B | IQGAP2 |  |
| PPP1CA | SGMS1 |  | UBE2I | ZSCAN18 |  |
| NTMT1 | ZNF286B |  | CLDN8 | CHGB |  |
| LSM14B | USPL1 |  | CYP2U1 | SI |  |
| CYLD | FBXL2 |  | TMEM67 | PLEKHA2 |  |
| GTF2B | ING3 |  | CDC42EP2 | ENPP3 |  |
| TFRC | NDUFAF2 |  | GALT | PRAC1 |  |
| SIPA1L2 | ZNF532 |  | SGSM3 | IGHA2 |  |
| C6orf136 | CEP55 |  | RIMKLB | NPY1R |  |
| MFAP2 | TMPRSS3 |  | RPARP-AS1 | FOXP2 |  |
| LOC101928879 | MGA |  | PRDM11 | REG4 |  |
| TRIP11 | TOMM70 |  | ABCB4 | ZC3H12C |  |
| ATM | PNPT1 |  | GIT1 | TFF1 |  |
| MIR3656 | CENPL |  | HIP1R | HEPACAM2 |  |
| HSP90AA1 | EIF3C |  | CEP57 | CLIC6 |  |
| RSRC1 | PRKD3 |  | NOS1AP | CNTN3 |  |
| FAM208A | PLAA |  | HEMK1 | DEFB1 |  |
| CYCS | MNS1 |  | P2RY12 | AGR3 |  |
| MAD1L1 | MTFR1 |  | LINC00328 | DMBT1 |  |
| POLR2J4 | PRLR |  | ETV5 | AXDND1 |  |
| SNX14 | TMEM55A |  | TMEM129 | ALDH1A1 |  |
| UBE2K | NME1 |  | HSBP1 | GSTT1 |  |
| PAFAH1B1 | DKK3 |  | PPFIBP1 | UGT2B17 |  |
| ASF1B | PCDH18 |  | CASC4 | LOC101926951 |  |
| PIGO | FZD6 |  | LOC646214 | SPINK4 |  |
| PACRGL | GRHL1 |  | FAM20C | UGT2A3 |  |
| DDOST | CKS2 |  | MYO15B |  |  |
| PIGB | ZNF254 |  | SLC29A4 |  |  |
| FNDC1 | PLEKHA5 |  | ZNF561 |  |  |
| STARD4 | ZBTB33 |  | BTBD6 |  |  |
| ZNF195 | CEP170P1 |  | HOXA10 |  |  |
| LACTB | DDX52 |  | KIAA1217 |  |  |
| PLP2 | ADAM32 |  | ABCA6 |  |  |
| FPR3 | COL12A1 |  | WNK2 |  |  |
| TMEM259 | BICC1 |  | FAM161B |  |  |
| SF3B4 | ATP6V1C1 |  | TBXA2R |  |  |
| CISD2 | RAB30 |  | MOB3B |  |  |
| ACBD3 | TASP1 |  | SMU1 |  |  |
| LAMTOR3 | RPL32P3 |  | LETMD1 |  |  |
| NVL | ZMYM6 |  | PNKD |  |  |
| NUP155 | ERVMER34-1 |  | DAAM2 |  |  |
| MORN2 | ZNF81 |  | IGBP1 |  |  |
| AKAP1 | CEMIP |  | EIF1 |  |  |
| HECTD1 | ALG11 |  | MMP24-AS1 |  |  |
| CLN3 | SLC22A3 |  | SMAD5 |  |  |
| GINS2 | DNAJB9 |  | TADA2B |  |  |
| GTSE1 | POLR3F |  | VAPB |  |  |
| TNRC6A | UPF3B |  | FBXO9 |  |  |
| SF3B6 | ZNF550 |  | ARHGEF5 |  |  |
| ADAM19 | LIN7A |  | TACC1 |  |  |
| FAM91A1 | FAM60A |  | AP1S2 |  |  |
| GSKIP | ITPR2 |  | ZNF274 |  |  |
| COPE | PAK1IP1 |  | SLC25A29 |  |  |
| SERP1 | MIR15A |  | FOXO3 |  |  |
| RHOB | CCDC167 |  | CHD8 |  |  |
| NADK2 | MRPS28 |  | ZFYVE27 |  |  |
| ALG2 | BBS9 |  | ARRDC2 |  |  |
| PSMD5 | ZNF818P |  | CABLES1 |  |  |
| THUMPD3-AS1 | ASAP1 |  | SLC35E1 |  |  |
| CHMP5 | LOC101927027 |  | HERC2P7 |  |  |
| FAM45A | ZNF239 |  | NES |  |  |
| C1orf112 | SKP2 |  | ELF5 |  |  |
| BARD1 | STK31 |  | C3orf18 |  |  |
| BUB1 | ARHGAP24 |  | VPS13C |  |  |
| RDX | PPIP5K2 |  | POMT2 |  |  |
| CTSS | TOMM20 |  | HNRNPA0 |  |  |
| SHCBP1 | COL1A1 |  | RARRES2 |  |  |
| DNPH1 | GPR137B |  | RNF170 |  |  |
| MRPS28 | GTF3A |  | ZNF629 |  |  |
| TBC1D24 | DNAJC9 |  | LAMA4 |  |  |
| RIPK2 | FAM13B |  | TRIM66 |  |  |
| PRDM2 | NARS2 |  | NANOG |  |  |
| TLCD1 | KIF13A |  | PDLIM3 |  |  |
| CD9 | TTI1 |  | FAM63A |  |  |
| CDC45 | ZAK |  | ALDH9A1 |  |  |
| NECAP1 | TUBGCP3 |  | STX7 |  |  |
| ORMDL1 | CHCHD7 |  | FAM71A |  |  |
| AURKB | IFT81 |  | TBC1D5 |  |  |
| TNFAIP3 | APOOL |  | DNASE1L3 |  |  |
| PDZD11 | SLC13A3 |  | LZTR1 |  |  |
| ESYT2 | SOX9 |  | SMARCD3 |  |  |
| NSMAF | CEP41 |  | NAP1L5 |  |  |
| NCAPG | ETS2 |  | PIGV |  |  |
| TAF9B | MID1 |  | STAC2 |  |  |
| NCAPD3 | SEC24A |  | GLDN |  |  |
| SEPSECS-AS1 | ZNF597 |  | SNCG |  |  |
| TBC1D5 | CCT2 |  | ZNF564 |  |  |
| KIAA0196 | ANP32E |  | CHID1 |  |  |
| ZBTB21 | KBTBD6 |  | ABHD5 |  |  |
| LSM12 | LOC441155 |  | ZNF548 |  |  |
| SMG9 | POMP |  | CLIP4 |  |  |
| DIP2B | PAPD4 |  | FLNC |  |  |
| PRMT2 | KLF3-AS1 |  | NMT2 |  |  |
| CHST11 | KNL1 |  | KLHL29 |  |  |
| GOLT1B | ADAMTS12 |  | PCGF5 |  |  |
| ZCCHC14 | GLS |  | APOL2 |  |  |
| RNF13 | COG6 |  | PPP2R5E |  |  |
| LOC101929356 | DCBLD2 |  | LOC105369662 |  |  |
| CBLL1 | KBTBD7 |  | ARIH2 |  |  |
| SRSF7 | ITGA2 |  | KCTD7 |  |  |
| SNORA29 | UNC5CL |  | LOC105372526 |  |  |
| MED10 | MZT1 |  | TRIM9 |  |  |
| NDC80 | EIF2S3 |  | LNPEP |  |  |
| TMEM267 | ZNF318 |  | GRK5 |  |  |
| PDCD6 | DNMT3B |  | NAV2 |  |  |
| LOC100190986 | SLC39A6 |  | TGFBR2 |  |  |
| DNAJC21 | LINC00525 |  | SSPN |  |  |
| TMBIM6 | OSGEPL1 |  | LRP3 |  |  |
| KIAA1147 | BORA |  | HLA-F-AS1 |  |  |
| PLOD2 | ENAH |  | PCDH18 |  |  |
| SLC35D1 | TGFBI |  | MPST |  |  |
| ANKRD28 | UPF3A |  | SAA2-SAA4 |  |  |
| UGCG | TRIM23 |  | TCEAL7 |  |  |
| USO1 | TSC22D1-AS1 |  | C14orf28 |  |  |
| ZBTB8A | EIF3F |  | PCYOX1 |  |  |
| PTPRF | SLC25A30 |  | DHRS3 |  |  |
| EEF1E1 | COG3 |  | ARHGAP10 |  |  |
| EIF2S2 | EPHX4 |  | LRIG2 |  |  |
| TBC1D3P1-DHX40P1 | TMTC4 |  | HIPK2 |  |  |
| SH3BGRL3 | PCID2 |  | MARVELD1 |  |  |
| C2orf42 | LPCAT2 |  | INPP1 |  |  |
| CEP152 | C11orf74 |  | RNF169 |  |  |
| TP53I11 | THNSL1 |  | COL4A3BP |  |  |
| TMEM33 | SNORD87 |  | TRPC1 |  |  |
| TUBG1 | ID2B |  | C19orf12 |  |  |
| CEBPG | CCNB1 |  | LOC102725451 |  |  |
| KIF22 | ADAM12 |  | BDKRB2 |  |  |
| APITD1-CORT | ATP7A |  | ARHGAP6 |  |  |
| STAG3L2 | GLCE |  | AGO1 |  |  |
| NQO1 | ZNF623 |  | LOC105373878 |  |  |
| RGS1 | CHEK2 |  | ST3GAL3 |  |  |
| C8orf33 | MIR1292 |  | CDK5RAP2 |  |  |
| SMIM13 | RHOQ |  | CCDC180 |  |  |
| LSM8 | LOC102724951 |  | JAG1 |  |  |
| CDV3 | HSPD1 |  | OIP5-AS1 |  |  |
| LLGL2 | MELK |  | MIR6789 |  |  |
| TTC17 | SDHAF3 |  | SH3RF2 |  |  |
| BROX | PHACTR3 |  | MIR1282 |  |  |
| TMEM8A | ARHGAP18 |  | PIDD1 |  |  |
| ZC3H15 | DUSP14 |  | PPM1L |  |  |
| MPLKIP | GCLM |  | NICN1 |  |  |
| SLC35F5 | SNRPD1 |  | APP |  |  |
| GALE | NIT2 |  | RELL1 |  |  |
| BBX | SMARCA1 |  | MIR6125 |  |  |
| EXOC4 | NAPB |  | FAM172A |  |  |
| CLCN3 | MAP4K4 |  | ZNF826P |  |  |
| MRPL15 | LOC101927826 |  | CNTNAP3B |  |  |
| CD44 | ANOS1 |  | CCDC93 |  |  |
| CKAP4 | RBL1 |  | EPB41L2 |  |  |
| SGK494 | NOM1 |  | BOK |  |  |
| SPATA5L1 | PARP8 |  | GGT7 |  |  |
| COA6 | CBX2 |  | CNOT6L |  |  |
| TCF3 | CLHC1 |  | COBL |  |  |
| ANKH | SLC1A4 |  | TLE2 |  |  |
| SNORA72 | CDK6 |  | ZFP90 |  |  |
| NAGA | KPNA2 |  | PRPF38A |  |  |
| SCFD1 | ARID5B |  | C11orf58 |  |  |
| RIF1 | NNT-AS1 |  | RAD1 |  |  |
| CYB5B | FTCDNL1 |  | RCBTB2 |  |  |
| TRPS1 | TEX9 |  | OSER1-AS1 |  |  |
| RABGGTB | PRDX4 |  | ANKRD20A11P |  |  |
| APPL2 | SORBS2 |  | MAP3K4 |  |  |
| GMPR2 | LMNB1 |  | CCDC84 |  |  |
| CEP70 | UTP18 |  | ADHFE1 |  |  |
| GPSM2 | EDNRA |  | AHSA2 |  |  |
| MED19 | MIR146A |  | ADGRL3 |  |  |
| MDM4 | LOC441666 |  | ANKRD6 |  |  |
| TMEM168 | WWP1 |  | ZNF775 |  |  |
| PLD3 | DGKH |  | TFDP2 |  |  |
| KIF14 | LOC285147 |  | LOC202025 |  |  |
| RTCA | ANAPC5 |  | TMEM256 |  |  |
| PSMA3-AS1 | SLC8A1 |  | FLT3LG |  |  |
| CLP1 | FBXL17 |  | RPL35A |  |  |
| CHMP4B | HSD17B12 |  | KCNK3 |  |  |
| TMED7-TICAM2 | FNBP4 |  | PML |  |  |
| NCK1-AS1 | GZF1 |  | ALPK1 |  |  |
| PPP1R10 | GNL3 |  | AVIL |  |  |
| GET4 | TNFAIP6 |  | CDKN2C |  |  |
| ARHGAP32 | LEF1 |  | TFPI |  |  |
| PPM1G | PXYLP1 |  | RNF10 |  |  |
| ANKFY1 | CHURC1 |  | FAM126A |  |  |
| TSTA3 | SSB |  | EBF2 |  |  |
| KPNB1 | KIF11 |  | NTAN1 |  |  |
| NOM1 | MET |  | ANAPC16 |  |  |
| RSRP1 | PRMT6 |  | BRAP |  |  |
| EIF2AK2 | MED14 |  | HNRNPR |  |  |
| CENPM | ARRDC3 |  | HGF |  |  |
| MYO1B | LINC00888 |  | ENKD1 |  |  |
| NDFIP2 | CDCA7 |  | PCBP2 |  |  |
| MED14 | FOXP4-AS1 |  | TLCD2 |  |  |
| GNB5 | CHORDC1 |  | GBP2 |  |  |
| TEAD4 | FAM185A |  | LZTS2 |  |  |
| TCFL5 | STIL |  | FAM46A |  |  |
| ZNF367 | ANKRD50 |  | STXBP1 |  |  |
| MIR612 | YAE1D1 |  | TFEB |  |  |
| SNAPC3 | H2AFJ |  | LOC101060275 |  |  |
| SNX5 | DOCK11 |  | NEIL2 |  |  |
| HIST1H2BD | CBFA2T2 |  | SGSH |  |  |
| LRRFIP1 | CRYZ |  | CTTNBP2 |  |  |
| HMGB1 | ZBED8 |  | KBTBD8 |  |  |
| APTX | NCAPG2 |  | KIAA1652 |  |  |
| S100P | HP1BP3 |  | MECOM |  |  |
| PTTG1 | SNRPF |  | MAP1A |  |  |
| TRAM2 | FOXQ1 |  | ALDH3A2 |  |  |
| METAP2 | DHX9 |  | TMUB2 |  |  |
| MIR6125 | PSMC2 |  | GAB2 |  |  |
| HIBADH | HERC2P7 |  | SHC2 |  |  |
| WDR61 | ZNF112 |  | ERMAP |  |  |
| SYAP1 | ZWINT |  | H6PD |  |  |
| PTRH1 | TCTEX1D2 |  | ATG12 |  |  |
| HMGN4 | S100A9 |  | CARD6 |  |  |
| RCOR1 | ZKSCAN8 |  | ACVR2A |  |  |
| SLC39A6 | MBTPS2 |  | ARHGAP4 |  |  |
| ATG7 | DPCD |  | MKNK2 |  |  |
| NFYA | MRPL13 |  | INPP5K |  |  |
| QTRT2 | RACGAP1 |  | CLCN7 |  |  |
| CHAC2 | LARP4 |  | TRAK1 |  |  |
| PCNP | SP2-AS1 |  | PPP2R2A |  |  |
| RPL17-C18orf32 | ASCL2 |  | TVP23C-CDRT4 |  |  |
| PRIM1 | RWDD2A |  | SLC22A17 |  |  |
| MAP2K4 | WDR3 |  | MPRIP |  |  |
| UBE3C | BICD2 |  | MIR100HG |  |  |
| RPP38 | TIGD1 |  | ADGRF5 |  |  |
| MRPS30 | PANX1 |  | MIGA1 |  |  |
| MRPL19 | ZXDA |  | DDB2 |  |  |
| OAS3 | NUFIP1 |  | FMOD |  |  |
| NUP50 | TRAPPC2B |  | LINC00312 |  |  |
| CALD1 | PTPN22 |  | LOC105378577 |  |  |
| TAF15 | OBSCN |  | SPATA2L |  |  |
| KIF4A | CKMT2-AS1 |  | COL14A1 |  |  |
| MAD2L1BP | CCAR2 |  | RMDN1 |  |  |
| GCLC | TOMM6 |  | TBC1D17 |  |  |
| HGSNAT | RORA |  | TNFRSF14 |  |  |
| DNAJC14 | EDEM3 |  | ARHGEF37 |  |  |
| SLC35A3 | NASP |  | PDK2 |  |  |
| PRMT5 | ANAPC7 |  | SDK2 |  |  |
| CHD9 | ZNF137P |  | ARHGAP31 |  |  |
| RAD51AP1 | SLFN5 |  | LLGL1 |  |  |
| HJURP | SQLE |  | C20orf194 |  |  |
| MFSD8 | RPP30 |  | LOC100506990 |  |  |
| GDAP2 | BLACAT1 |  | WLS |  |  |
| UMPS | UTP14A |  | SF3A3 |  |  |
| HIST1H2BJ | KIRREL |  | CALCOCO2 |  |  |
| PGM2L1 | EEF1AKMT1 |  | IST1 |  |  |
| KIF3A | DACH1 |  | ARL15 |  |  |
| STIM2 | TRERF1 |  | SEC62 |  |  |
| ALOX5AP | PRKDC |  | ZBTB44 |  |  |
| BID | CDC6 |  | RAB33B |  |  |
| SDS | MTERF3 |  | GOLGA2P10 |  |  |
| PUS7 | THBS2 |  | RHOBTB3 |  |  |
| TXN | AMZ2 |  | PDPR |  |  |
| TUBB | PCDH17 |  | ZNF324 |  |  |
| IPO11-LRRC70 | MAGOHB |  | LUC7L |  |  |
| CABLES2 | MMP12 |  | C18orf54 |  |  |
| TMEM87B | LOC285902 |  | MAFF |  |  |
| KCTD3 | AIG1 |  | AKR7A2 |  |  |
| PCDH17 | PPP1R3D |  | GPR153 |  |  |
| NCOA3 | SLC9B2 |  | PC |  |  |
| COPS5 | FUT8 |  | HSPG2 |  |  |
| DAAM1 | TCFL5 |  | DIS3L |  |  |
| TCF19 | ASNS |  | DOK4 |  |  |
| CCDC117 | ZNF44 |  | ZNF346 |  |  |
| TRIQK | ASF1A |  | SMARCA2 |  |  |
| AGO3 | TNKS2 |  | NT5C3B |  |  |
| DLD | TBC1D8 |  | CACNA2D1 |  |  |
| MAD2L2 | SERPINI1 |  | SNORD77 |  |  |
| ROCK1 | CAMTA1 |  | PTPRB |  |  |
| STAU2 | LACC1 |  | MCRIP1 |  |  |
| GNL3L | LUC7L3 |  | LINC00938 |  |  |
| SMC1A | SUPT3H |  | MYEOV |  |  |
| FIGNL1 | ERO1B |  | UBOX5 |  |  |
| UBXN7 | GLS2 |  | TMEM42 |  |  |
| MIR6734 | CCDC34 |  | ZNF563 |  |  |
| PAQR4 | ADGRL4 |  | FAM13C |  |  |
| RRP15 | WDYHV1 |  | MESP1 |  |  |
| PANK3 | LOC401317 |  | SLC38A10 |  |  |
| SUB1 | BLM |  | PACS2 |  |  |
| PDK3 | PDLIM5 |  | TNKS |  |  |
| KMT2C | TRIP13 |  | ECSIT |  |  |
| NLK | CCNE2 |  | CREBBP |  |  |
| SLC38A6 | SNHG8 |  | MAATS1 |  |  |
| ZNF567 | ARL5B |  | GSPT1 |  |  |
| CD24 | FAR1 |  | AHDC1 |  |  |
| PNRC2 | SOX4 |  | MPPED2 |  |  |
| CDK13 | TSPAN5 |  | USP47 |  |  |
| NPRL3 | MCC |  | IGFBP4 |  |  |
| TMOD3 | NUSAP1 |  | TAZ |  |  |
| NDUFS1 | COL11A1 |  | CHMP4A |  |  |
| RCC1L | CDK8 |  | DR1 |  |  |
| EMC8 | OIP5 |  | ACVR2B |  |  |
| C1QBP | POLR3G |  | FZD7 |  |  |
| FAM208B | MGC32805 |  | EGF |  |  |
| ICAM1 | SLC7A11 |  | RPS29 |  |  |
| DR1 | RRN3P1 |  | ARHGEF6 |  |  |
| HS2ST1 | PAN3 |  | TSC22D4 |  |  |
| NPL | CDKL5 |  | HMBOX1 |  |  |
| MIS18A | HACD3 |  | LOC100289230 |  |  |
| RNF114 | FRMD5 |  | ACO1 |  |  |
| MED13 | BBOX1-AS1 |  | EPHB3 |  |  |
| PHLDA2 | LOC283357 |  | TIMM22 |  |  |
| C12orf49 | EIF3H |  | AVPR1A |  |  |
| ITSN2 | DZANK1 |  | REV3L |  |  |
| KIAA0391 | LAMC1 |  | GOLGB1 |  |  |
| USP48 | E2F5 |  | SCAF11 |  |  |
| GLRX | TDRKH |  | C7 |  |  |
| MRPS7 | TTN-AS1 |  | PIP |  |  |
| CHML | CD3EAP |  | YIPF6 |  |  |
| HSPA1B | ANXA1 |  | SIL1 |  |  |
| MPZL1 | DYNC2H1 |  | GPSM1 |  |  |
| ALAS1 | RNF170 |  | PI15 |  |  |
| NBN | MORC4 |  | TMEM248 |  |  |
| FGD6 | AZGP1 |  | ACD |  |  |
| KNSTRN | ASCC3 |  | C5orf56 |  |  |
| EIF2AK3 | LCP2 |  | GATSL2 |  |  |
| SNRNP27 | CCSER2 |  | MAN2C1 |  |  |
| FECH | SAMHD1 |  | RIN3 |  |  |
| DNAJA2 | GPR160 |  | CCDC152 |  |  |
| DDX58 | BCAS4 |  | MICU3 |  |  |
| H2AFX | TMEM231 |  | NALCN |  |  |
| RNF138 | QKI |  | MT1X |  |  |
| DNAJC16 | EIF4B |  | TNRC6C |  |  |
| COMP | MSANTD3-TMEFF1 |  | KRR1 |  |  |
| TMEM260 | COA1 |  | ZNF677 |  |  |
| GEN1 | CRISPLD2 |  | PTGIS |  |  |
| GPD2 | CKLF |  | ZBTB7A |  |  |
| SORD | NEDD4 |  | SRGAP2C |  |  |
| RMI2 | MIR1182 |  | TIMM10B |  |  |
| FAM49B | KIF2C |  | TMEM140 |  |  |
| ADAM10 | LRRC37A2 |  | C22orf29 |  |  |
| STAG1 | KLHL7 |  | TEK |  |  |
| TMEM126B | CTLA4 |  | FAM69B |  |  |
| BACH1 | AURKA |  | MZT2B |  |  |
| CCDC47 | AHCY |  | FAM160A2 |  |  |
| PSIP1 | SLC22A15 |  | FAM47E-STBD1 |  |  |
| DSN1 | GPX2 |  | EMID1 |  |  |
| PHKA1 | GINS2 |  | SYT8 |  |  |
| FASTKD3 | SUV39H2 |  | FYN |  |  |
| RBM47 | CREBRF |  | OGG1 |  |  |
| CDK8 | MAK16 |  | NUB1 |  |  |
| GTF2H4 | USP1 |  | TRA2A |  |  |
| YBX1 | RPS21 |  | RALGAPA2 |  |  |
| PTS | CTSH |  | PXN |  |  |
| MEAF6 | SLC19A3 |  | ODF3B |  |  |
| SUPT16H | FAM217B |  | FER |  |  |
| SEC24D | LOC100506548 |  | TSPAN7 |  |  |
| SPOPL | NOP16 |  | CXCL2 |  |  |
| PSMA5 | POLR1D |  | ANKRD40 |  |  |
| CHUK | BACE2 |  | FBLN1 |  |  |
| SOX4 | COL1A2 |  | ZNF362 |  |  |
| SLC29A1 | TTBK2 |  | MUCL1 |  |  |
| MTUS1 | PROCR |  | TCF4 |  |  |
| MKLN1 | MYO5A |  | TENM2 |  |  |
| ERLIN2 | TNFSF15 |  | RGMA |  |  |
| TTF2 | BCAT1 |  | LOC101927811 |  |  |
| VDAC3 | MKI67 |  | ZSCAN26 |  |  |
| LAMP2 | TMEM68 |  | NPHP3-ACAD11 |  |  |
| WDR5 | TYW3 |  | FSTL1 |  |  |
| SMU1 | AUNIP |  | SLC25A6 |  |  |
| TPD52L1 | SESN3 |  | ERICH1 |  |  |
| NCF2 | SNORD3D |  | EBF4 |  |  |
| TACO1 | ZFHX3 |  | FAM193B |  |  |
| ANKHD1-EIF4EBP3 | KLHL5 |  | PINX1 |  |  |
| PRPS2 | TFEC |  | ATP6V1D |  |  |
| GINS1 | LOC101930583 |  | TSC22D1 |  |  |
| PSMA7 | ABCC4 |  | FUBP1 |  |  |
| CEP170P1 | ANKRD29 |  | NR1H3 |  |  |
| IRF6 | CRNDE |  | DHRS4L2 |  |  |
| CENPE | HSPA12A |  | PLEKHH1 |  |  |
| IFI30 | SRPRB |  | EIF3J-AS1 |  |  |
| LDHA | GPX8 |  | PAIP2B |  |  |
| RANBP1 | IFIT5 |  | TMOD2 |  |  |
| C1QTNF3-AMACR | DDIAS |  | PLGLB1 |  |  |
| KMT2B | TRAF5 |  | PIK3C2B |  |  |
| GGPS1 | KBTBD8 |  | CPE |  |  |
| TLE1 | SEMA3A |  | THRB |  |  |
| IDH2 | ALCAM |  | PRNP |  |  |
| PHLPP2 | CRLS1 |  | GGA2 |  |  |
| UPK3BL | NHS |  | NAV1 |  |  |
| LOC101927705 | FANCI |  | GEMIN4 |  |  |
| SLC2A4RG | ITGB1BP1 |  | TWIST1 |  |  |
| DMXL2 | ZNF311 |  | SIK3 |  |  |
| STK17B | PLAU |  | RXRA |  |  |
| GOT2 | PHF19 |  | SP4 |  |  |
| MRPL17 | TMEM63A |  | ARHGEF10L |  |  |
| ABHD3 | TRIM4 |  | ZSWIM7 |  |  |
| PTGR1 | SLC9A7 |  | PLEKHO2 |  |  |
| UNK | KIAA0101 |  | DDAH2 |  |  |
| HSPA14 | IFI44 |  | FERMT2 |  |  |
| GNPDA2 | PDP1 |  | PHKB |  |  |
| HIST1H2BK | FAM72A |  | CAPN6 |  |  |
| CCT6A | ERAP1 |  | TNFRSF1B |  |  |
| MRPL34 | TIFA |  | PHLDA1 |  |  |
| CUX1 | ADGRL3 |  | SMAD3 |  |  |
| CDH1 | DYX1C1 |  | PDCD11 |  |  |
| APOO | CCNB1IP1 |  | C2orf27A |  |  |
| NORAD | FRG1CP |  | HIBCH |  |  |
| CXorf38 | MINCR |  | PELI2 |  |  |
| CKLF-CMTM1 | TFPI |  | PLLP |  |  |
| TOM1L1 | ARL4C |  | ZNF529 |  |  |
| NIPA2 | LOC728554 |  | NSMCE1 |  |  |
| AAGAB | OLA1 |  | GULP1 |  |  |
| WDR12 | B3GLCT |  | SNORD114-3 |  |  |
| NIPA1 | LAMB1 |  | EPM2A |  |  |
| DHCR7 | SPATA17 |  | METAP1D |  |  |
| VIMP | MCM4 |  | NFE2L1 |  |  |
| RHOQ | LOC100506098 |  | GPAT3 |  |  |
| SREK1IP1 | PRDM5 |  | MTMR3 |  |  |
| PBX3 | RBMS1 |  | MEIS3P1 |  |  |
| SLC25A28 | PNISR |  | BMS1P20 |  |  |
| MAP4K4 | NBN |  | TMEM178A |  |  |
| SEC22C | TNFRSF11B |  | GABRP |  |  |
| TUSC2 | LYPD6 |  | LOC728323 |  |  |
| FAM122B | PSAT1 |  | TTC12 |  |  |
| GEMIN6 | PRPS1 |  | SLC25A23 |  |  |
| DCPS | ELAVL2 |  | SVBP |  |  |
| PATZ1 | LINC00920 |  | MIR7113 |  |  |
| KLF6 | PHKA1 |  | PLEKHG4 |  |  |
| CSPP1 | QPCT |  | KCTD2 |  |  |
| MAP2K1 | CCDC77 |  | SLC25A37 |  |  |
| NAA35 | SNHG3 |  | PRR34-AS1 |  |  |
| SLC31A1 | FMR1 |  | LMO2 |  |  |
| CHD2 | LOC101929787 |  | FCGRT |  |  |
| CAB39 | GPX7 |  | TACR1 |  |  |
| DDX21 | GTF2H3 |  | FGFR1OP |  |  |
| CHORDC1 | HMCN1 |  | NFATC1 |  |  |
| MCCC2 | CTHRC1 |  | PGLS |  |  |
| FN1 | B4GALT6 |  | MIR675 |  |  |
| SERBP1 | MRPS23 |  | MALAT1 |  |  |
| HELLS | LOC101929036 |  | SH3D19 |  |  |
| NAPG | TGIF2 |  | PHACTR2 |  |  |
| RAD23A | CCDC88A |  | PACSIN2 |  |  |
| MBNL1 | SPATA5 |  | INS-IGF2 |  |  |
| PSME3 | LAMP3 |  | LTB4R |  |  |
| ABCA1 | COL6A3 |  | TP73-AS1 |  |  |
| AASDH | SPRED1 |  | SCARNA15 |  |  |
| PLAA | NSUN7 |  | PLEKHA2 |  |  |
| SCAMP1 | KCNH8 |  | TMED1 |  |  |
| C6orf132 | VSNL1 |  | SIX5 |  |  |
| POLR2C | PPP2R2C |  | TMEM170B |  |  |
| ENTPD1 | ACSL6 |  | CCDC85B |  |  |
| CDK12 | COL4A1 |  | CES4A |  |  |
| ASPN | DNAJC3-AS1 |  | TRNP1 |  |  |
| KDM6A | GINS1 |  | ARL10 |  |  |
| CDK2AP2 | GLCCI1 |  | KLHDC4 |  |  |
| PIGM | HOOK1 |  | KANK2 |  |  |
| PRDM1 | GEMIN5 |  | BAG1 |  |  |
| NECTIN4 | CACNA1D |  | GPLD1 |  |  |
| RFC4 | SHANK2 |  | ENOSF1 |  |  |
| NCK1 | SALL4 |  | SAP18 |  |  |
| ABHD2 | CBX4 |  | MIRLET7BHG |  |  |
| ZMYM2 | NANP |  | RIC1 |  |  |
| KIFC2 | CTSC |  | EHF |  |  |
| VEZF1 | FAM84B |  | USP53 |  |  |
| ORC2 | MCM6 |  | UBE2D4 |  |  |
| PQBP1 | PHLDA1 |  | PLEKHG3 |  |  |
| MRPL30 | ZNF697 |  | TBX5 |  |  |
| GFM2 | SYK |  | SLC26A3 |  |  |
| BRAF | TMEM170B |  | AIF1L |  |  |
| ARL13B | IL7R |  | ARSD |  |  |
| MAT2A | IGFBP7 |  | PHC1 |  |  |
| RHNO1 | PREP |  | SNX22 |  |  |
| IPO4 | SULF1 |  | SPRY1 |  |  |
| MS4A7 | ATP10D |  | DVL2 |  |  |
| ISCA1 | MNDA |  | LOC105379499 |  |  |
| GAPDH | MIR1908 |  | PEAK1 |  |  |
| SEH1L | PTCH1 |  | ZNF839 |  |  |
| EIF4G3 | MS4A6A |  | ALDH6A1 |  |  |
| FOXM1 | SMKR1 |  | ZHX3 |  |  |
| TCEB1 | GPC4 |  | CREB5 |  |  |
| MCM2 | GRK3 |  | NDUFAF4 |  |  |
| BAG2 | FREM2 |  | AKAP13 |  |  |
| DNAJA3 | SLC25A32 |  | SALL2 |  |  |
| PDP1 | LOC101930578 |  | SGCG |  |  |
| BTF3L4 | PWAR6 |  | LRRC56 |  |  |
| NMD3 | ADAMTS6 |  | BCR |  |  |
| KNOP1 | ABI2 |  | PEMT |  |  |
| KIAA0141 | TPD52L1 |  | CHL1 |  |  |
| HIKESHI | SLC16A10 |  | RPS28 |  |  |
| CPT2 | LACTB2 |  | TAOK1 |  |  |
| PPP1R14B | FAM120C |  | PRICKLE2 |  |  |
| PAFAH2 | E2F8 |  | SYTL4 |  |  |
| KDM4A | PTK2 |  | METTL9 |  |  |
| SMG8 | CEP76 |  | KRT17 |  |  |
| LPIN2 | SYBU |  | MGEA5 |  |  |
| NT5C3A | MIR452 |  | MPZL1 |  |  |
| EXOC2 | FAM92A1 |  | SPRY2 |  |  |
| RPL17-C18orf32 | PDK3 |  | MTERF2 |  |  |
| OSBPL3 | MCUB |  | C11orf95 |  |  |
| ZFAS1 | RGS1 |  | MRVI1 |  |  |
| CTSZ | ZNF83 |  | ITGB1BP1 |  |  |
| VASP | PTTG1 |  | LY75-CD302 |  |  |
| SVIP | ZNF280B |  | TMEM101 |  |  |
| NUDCD2 | TTC39C |  | ERMARD |  |  |
| CPNE3 | PTPN13 |  | KCNAB1 |  |  |
| CHKA | AGT |  | IQSEC2 |  |  |
| SDHB | MEGF6 |  | DDX58 |  |  |
| CHAMP1 | ANKRD36B |  | SMIM11A |  |  |
| CRNKL1 | CXCL3 |  | TBC1D2B |  |  |
| ZNF436 | STK38L |  | GAK |  |  |
| MRPS15 | RAB23 |  | LOC101930349 |  |  |
| MARCKSL1 | GSTA4 |  | COL16A1 |  |  |
| FBN1 | FTX |  | NUDT18 |  |  |
| DGKA | NID1 |  | CLIP3 |  |  |
| ZNF304 | CHI3L1 |  | LINC00926 |  |  |
| MCFD2 | LAPTM4B |  | JMY |  |  |
| ALKBH5 | FAM216A |  | SLC24A1 |  |  |
| MOSPD1 | MMP3 |  | PCCA |  |  |
| OCRL | AKT3 |  | DKK3 |  |  |
| ZCCHC6 | JADE3 |  | OGFRL1 |  |  |
| COQ7 | ZNF559 |  | MELTF |  |  |
| ARHGEF2 | LOC101928195 |  | ZNF729 |  |  |
| STX16 | FAT4 |  | BCL2 |  |  |
| GOLGA2 | VMP1 |  | MIR29C |  |  |
| MFSD6 | ADAMTS2 |  | LOC728392 |  |  |
| PERP | LIPG |  | SLC22A3 |  |  |
| MFHAS1 | SNHG16 |  | PTPN21 |  |  |
| PDE8A | DSE |  | ACTR3 |  |  |
| MPC2 | RRM2 |  | PCMTD1 |  |  |
| TSEN2 | KCNJ3 |  | IPP |  |  |
| MYO19 | SLCO5A1 |  | C15orf38-AP3S2 |  |  |
| AKIRIN2 | MPEG1 |  | ICA1L |  |  |
| SLAIN2 | SULT1C2 |  | ADCY3 |  |  |
| ESPL1 | IRS1 |  | MAPK8IP3 |  |  |
| MRPS18A | LRRC6 |  | FAM213A |  |  |
| RAP2A | GUCY1B3 |  | S1PR1 |  |  |
| SSRP1 | LOC100129518 |  | KAZN |  |  |
| CUL4B | LOC101927196 |  | SLC9A9 |  |  |
| NAMPT | ARFGEF3 |  | RBM33 |  |  |
| SNX1 | TDO2 |  | IKZF5 |  |  |
| ANKRD13C | TBC1D30 |  | LZTS1 |  |  |
| SRGAP2 | ADGRF5 |  | DIAPH2 |  |  |
| ARFGAP3 | CENPW |  | OBSL1 |  |  |
| STARD3NL | GIMAP2 |  | GIMAP7 |  |  |
| TIPIN | CCDC113 |  | BAZ2B |  |  |
| MRPS18B | RGMB |  | OR2A9P |  |  |
| METTL13 | NRP1 |  | TUBG2 |  |  |
| EFCAB11 | FAM201A |  | TDRP |  |  |
| DLG3 | ATP6V1C2 |  | LINC00667 |  |  |
| ENY2 | CXCL8 |  | AMIGO2 |  |  |
| RALGAPB | LINC01004 |  | NEK8 |  |  |
| CTTN | CTTNBP2 |  | ZNF493 |  |  |
| BAG5 | PODXL |  | ATXN7L1 |  |  |
| PRMT9 | HECTD2 |  | MED13L |  |  |
| SLC30A1 | ROBO1 |  | ZNF625-ZNF20 |  |  |
| TCEB3 | BMP7 |  | PCSK5 |  |  |
| NUP160 | LAMA4 |  | MAP2 |  |  |
| OLMALINC | RGCC |  | PRR5 |  |  |
| KIF18B | STXBP6 |  | CEBPA |  |  |
| CDK10 | ZNF506 |  | ANKRD11 |  |  |
| GALNT1 | HS6ST2 |  | SETBP1 |  |  |
| PPIL1 | SH3BP4 |  | GATAD2B |  |  |
| MIR6758 | F3 |  | SESN3 |  |  |
| MIR4738 | FAM83D |  | RAB24 |  |  |
| RFX5 | PM20D2 |  | TIAL1 |  |  |
| AP1M2 | FLT1 |  | C3orf38 |  |  |
| RUSC1-AS1 | LGR5 |  | PTGES3L-AARSD1 |  |  |
| ITGB2 | JPH1 |  | APH1B |  |  |
| IMPA1 | LOC730101 |  | ACSL1 |  |  |
| FAP | LOC100289090 |  | PLIN5 |  |  |
| SAMD9 | KLHL29 |  | GSTA4 |  |  |
| AGK | ARG2 |  | TMEM64 |  |  |
| HNRNPLL | CFTR |  | ANKRD10 |  |  |
| PSMG1 | ANKRD44 |  | PPARG |  |  |
| TGM2 | SFTA2 |  | ZNF573 |  |  |
| BLOC1S3 | DNAJC6 |  | CAV2 |  |  |
| AHCTF1 | IFITM1 |  | DAAM1 |  |  |
| MINPP1 | SCARNA2 |  | NMB |  |  |
| UFM1 | MSX1 |  | RNF145 |  |  |
| CSTF2 | PCDHB14 |  | CTNNB1 |  |  |
| ZNF43 | S100A6 |  | SLC25A12 |  |  |
| OGT | NABP1 |  | LAMA2 |  |  |
| THBS1 | COCH |  | IDS |  |  |
| GTPBP4 | C2CD4A |  | CYBRD1 |  |  |
| SLC38A2 | PAN3-AS1 |  | MAGI2 |  |  |
| YES1 | UTS2 |  | LTBP2 |  |  |
| CHEK1 | TPX2 |  | PDK3 |  |  |
| ZFAND5 | CYP4F3 |  | LOC100506718 |  |  |
| PAPD4 | LDHB |  | BAIAP2-AS1 |  |  |
| KIF15 | SNX10 |  | COQ4 |  |  |
| AIP | SAP30 |  | NEDD8-MDP1 |  |  |
| PLPP4 | LSAMP |  | ZDHHC14 |  |  |
| CDC123 | NR2F1 |  | AGPS |  |  |
| DNAJA4 | TRPS1 |  | PIK3IP1 |  |  |
| CXCL9 | C11orf70 |  | PLA2G4C |  |  |
| COMMD2 | PLAG1 |  | KHSRP |  |  |
| GID8 | SLC35F6 |  | INTS3 |  |  |
| LOC728554 | GATM |  | PRRX2 |  |  |
| NARS2 | SCD |  | MACROD2 |  |  |
| CNNM4 | STC2 |  | GUCY1A3 |  |  |
| SNX24 | FARP1 |  | PRKCDBP |  |  |
| GATC | CCNB2 |  | GALK2 |  |  |
| PKM | SCML4 |  | MIR4680 |  |  |
| ADAM9 | SORD |  | ZNF790-AS1 |  |  |
| CLEC7A | LDLRAD4 |  | PDZRN3 |  |  |
| P4HA1 | OAS2 |  | ZNF446 |  |  |
| TRAPPC1 | ARHGAP29 |  | GPAT4 |  |  |
| MIR1292 | PTPRO |  | NR2F1 |  |  |
| FAM199X | GZMB |  | HOXD10 |  |  |
| IQCB1 | MMP1 |  | MAF |  |  |
| PPIL3 | LYAR |  | COL18A1 |  |  |
| SGPP1 | HSPA4L |  | ABI3BP |  |  |
| LOC101927027 | LOC729680 |  | GPER1 |  |  |
| KDM5A | TIMP3 |  | THOC5 |  |  |
| MTERF3 | QSER1 |  | RTN2 |  |  |
| SUPT4H1 | PRC1 |  | FGF2 |  |  |
| OIP5 | ZNF529 |  | PDE1C |  |  |
| FNIP1 | ZKSCAN7 |  | ALG13 |  |  |
| NNT | COBLL1 |  | FMC1 |  |  |
| LCLAT1 | SLC6A20 |  | GIMAP1-GIMAP5 |  |  |
| SPDL1 | MS4A7 |  | LOC729966 |  |  |
| MAZ | DUSP4 |  | CKB |  |  |
| KDELC1 | HLTF |  | LOXL4 |  |  |
| LNPK | F5 |  | DPP7 |  |  |
| GSE1 | N4BP2L1 |  | DLK1 |  |  |
| TPR | CSRP2 |  | RTN1 |  |  |
| WRB | FRMD6 |  | ORAI3 |  |  |
| EPB41L4B | CKMT2 |  | CRIPT |  |  |
| SPATA5 | COL10A1 |  | LINC01140 |  |  |
| TMEM181 | COL8A1 |  | STAB1 |  |  |
| ZRANB1 | GGH |  | CDK5RAP3 |  |  |
| ESF1 | GNB4 |  | GFOD1 |  |  |
| TMEM19 | TRIB3 |  | ZNF503 |  |  |
| DHX8 | MME |  | TNN |  |  |
| SNRNP40 | EPSTI1 |  | ITGBL1 |  |  |
| LOC100294033 | CH25H |  | KIAA1462 |  |  |
| SH3RF1 | LPAR6 |  | LAMA3 |  |  |
| GREM1 | KHDRBS3 |  | ECHDC3 |  |  |
| IMMP1L | LRRC34 |  | NLRP1 |  |  |
| SLC44A2 | CDH3 |  | FAT3 |  |  |
| SOCS5 | KIF20A |  | TRIP10 |  |  |
| ACOT7 | CXCL11 |  | RASL12 |  |  |
| MRPS6 | PABPC1L |  | SGCB |  |  |
| FYB | TDGF1P3 |  | HEXIM1 |  |  |
| CD86 | MYC |  | CHKB-CPT1B |  |  |
| RUSC1 | PRNP |  | ARHGAP24 |  |  |
| STK38L | LPL |  | IFFO1 |  |  |
| UGGT1 | WT1 |  | LOC100652930 |  |  |
| KNL1 | HILPDA |  | PLPP3 |  |  |
| B4GALT4 | ANTXR1 |  | BCAM |  |  |
| MICB | LOC653602 |  | TMEM237 |  |  |
| SOCS6 | DGAT2 |  | CYB5D1 |  |  |
| RAI14 | IL20RA |  | DLGAP4 |  |  |
| MED28 | MACROD2 |  | POLN |  |  |
| GOLM1 | GRB10 |  | PTHLH |  |  |
| HES6 | PLCB1 |  | TOM1L2 |  |  |
| MYBL2 | BLVRA |  | CALML3 |  |  |
| SFSWAP | MFAP3L |  | PODXL |  |  |
| AP1S2 | DCDC2 |  | ARHGEF10 |  |  |
| PYCRL | ESRRG |  | CPXM2 |  |  |
| THRIL | DUXAP10 |  | AP4S1 |  |  |
| E2F8 | HSP90AB1 |  | LOC100996741 |  |  |
| TXNRD1 | CXCR4 |  | FLJ45482 |  |  |
| NAA40 | MCTP1 |  | CTNNBIP1 |  |  |
| KIAA1468 | PITX2 |  | MKNK1 |  |  |
| USP31 | TMEM97 |  | LPP |  |  |
| RABEP1 | IFITM2 |  | KIF13A |  |  |
| LRRC8E | CPA3 |  | PGRMC2 |  |  |
| BRWD1 | LINC01296 |  | CAMK1 |  |  |
| TOX4 | FABP6 |  | LOC100505549 |  |  |
| MYO5A | MITF |  | LOC102725051 |  |  |
| WDR77 | COL5A1 |  | ETS2 |  |  |
| MFSD11 | MCOLN3 |  | FBXW12 |  |  |
| RHOC | LARP6 |  | TMEM44-AS1 |  |  |
| ATF7IP | SNORD50B |  | RBMS1 |  |  |
| C6orf89 | PROSER2 |  | DSEL |  |  |
| INSIG1 | MXRA5 |  | MID1 |  |  |
| RAB31 | IL33 |  | KIAA1671 |  |  |
| SRXN1 | TMEM150C |  | SLC48A1 |  |  |
| ZNF664 | PDGFRA |  | ANXA1 |  |  |
| FAM149B1 | B3GALNT1 |  | MDN1 |  |  |
| SPAG9 | AMIGO2 |  | TMEM261 |  |  |
| MIR7113 | PTPRC |  | RCN1 |  |  |
| CAMK2G | ACKR3 |  | LOC102724562 |  |  |
| CCSAP | SHC3 |  | TRIM29 |  |  |
| LMNB2 | AXIN2 |  | LOC100505874 |  |  |
| CYR61 | GLT8D2 |  | SMAD9 |  |  |
| SRSF4 | CCL20 |  | ITPR1 |  |  |
| IGHM | RNF183 |  | FAM117A |  |  |
| AMZ2P1 | ALDH1L2 |  | OPTN |  |  |
| RUNX1-IT1 | CPVL |  | B3GALT5 |  |  |
| TBC1D8B | ANKRD22 |  | SPARCL1 |  |  |
| C9orf16 | TXLNGY |  | MIR205 |  |  |
| STK24 | FRY |  | RNASEH2C |  |  |
| ZADH2 | SLC35D3 |  | PLD1 |  |  |
| ATG4A | CXCL6 |  | LONRF1 |  |  |
| KLF7 | ST6GAL1 |  | S100A13 |  |  |
| GOSR1 | ZNRF3 |  | MYL9 |  |  |
| MIAT | RASSF10 |  | CPLX1 |  |  |
| ATP2C1 | ZMAT1 |  | NUAK1 |  |  |
| SEC63 | MS4A4A |  | KLHL3 |  |  |
| AKAP13 | FIBIN |  | CARD8 |  |  |
| AKAP8L | LOC103091866 |  | HSPA12A |  |  |
| DDX20 | PLA2G16 |  | VIT |  |  |
| MAGOHB | GAD1 |  | CSNK2A2 |  |  |
| TTC13 | PDGFC |  | PDPN |  |  |
| TDRKH | CXCL10 |  | CCDC80 |  |  |
| TMEM185A | IL17RD |  | IL4R |  |  |
| VAV3 | SLC28A3 |  | PPM1M |  |  |
| ANKRD39 | POSTN |  | CRISPLD1 |  |  |
| FLOT1 | SRPX2 |  | RGS5 |  |  |
| HNRNPL | PLCB4 |  | FZD3 |  |  |
| TERF2 | CEL |  | TMEM47 |  |  |
| GLIPR2 | PIPOX |  | PRDM2 |  |  |
| RAD51 | IL7 |  | NEURL3 |  |  |
| ALG6 | SLC4A11 |  | PTCH1 |  |  |
| TMC6 | S100P |  | KCNJ2 |  |  |
| RMND1 | EPHB1 |  | ACTG2 |  |  |
| RNF41 | DPEP1 |  | FBLN7 |  |  |
| SPATS2L | MYEF2 |  | NPHP3 |  |  |
| CAD | CXCL1 |  | MIR1182 |  |  |
| CIAPIN1 | HMGA2 |  | S100A4 |  |  |
| GPATCH2 | LOC101930404 |  | TEFM |  |  |
| LRRC42 | PCDHB9 |  | TYRO3 |  |  |
| STK17A | KRT23 |  | SATB1 |  |  |
| KLHL7 | UTY |  | RIN2 |  |  |
| AP1S1 | IGF2BP3 |  | PLA2G12A |  |  |
| NUP205 | SLCO1B3 |  | NOB1 |  |  |
| CLDN7 | RNF217 |  | PGGHG |  |  |
| RCBTB1 | WDR72 |  | SLCO3A1 |  |  |
| CCT5 | DEFA6 |  | TMEM55A |  |  |
| LMNA | TMEM71 |  | DDX17 |  |  |
| LAP3 | PKIA |  | ZNF207 |  |  |
| CEP63 | MYRIP |  | CYP27A1 |  |  |
| MCM3 | EMB |  | TSPOAP1 |  |  |
| B4GALT3 | BNIP3 |  | COL27A1 |  |  |
| DNAJB14 | REG3A |  | PTPRG |  |  |
| AKT1 | DSC3 |  | FAM135A |  |  |
| SKA3 | S100A8 |  | STAG3L2 |  |  |
| DNAJC19 | TSPAN2 |  | SPON1 |  |  |
| FGFR3 | ZIC2 |  | HK2 |  |  |
| PTGFRN | CXCL14 |  | ATP9A |  |  |
| PBX1 | UCA1 |  | APBB1IP |  |  |
| MAP4K2 | APCDD1 |  | TRIM68 |  |  |
| CDK4 | DEFA5 |  | CPAMD8 |  |  |
| CTPS2 | WIF1 |  | NRXN3 |  |  |
| TRUB1 | PTGS2 |  | FAM210B |  |  |
| CDCA3 | KIAA0226L |  | APBA1 |  |  |
| RAB3IP | TCN1 |  | RGS2 |  |  |
| OSMR | MAP7D2 |  | BHLHE41 |  |  |
| TRMT11 | HOXA10-HOXA9 |  | EFCAB14 |  |  |
| IARS | SAMD5 |  | FRZB |  |  |
| RUVBL1 | TACSTD2 |  | LINC01315 |  |  |
| TDP1 | KRT6B |  | HRASLS5 |  |  |
| C8orf76 | CYP4X1 |  | ADRA2A |  |  |
| DIMT1 | UBD |  | TBX3 |  |  |
| ZNF652 | FAM3B |  | PKIG |  |  |
| AIMP1 | FZD10 |  | SEMA6A |  |  |
| LURAP1L | SPINK1 |  | KIF13B |  |  |
| MUM1 | LY6G6F |  | LAMC2 |  |  |
| MCM10 | CLDN2 |  | TGFB1I1 |  |  |
| HSPB11 |  |  | NDUFC2-KCTD14 |  |  |
| CD55 |  |  | TOX4 |  |  |
| NCAPG2 |  |  | EPOR |  |  |
| OAS2 |  |  | C14orf79 |  |  |
| MRPL51 |  |  | TXNIP |  |  |
| F12 |  |  | EMC3 |  |  |
| AP1G1 |  |  | IL33 |  |  |
| ELF1 |  |  | RCAN1 |  |  |
| NGLY1 |  |  | PLEKHM3 |  |  |
| TMEM55B |  |  | LINC00969 |  |  |
| LINC01000 |  |  | ARNT |  |  |
| MAPK14 |  |  | EXOC7 |  |  |
| SRSF6 |  |  | ZFP36L2 |  |  |
| FDX1 |  |  | ZNF268 |  |  |
| NME1 |  |  | CHD2 |  |  |
| ICMT |  |  | LOC100233156 |  |  |
| NIFK |  |  | CEP112 |  |  |
| GZF1 |  |  | HNRNPD |  |  |
| PLEK |  |  | HLX |  |  |
| CCL3L3 |  |  | BNIP3L |  |  |
| EMP2 |  |  | MIA |  |  |
| FUNDC1 |  |  | FBXO28 |  |  |
| C12orf75 |  |  | PPP6R2 |  |  |
| VPS8 |  |  | KATNAL1 |  |  |
| NRIP1 |  |  | LRRFIP1 |  |  |
| FBLIM1 |  |  | TMEM136 |  |  |
| CAAP1 |  |  | GBE1 |  |  |
| EED |  |  | PPP2CA |  |  |
| ZDHHC4 |  |  | GIMAP6 |  |  |
| MGAT4A |  |  | CD209 |  |  |
| NT5DC1 |  |  | ALDH2 |  |  |
| SIGMAR1 |  |  | MIR7109 |  |  |
| PAWR |  |  | SERPINB6 |  |  |
| RNF170 |  |  | ARMCX3 |  |  |
| BCCIP |  |  | PTGER4 |  |  |
| CEP192 |  |  | CMYA5 |  |  |
| ACAD8 |  |  | PAOX |  |  |
| ANTXR1 |  |  | HIC1 |  |  |
| CAMSAP3 |  |  | KCNJ8 |  |  |
| LRCH3 |  |  | REEP5 |  |  |
| GPR160 |  |  | CNIH1 |  |  |
| HSPA6 |  |  | CFAP69 |  |  |
| CENPW |  |  | LOC102724229 |  |  |
| TANK |  |  | CDKL3 |  |  |
| CEP78 |  |  | LIX1L |  |  |
| SLC25A15 |  |  | LOC101930241 |  |  |
| POLM |  |  | ZDHHC3 |  |  |
| DNMT3A |  |  | MMP16 |  |  |
| TAPBP |  |  | IL6ST |  |  |
| KIN |  |  | SCAI |  |  |
| MTRR |  |  | ZNF767P |  |  |
| GPCPD1 |  |  | FAM129A |  |  |
| MASTL |  |  | KCTD12 |  |  |
| PRIM2B |  |  | GPX3 |  |  |
| PPP1R21 |  |  | QSOX1 |  |  |
| JUNB |  |  | RASA3 |  |  |
| TMEM254 |  |  | DTX3 |  |  |
| MIGA1 |  |  | LOC100507577 |  |  |
| AASDHPPT |  |  | HOXD8 |  |  |
| GRHL1 |  |  | CFL2 |  |  |
| RASA2 |  |  | STOM |  |  |
| GGCT |  |  | COL4A6 |  |  |
| FMNL2 |  |  | APOL3 |  |  |
| BNIP3 |  |  | GOLGA8A |  |  |
| MYSM1 |  |  | GAREM1 |  |  |
| YRDC |  |  | TAPT1-AS1 |  |  |
| HCCS |  |  | SNTB1 |  |  |
| SOS1 |  |  | CDC42BPB |  |  |
| MKRN2 |  |  | SETMAR |  |  |
| TRIM13 |  |  | CARD10 |  |  |
| ACSL3 |  |  | CRACR2B |  |  |
| ATP2B1 |  |  | SMIM3 |  |  |
| CD47 |  |  | KLF3-AS1 |  |  |
| GBAP1 |  |  | CROCCP2 |  |  |
| NADK |  |  | SKAP2 |  |  |
| SHB |  |  | CYB561D2 |  |  |
| DIRC2 |  |  | SERPINI1 |  |  |
| MAP1LC3B |  |  | PGAP1 |  |  |
| TRMT5 |  |  | ARPC4-TTLL3 |  |  |
| MCM6 |  |  | ACADL |  |  |
| MDK |  |  | C5orf63 |  |  |
| PDE4B |  |  | MAP1LC3A |  |  |
| SH3BGRL |  |  | LRP2 |  |  |
| SH3GLB2 |  |  | MSH5-SAPCD1 |  |  |
| PARP14 |  |  | HCG18 |  |  |
| FCER1G |  |  | SCGB1D2 |  |  |
| ARL6IP5 |  |  | MIR631 |  |  |
| ICE2 |  |  | ZNF141 |  |  |
| ABCC4 |  |  | PARD3B |  |  |
| METTL6 |  |  | PHLPP1 |  |  |
| CCL4 |  |  | NAV3 |  |  |
| POLDIP3 |  |  | C3 |  |  |
| CDC40 |  |  | RBPMS |  |  |
| SLC39A4 |  |  | NPIPB15 |  |  |
| ARHGDIB |  |  | LOC100505938 |  |  |
| PRIMPOL |  |  | GLYR1 |  |  |
| DIAPH3 |  |  | ZNF706 |  |  |
| ANAPC7 |  |  | USP13 |  |  |
| LSR |  |  | XDH |  |  |
| IL4I1 |  |  | KLHL22 |  |  |
| CDCA4 |  |  | EPN2-IT1 |  |  |
| RAP2C |  |  | ISM1 |  |  |
| ALG8 |  |  | RGPD2 |  |  |
| DEPDC1B |  |  | SFXN3 |  |  |
| HIST1H4J |  |  | FGD4 |  |  |
| MLF2 |  |  | CSPG4 |  |  |
| NR2F6 |  |  | LOC102724851 |  |  |
| DPY19L4 |  |  | ALDH1A1 |  |  |
| HIRA |  |  | SOX7 |  |  |
| C3orf80 |  |  | LOC283588 |  |  |
| QRSL1 |  |  | C3orf58 |  |  |
| CDC6 |  |  | TMCO4 |  |  |
| EXO1 |  |  | LOC105376805 |  |  |
| MBOAT2 |  |  | AVPI1 |  |  |
| EDNRA |  |  | ZDHHC2 |  |  |
| RASAL2 |  |  | KRT5 |  |  |
| PPIC |  |  | NASP |  |  |
| LOC105372255 |  |  | SLC25A11 |  |  |
| MIR7112 |  |  | CC2D2A |  |  |
| SLC25A46 |  |  | PER2 |  |  |
| EDC3 |  |  | KCNB1 |  |  |
| STK16 |  |  | LOC401317 |  |  |
| ZDHHC13 |  |  | MMAA |  |  |
| MMP14 |  |  | GNAQ |  |  |
| SLC39A10 |  |  | LOC100996740 |  |  |
| CHCHD7 |  |  | FGD2 |  |  |
| BRIP1 |  |  | NTRK3 |  |  |
| PIAS3 |  |  | EID1 |  |  |
| BCL2A1 |  |  | LAYN |  |  |
| RAB29 |  |  | SEPP1 |  |  |
| DDIAS |  |  | ALB |  |  |
| RAC2 |  |  | LOC102724851 |  |  |
| FLII |  |  | LRRC75B |  |  |
| C1orf174 |  |  | IGDCC3 |  |  |
| PRKAB2 |  |  | HIVEP3 |  |  |
| SLC19A1 |  |  | EMILIN2 |  |  |
| RSU1 |  |  | MAP1B |  |  |
| FRK |  |  | KIAA1755 |  |  |
| PPP3CB |  |  | TCF7L1 |  |  |
| CXCL11 |  |  | ITPR2 |  |  |
| TMEM132A |  |  | TWIST2 |  |  |
| ERP27 |  |  | DNM1 |  |  |
| FBXL6 |  |  | TNK2 |  |  |
| CENPL |  |  | PARP10 |  |  |
| SLC25A36 |  |  | SCGB2A2 |  |  |
| AKAP17A |  |  | C22orf39 |  |  |
| SEL1L3 |  |  | MYL5 |  |  |
| AMZ2 |  |  | TRO |  |  |
| SRGAP2C |  |  | IDNK |  |  |
| PQLC3 |  |  | YBX3 |  |  |
| LOC101929819 |  |  | NRN1 |  |  |
| BLM |  |  | MIR6845 |  |  |
| ZNF117 |  |  | JRKL |  |  |
| IRF9 |  |  | ETV6 |  |  |
| SRC |  |  | FOLR1 |  |  |
| EMC1 |  |  | APCDD1L |  |  |
| TFCP2 |  |  | SPHKAP |  |  |
| DENND1B |  |  | COPZ2 |  |  |
| IGHV3-23 |  |  | FAM69A |  |  |
| SAR1A |  |  | IFFO2 |  |  |
| THAP9-AS1 |  |  | TPPP3 |  |  |
| MMP1 |  |  | CPXM1 |  |  |
| COL4A2 |  |  | TP53I13 |  |  |
| FAM63B |  |  | MBD6 |  |  |
| THEMIS2 |  |  | LLPH |  |  |
| POLE2 |  |  | CDCA7L |  |  |
| PALLD |  |  | NYNRIN |  |  |
| TPM1 |  |  | TPR |  |  |
| RAB23 |  |  | SAMD4A |  |  |
| HOTAIR |  |  | P2RY14 |  |  |
| ASPH |  |  | RGCC |  |  |
| DARS2 |  |  | AXL |  |  |
| ABCC1 |  |  | EGR1 |  |  |
| KTI12 |  |  | ALDOC |  |  |
| ZNF277 |  |  | ARMC8 |  |  |
| HAUS1 |  |  | CBR3 |  |  |
| LCP2 |  |  | ATG5 |  |  |
| ZKSCAN8 |  |  | ENAH |  |  |
| HNRNPA1 |  |  | P3H2 |  |  |
| SLC44A1 |  |  | DIP2C |  |  |
| FLT1 |  |  | ANGPTL2 |  |  |
| MRPS23 |  |  | NDUFB8 |  |  |
| NBPF20 |  |  | WFS1 |  |  |
| FCGR2C |  |  | CDK13 |  |  |
| IKBKB |  |  | CHD3 |  |  |
| CASP6 |  |  | C1orf21 |  |  |
| CDCA8 |  |  | ADARB1 |  |  |
| TAF1B |  |  | GLRX3 |  |  |
| BLMH |  |  | OLFM4 |  |  |
| GOT1 |  |  | NEK3 |  |  |
| STIL |  |  | ANGPT1 |  |  |
| GLUD1 |  |  | THNSL2 |  |  |
| CASZ1 |  |  | RAMP3 |  |  |
| ASXL1 |  |  | KIAA0232 |  |  |
| PHB |  |  | SFT2D3 |  |  |
| MORC2 |  |  | SPAG16 |  |  |
| RNF19A |  |  | IL22RA2 |  |  |
| NF1P9 |  |  | ZNF615 |  |  |
| FOXK2 |  |  | RIPK4 |  |  |
| EXPH5 |  |  | SYMPK |  |  |
| STAT1 |  |  | SLC25A3 |  |  |
| DBT |  |  | KCNT2 |  |  |
| CYC1 |  |  | ITGA6 |  |  |
| CTAGE8 |  |  | PPP1R3E |  |  |
| SLC7A1 |  |  | FMO1 |  |  |
| SCO2 |  |  | ATP9B |  |  |
| AP2A1 |  |  | TAPT1 |  |  |
| SLC16A1 |  |  | RAD51B |  |  |
| EPPK1 |  |  | ZNF704 |  |  |
| CBX2 |  |  | ASAP3 |  |  |
| CCNYL1 |  |  | SMOC2 |  |  |
| DNPEP |  |  | DNMBP |  |  |
| ARHGAP18 |  |  | HSPB2-C11orf52 |  |  |
| MAP2K5 |  |  | IFT88 |  |  |
| GXYLT2 |  |  | MGMT |  |  |
| MIR637 |  |  | LINC00899 |  |  |
| WTH3DI |  |  | ARMCX6 |  |  |
| PRKAA1 |  |  | CALHM2 |  |  |
| CIB1 |  |  | MYRIP |  |  |
| FNTA |  |  | AKAP7 |  |  |
| TWSG1 |  |  | A2M |  |  |
| ZCCHC2 |  |  | SOCS2 |  |  |
| MGA |  |  | CYP4V2 |  |  |
| TMEM123 |  |  | NSF |  |  |
| KAT6B |  |  | PTPRM |  |  |
| DNAH14 |  |  | ARHGAP5 |  |  |
| ZNF165 |  |  | TTYH2 |  |  |
| PRPS1 |  |  | LOC101928524 |  |  |
| FXR1 |  |  | LIMCH1 |  |  |
| UTP23 |  |  | CBR4 |  |  |
| LOC101928195 |  |  | ADGRL1 |  |  |
| TMEM65 |  |  | AKAP1 |  |  |
| DHX40 |  |  | ACKR4 |  |  |
| NEK2 |  |  | NEO1 |  |  |
| HIST2H4B |  |  | CFAP70 |  |  |
| SOCS3 |  |  | STRN |  |  |
| IFI6 |  |  | ADORA1 |  |  |
| OSGIN2 |  |  | ADAMTS1 |  |  |
| TPI1 |  |  | PGM1 |  |  |
| WDYHV1 |  |  | TBC1D16 |  |  |
| EFHD2 |  |  | SNORD50B |  |  |
| MALT1 |  |  | ZNF688 |  |  |
| CUEDC1 |  |  | PNISR |  |  |
| UCP2 |  |  | LOC644172 |  |  |
| HEXIM1 |  |  | SESN1 |  |  |
| NEDD4 |  |  | SOX4 |  |  |
| GPR137B |  |  | EPHB1 |  |  |
| SLC44A4 |  |  | TSPAN31 |  |  |
| GLTP |  |  | IVD |  |  |
| ZKSCAN5 |  |  | TFAP2C |  |  |
| PIK3R3 |  |  | DBT |  |  |
| MRPS17 |  |  | MSRB3 |  |  |
| PLXNB1 |  |  | ABCA3 |  |  |
| PKP4 |  |  | EPB41 |  |  |
| FKBP5 |  |  | LANCL2 |  |  |
| BAG1 |  |  | C1QTNF1 |  |  |
| GTF2E2 |  |  | LOC286191 |  |  |
| OCLN |  |  | PRRG1 |  |  |
| NOL9 |  |  | N4BP2L2 |  |  |
| HAUS7 |  |  | LTF |  |  |
| DIO2 |  |  | RPS6KA5 |  |  |
| SRPK1 |  |  | ROPN1 |  |  |
| DEPDC1 |  |  | PDE4A |  |  |
| ACER3 |  |  | ZFHX3 |  |  |
| KYNU |  |  | ZNF521 |  |  |
| HLA-DPA1 |  |  | CROT |  |  |
| ATPIF1 |  |  | ACADSB |  |  |
| TOMM5 |  |  | AGAP1 |  |  |
| HIPK2 |  |  | COL5A3 |  |  |
| ABCG1 |  |  | FOXP1-IT1 |  |  |
| UCHL3 |  |  | KDR |  |  |
| JADE3 |  |  | CYTH4 |  |  |
| FICD |  |  | MXI1 |  |  |
| SAMD9L |  |  | LOC101927841 |  |  |
| CEP41 |  |  | MFAP4 |  |  |
| PPP2R5E |  |  | MRC1 |  |  |
| CLTC |  |  | NOSTRIN |  |  |
| SAMSN1 |  |  | EHHADH |  |  |
| NOP16 |  |  | PARP11 |  |  |
| RNFT1 |  |  | CSTA |  |  |
| C1QTNF6 |  |  | DYNC2H1 |  |  |
| ATP11B |  |  | MYLIP |  |  |
| RBM6 |  |  | PDGFRL |  |  |
| CSTB |  |  | ROPN1B |  |  |
| PMAIP1 |  |  | POLI |  |  |
| CCL2 |  |  | C15orf52 |  |  |
| RGS4 |  |  | CYHR1 |  |  |
| RAB11FIP1 |  |  | LPIN1 |  |  |
| EGLN1 |  |  | RAPH1 |  |  |
| CNOT9 |  |  | LINC00888 |  |  |
| MIIP |  |  | IRAK3 |  |  |
| LPXN |  |  | TCEAL2 |  |  |
| ZNF93 |  |  | NPDC1 |  |  |
| EPN3 |  |  | OSR1 |  |  |
| SMARCA2 |  |  | TNFRSF10D |  |  |
| LUC7L3 |  |  | BEX2 |  |  |
| GLMP |  |  | MEIS1 |  |  |
| PCBD1 |  |  | DIXDC1 |  |  |
| DDIT4 |  |  | BHLHE22 |  |  |
| INSIG2 |  |  | RNF125 |  |  |
| NUS1 |  |  | LOC729732 |  |  |
| SNORD77 |  |  | RASD1 |  |  |
| RNF130 |  |  | LINC01279 |  |  |
| NCAPH |  |  | HSD17B8 |  |  |
| RBMS1 |  |  | VGLL3 |  |  |
| PLXDC2 |  |  | MB21D2 |  |  |
| BMP1 |  |  | FAH |  |  |
| ZYG11B |  |  | GK |  |  |
| GON4L |  |  | GSTZ1 |  |  |
| CD99 |  |  | FAM150B |  |  |
| GLRX2 |  |  | STAG3L3 |  |  |
| THY1 |  |  | FAM120C |  |  |
| COL1A1 |  |  | LINC00597 |  |  |
| FAM117B |  |  | MR1 |  |  |
| SENP2 |  |  | GPCPD1 |  |  |
| TNFSF4 |  |  | PEX11A |  |  |
| SLC7A11 |  |  | LY75 |  |  |
| CTSC |  |  | ZNF84 |  |  |
| WWC3 |  |  | PELI1 |  |  |
| VDR |  |  | KLF8 |  |  |
| TSPAN3 |  |  | WWC1 |  |  |
| ID2 |  |  | STEAP4 |  |  |
| RPS6KA3 |  |  | ACSS1 |  |  |
| HOPX |  |  | L3MBTL4 |  |  |
| LDAH |  |  | GARNL3 |  |  |
| RAD51C |  |  | WDR19 |  |  |
| MEX3A |  |  | TMEM163 |  |  |
| OPN3 |  |  | ANAPC5 |  |  |
| GFPT2 |  |  | LOC400043 |  |  |
| RPP40 |  |  | LOC100507855 |  |  |
| LOC202181 |  |  | KCNN4 |  |  |
| EMC9 |  |  | C8orf58 |  |  |
| GBP5 |  |  | GRB10 |  |  |
| DUSP10 |  |  | SAMHD1 |  |  |
| LAD1 |  |  | HIC2 |  |  |
| CHEK2 |  |  | TNNI2 |  |  |
| NUDT5 |  |  | FBXO2 |  |  |
| HAUS6 |  |  | SEMA3F |  |  |
| PIGW |  |  | RPS21 |  |  |
| PRKAB1 |  |  | MIR4683 |  |  |
| IGFBP3 |  |  | PEG3 |  |  |
| MTFR2 |  |  | PROM1 |  |  |
| HILPDA |  |  | MIR6840 |  |  |
| C1GALT1 |  |  | FAM43A |  |  |
| BIVM |  |  | JAML |  |  |
| PLK4 |  |  | TIMP2 |  |  |
| UQCR10 |  |  | ELP2 |  |  |
| IGHV4-31 |  |  | PAPLN |  |  |
| PLSCR1 |  |  | LPAR6 |  |  |
| NR1D2 |  |  | NXN |  |  |
| RNF213 |  |  | TNFRSF21 |  |  |
| EPSTI1 |  |  | MRPS14 |  |  |
| GAB1 |  |  | PHLDA3 |  |  |
| SLC2A10 |  |  | RPS16P5 |  |  |
| CENPN |  |  | PDE5A |  |  |
| MEX3D |  |  | COBLL1 |  |  |
| RNF24 |  |  | CDC42BPA |  |  |
| GGH |  |  | ZNF14 |  |  |
| SDF2L1 |  |  | TMEM117 |  |  |
| OTULIN |  |  | OLFML1 |  |  |
| ACTR5 |  |  | NR2F2 |  |  |
| ERC1 |  |  | LOC389765 |  |  |
| MCUB |  |  | ARHGAP26 |  |  |
| GRTP1 |  |  | GGT6 |  |  |
| ACOT13 |  |  | PODN |  |  |
| IL1RN |  |  | ISLR |  |  |
| NUDT1 |  |  | SPATA18 |  |  |
| IFI44L |  |  | PAN2 |  |  |
| PITX1 |  |  | ECHDC1 |  |  |
| HBS1L |  |  | MPZL2 |  |  |
| MMP3 |  |  | FAM134B |  |  |
| LCP1 |  |  | CLMP |  |  |
| TLR1 |  |  | SLC28A3 |  |  |
| PCYOX1 |  |  | NFASC |  |  |
| MCM7 |  |  | INHBB |  |  |
| C5AR1 |  |  | GABPB1-AS1 |  |  |
| ATP8B1 |  |  | PLCB1 |  |  |
| MSR1 |  |  | NFATC2 |  |  |
| FAR2 |  |  | RPH3AL |  |  |
| VASH1 |  |  | ADRB1 |  |  |
| TP53BP2 |  |  | MXRA8 |  |  |
| GLIPR1 |  |  | ITGB4 |  |  |
| MED30 |  |  | ANXA8L1 |  |  |
| SLC6A6 |  |  | SDC3 |  |  |
| SYNGR2 |  |  | FZD1 |  |  |
| PRR15 |  |  | IRX2 |  |  |
| CAMK2D |  |  | ARHGAP23 |  |  |
| RAPH1 |  |  | ZNF559 |  |  |
| CASD1 |  |  | LOC100507577 |  |  |
| CPPED1 |  |  | CIB2 |  |  |
| RALB |  |  | LSP1 |  |  |
| CCAR1 |  |  | HAPLN3 |  |  |
| RRS1 |  |  | SRSF1 |  |  |
| GEMIN2 |  |  | IGHA2 |  |  |
| CD74 |  |  | ACSS3 |  |  |
| SLC12A8 |  |  | APOLD1 |  |  |
| ZMYND8 |  |  | TPTEP1 |  |  |
| ZNF514 |  |  | C1orf116 |  |  |
| COG1 |  |  | EGFR |  |  |
| ERLIN1 |  |  | STON1 |  |  |
| KIF3C |  |  | FOXC1 |  |  |
| CLN6 |  |  | APOL6 |  |  |
| NINJ2 |  |  | MCOLN3 |  |  |
| RAD18 |  |  | EFNA5 |  |  |
| PILRA |  |  | MFSD4A |  |  |
| MLF1 |  |  | KLHDC1 |  |  |
| SNRPA1 |  |  | ADD3 |  |  |
| IRX5 |  |  | PDGFRB |  |  |
| YEATS4 |  |  | SIRPA |  |  |
| LOC101928615 |  |  | PADI2 |  |  |
| PUDP |  |  | ANG |  |  |
| SLC35F2 |  |  | CX3CR1 |  |  |
| PGM2 |  |  | DUSP6 |  |  |
| MMP9 |  |  | GPR180 |  |  |
| SIK3 |  |  | ERO1B |  |  |
| MSMO1 |  |  | LAMB1 |  |  |
| MST1R |  |  | ASPH |  |  |
| DDX11 |  |  | GLIPR2 |  |  |
| LNPEP |  |  | RGMB |  |  |
| SAMD12 |  |  | FLJ37453 |  |  |
| COTL1 |  |  | DTX4 |  |  |
| IGHD |  |  | MAP3K6 |  |  |
| CDC25C |  |  | CITED4 |  |  |
| SLC5A3 |  |  | STMN3 |  |  |
| NFE2L3 |  |  | WWOX |  |  |
| CEMIP |  |  | KDM4B |  |  |
| CDCA7 |  |  | RAI2 |  |  |
| STMN1 |  |  | FABP7 |  |  |
| SPC25 |  |  | YBEY |  |  |
| CMC2 |  |  | FAT4 |  |  |
| MIR4680 |  |  | C12orf66 |  |  |
| CIART |  |  | PLXND1 |  |  |
| HDLBP |  |  | GMFG |  |  |
| SLAMF8 |  |  | S100A1 |  |  |
| DIP2C |  |  | PTGES |  |  |
| PDCD2L |  |  | FAM171A1 |  |  |
| IFI44 |  |  | ZRANB1 |  |  |
| CEP57 |  |  | PLEKHS1 |  |  |
| OGDH |  |  | EGR2 |  |  |
| IFRD1 |  |  | CCAR2 |  |  |
| RSL1D1 |  |  | C14orf159 |  |  |
| TIMP3 |  |  | C1orf226 |  |  |
| DNAJC15 |  |  | RFX3 |  |  |
| DNAJB6 |  |  | GGT2 |  |  |
| MFSD3 |  |  | SLPI |  |  |
| PPM1D |  |  | TBC1D24 |  |  |
| PSD3 |  |  | GKAP1 |  |  |
| DAPK1-IT1 |  |  | MAPT |  |  |
| SYNRG |  |  | RPL31 |  |  |
| BEND3 |  |  | LDHB |  |  |
| SRSF8 |  |  | TIMP3 |  |  |
| UPK3BL |  |  | ATP6AP1L |  |  |
| LOX |  |  | AZGP1 |  |  |
| HMGCS1 |  |  | HEXDC |  |  |
| TEX30 |  |  | SLC27A3 |  |  |
| SLC3A2 |  |  | FRY |  |  |
| ST14 |  |  | TNFRSF25 |  |  |
| IGHV3-23 |  |  | GRAMD2 |  |  |
| CTXN1 |  |  | ADRB2 |  |  |
| SLC25A37 |  |  | EPS8L2 |  |  |
| USP18 |  |  | FLI1 |  |  |
| HMGB2 |  |  | LALBA |  |  |
| ATAD3B |  |  | PDZD2 |  |  |
| PARD6B |  |  | BBOX1 |  |  |
| PLEKHA2 |  |  | DUBR |  |  |
| CTGF |  |  | KLF2 |  |  |
| LOC101930415 |  |  | SERPING1 |  |  |
| OAZ3 |  |  | JCHAIN |  |  |
| ADAM12 |  |  | STC2 |  |  |
| TP53 |  |  | NUDT11 |  |  |
| CAP2 |  |  | KLK7 |  |  |
| SKA2 |  |  | RBMS2 |  |  |
| MTERF2 |  |  | APBB2 |  |  |
| GNAS |  |  | TPPP |  |  |
| MAP3K2 |  |  | NEDD9 |  |  |
| GINS3 |  |  | MAOB |  |  |
| MIR1178 |  |  | PRKAG2-AS1 |  |  |
| PTPRC |  |  | DKFZP564C152 |  |  |
| TMEM68 |  |  | KANK4 |  |  |
| CTHRC1 |  |  | TIAM2 |  |  |
| TNFRSF21 |  |  | PIWIL4 |  |  |
| TUFT1 |  |  | AMOTL2 |  |  |
| SEPHS1 |  |  | SNURF |  |  |
| LINC01094 |  |  | TMOD1 |  |  |
| ARFGEF3 |  |  | ACSL5 |  |  |
| ATP6V1H |  |  | PWAR6 |  |  |
| TCAIM |  |  | KALRN |  |  |
| CMAS |  |  | RORC |  |  |
| MIR15A |  |  | KCTD1 |  |  |
| TRAC |  |  | AS3MT |  |  |
| PLGRKT |  |  | CDC14B |  |  |
| LOC101926921 |  |  | PLEKHF1 |  |  |
| SMG1P5 |  |  | SNCAIP |  |  |
| DNAJC4 |  |  | C11orf63 |  |  |
| CBS |  |  | HEY2 |  |  |
| AGPS |  |  | XIST |  |  |
| PRKACB |  |  | C16orf46 |  |  |
| FAM64A |  |  | DOCK11 |  |  |
| MIPEP |  |  | IL27RA |  |  |
| FAXC |  |  | BCL11A |  |  |
| CASC7 |  |  | FTX |  |  |
| TYMP |  |  | SLC16A4 |  |  |
| SRGN |  |  | ZNF44 |  |  |
| SPA17 |  |  | MEST |  |  |
| TMEM35B |  |  | MIR6872 |  |  |
| SMIM14 |  |  | CAPS2 |  |  |
| MINA |  |  | PCGF2 |  |  |
| FERMT2 |  |  | C14orf132 |  |  |
| ST6GALNAC4 |  |  | MIR452 |  |  |
| ZBTB10 |  |  | MYH14 |  |  |
| DBNDD1 |  |  | VWA5A |  |  |
| RSAD2 |  |  | EFS |  |  |
| GPR68 |  |  | AGTR1 |  |  |
| RECQL4 |  |  | PLXDC1 |  |  |
| NAIP |  |  | LOC101926921 |  |  |
| EIF3C |  |  | MINOS1-NBL1 |  |  |
| SH3TC1 |  |  | ZNF626 |  |  |
| WIPF2 |  |  | WDR11 |  |  |
| CENPI |  |  | RUNX3 |  |  |
| FAM86DP |  |  | THSD4 |  |  |
| C15orf48 |  |  | TRIM2 |  |  |
| CCNE1 |  |  | LOC102724250 |  |  |
| TRAC |  |  | CFLAR |  |  |
| MANEAL |  |  | SERPINB9 |  |  |
| DNMT3B |  |  | SYNE1 |  |  |
| TMEM200A |  |  | CSN1S1 |  |  |
| FAM210B |  |  | PLEKHA6 |  |  |
| BTG3 |  |  | PIK3CD |  |  |
| ZNF639 |  |  | MT1E |  |  |
| C16orf87 |  |  | LINC00173 |  |  |
| FUBP3 |  |  | HOXB-AS1 |  |  |
| CENPBD1 |  |  | EPB41L1 |  |  |
| IFIT2 |  |  | ABCC3 |  |  |
| ADGRG1 |  |  | NBEA |  |  |
| IGSF9 |  |  | KCNMB1 |  |  |
| JAK2 |  |  | PNPLA4 |  |  |
| PKIB |  |  | ANO1 |  |  |
| CNOT7 |  |  | GSTT1 |  |  |
| LILRB4 |  |  | ATP13A5 |  |  |
| CCDC125 |  |  | ARMC9 |  |  |
| ZNF83 |  |  | SGK223 |  |  |
| S100A8 |  |  | NOVA1 |  |  |
| PSMB3 |  |  | LINC00849 |  |  |
| SLFN5 |  |  | ZSCAN18 |  |  |
| CELSR1 |  |  | HEPH |  |  |
| RPS6KA1 |  |  | XYLT1 |  |  |
| USP1 |  |  | MEIOC |  |  |
| MND1 |  |  | PGF |  |  |
| INAFM2 |  |  | TFAP2B |  |  |
| EPB41L5 |  |  | WFDC2 |  |  |
| KLHL42 |  |  | CAND2 |  |  |
| POLQ |  |  | PRKAR2B |  |  |
| LANCL2 |  |  | LOC101930400 |  |  |
| DPP7 |  |  | AZGP1P1 |  |  |
| DAPP1 |  |  | PPID |  |  |
| MCM5 |  |  | KATNB1 |  |  |
| ECE2 |  |  | NUDT7 |  |  |
| SRD5A1 |  |  | CHPT1 |  |  |
| CSTF3 |  |  | THEM4 |  |  |
| MAP9 |  |  | PTPRZ1 |  |  |
| COX20 |  |  | TMEM204 |  |  |
| U2SURP |  |  | IGHD |  |  |
| MXD1 |  |  | ZEB1 |  |  |
| SLC6A8 |  |  | LFNG |  |  |
| STARD10 |  |  | CYP4F22 |  |  |
| SPCS3 |  |  | SESTD1 |  |  |
| ST8SIA6-AS1 |  |  | HSD11B1 |  |  |
| IKBIP |  |  | MAML3 |  |  |
| EFNA4 |  |  | CTNNAL1 |  |  |
| SLC38A10 |  |  | ETV1 |  |  |
| THEM6 |  |  | CRACR2A |  |  |
| TRIM14 |  |  | FBXL20 |  |  |
| ABHD5 |  |  | KCNMA1 |  |  |
| MAPRE2 |  |  | TPM1 |  |  |
| TIFA |  |  | FRMD6 |  |  |
| DACT1 |  |  | ATP8A1 |  |  |
| REEP1 |  |  | LGR4 |  |  |
| BCL2L13 |  |  | FGFR2 |  |  |
| RUNX2 |  |  | TMEM71 |  |  |
| SLC43A3 |  |  | ZMAT1 |  |  |
| PRR5-ARHGAP8 |  |  | PIP4K2B |  |  |
| ARHGAP5 |  |  | TOP1MT |  |  |
| TRIB3 |  |  | TP53 |  |  |
| PLA2G7 |  |  | ERBB4 |  |  |
| PRSS23 |  |  | ADM |  |  |
| LRRC15 |  |  | DEFB1 |  |  |
| LOC101060835 |  |  | SOSTDC1 |  |  |
| HIST1H2BC |  |  | ARHGAP25 |  |  |
| RPL39L |  |  | SIM1 |  |  |
| IGHV3-23 |  |  | GLI3 |  |  |
| JMJD4 |  |  | HSD17B11 |  |  |
| COL8A2 |  |  | ENTPD1 |  |  |
| PRRG4 |  |  | POU2AF1 |  |  |
| KLHL23 |  |  | C1R |  |  |
| SPRED2 |  |  | IGF1R |  |  |
| NETO2 |  |  | PLAT |  |  |
| MIR155 |  |  | LINC01138 |  |  |
| TXNDC16 |  |  | SLC6A14 |  |  |
| XIST |  |  | LOC106146153 |  |  |
| GMPS |  |  | ZNF711 |  |  |
| LAPTM5 |  |  | ZNF117 |  |  |
| TKT |  |  | LOC100130872 |  |  |
| FLOT2 |  |  | PM20D2 |  |  |
| HIST1H4H |  |  | LINC00993 |  |  |
| ADAM15 |  |  | FBLN2 |  |  |
| TFEC |  |  | FKBP1B |  |  |
| ISG15 |  |  | PABPC1L |  |  |
| IRF1 |  |  | CNTN4 |  |  |
| IQCG |  |  | CD320 |  |  |
| PDGFC |  |  | TUBB2B |  |  |
| MAL2 |  |  | LOC100507424 |  |  |
| SOX11 |  |  | FMNL3 |  |  |
| CA12 |  |  | ARHGAP21 |  |  |
| IRF7 |  |  | MCF2L |  |  |
| CXADR |  |  | FRS2 |  |  |
| TMTC4 |  |  | ZNF559-ZNF177 |  |  |
| GRB7 |  |  | SNED1 |  |  |
| CDCA2 |  |  | ARRDC4 |  |  |
| OVOL1 |  |  | JADE2 |  |  |
| RAC3 |  |  | METTL10 |  |  |
| LOXL2 |  |  | LINC01410 |  |  |
| ALDH1B1 |  |  | FAM133A |  |  |
| TBL1X |  |  | SLC6A16 |  |  |
| MED1 |  |  | TM4SF1 |  |  |
| SLC2A13 |  |  | FBXO32 |  |  |
| ARHGEF9 |  |  | IDUA |  |  |
| G6PD |  |  | AR |  |  |
| CST1 |  |  | CLSTN2 |  |  |
| SLC1A4 |  |  | ADAMTS15 |  |  |
| ZFP36L1 |  |  | MPV17L |  |  |
| FOXA1 |  |  | SHANK2 |  |  |
| GALNT18 |  |  | BEX5 |  |  |
| RDH10 |  |  | CCND2 |  |  |
| FBXL20 |  |  | PRKG1 |  |  |
| ADCY7 |  |  | GPD1L |  |  |
| SCCPDH |  |  | AOX1 |  |  |
| HSPA1L |  |  | SELENBP1 |  |  |
| CYBB |  |  | ABCB6 |  |  |
| ATP2C2 |  |  | GABBR1 |  |  |
| ARNT2 |  |  | CLMN |  |  |
| TUBD1 |  |  | WASF3 |  |  |
| ITPRIPL2 |  |  | NLGN4X |  |  |
| CDKL5 |  |  | RERG |  |  |
| ETS2 |  |  | PTCHD1 |  |  |
| ANKRD10-IT1 |  |  | PHYHD1 |  |  |
| TMEM170A |  |  | FAM3B |  |  |
| NFIB |  |  | CST3 |  |  |
| HACD2 |  |  | H2AFJ |  |  |
| BCKDHB |  |  | CHST2 |  |  |
| PDLIM7 |  |  | FAM84A |  |  |
| CST6 |  |  | CRIPAK |  |  |
| DOCK10 |  |  | SLC12A2 |  |  |
| HIST1H3F |  |  | LOC102724197 |  |  |
| CDKAL1 |  |  | SLC24A3 |  |  |
| SCRN1 |  |  | ZNF703 |  |  |
| BICDL1 |  |  | PRTFDC1 |  |  |
| STX1A |  |  | FAM198B |  |  |
| ARHGEF7 |  |  | PLTP |  |  |
| PLS1 |  |  | GCNT2 |  |  |
| CNOT1 |  |  | RRAS2 |  |  |
| LYZ |  |  | GAS1 |  |  |
| PCTP |  |  | NINL |  |  |
| SGMS2 |  |  | DGKE |  |  |
| MYLIP |  |  | COL4A2 |  |  |
| LOC642846 |  |  | TCEA2 |  |  |
| NME7 |  |  | HDGFRP3 |  |  |
| CRIP2 |  |  | OLFML3 |  |  |
| CHAF1A |  |  | SLC7A2 |  |  |
| MTMR1 |  |  | RRAGD |  |  |
| NAT1 |  |  | CSAD |  |  |
| CD69 |  |  | PTX3 |  |  |
| FPR1 |  |  | RNF183 |  |  |
| HIST1H2AM |  |  | KIAA1324L |  |  |
| GPX8 |  |  | PARM1 |  |  |
| MOCOS |  |  | TGFBR1 |  |  |
| MIR1204 |  |  | GFRA1 |  |  |
| GDPD1 |  |  | C19orf18 |  |  |
| EXOC6 |  |  | FAR2P3 |  |  |
| RTN4IP1 |  |  | LOC727820 |  |  |
| KMO |  |  | SDC2 |  |  |
| SLC39A8 |  |  | MGP |  |  |
| TJP3 |  |  | TLE1 |  |  |
| SSFA2 |  |  | MIR17HG |  |  |
| HIST1H2AE |  |  | ZC3H12C |  |  |
| TDO2 |  |  | RHOV |  |  |
| TRIB2 |  |  | MAN1A1 |  |  |
| MUC1 |  |  | DTNA |  |  |
| ELP5 |  |  | RAB30 |  |  |
| NMU |  |  | NTN4 |  |  |
| DDX60 |  |  | F3 |  |  |
| HIST1H1C |  |  | MFAP5 |  |  |
| C1orf106 |  |  | IGFBP5 |  |  |
| LINC00467 |  |  | PHGDH |  |  |
| ST6GAL2 |  |  | KIAA1324 |  |  |
| C1orf53 |  |  | SOX6 |  |  |
| BTBD19 |  |  | LOC101928916 |  |  |
| ADH5 |  |  | EPHX2 |  |  |
| MMP13 |  |  | ZNF667-AS1 |  |  |
| SETD9 |  |  | ATL1 |  |  |
| TMEM45A |  |  | NPR3 |  |  |
| C8orf44-SGK3 |  |  | LOC102724951 |  |  |
| GCA |  |  | IGFBP3 |  |  |
| PTGS1 |  |  | MMP7 |  |  |
| FOS |  |  | YPEL2 |  |  |
| TMC5 |  |  | NSMCE4A |  |  |
| KIAA1324 |  |  | ELF3 |  |  |
| NID1 |  |  | KIAA0485 |  |  |
| KCNK1 |  |  | LRIG1 |  |  |
| COL5A2 |  |  | GDPD3 |  |  |
| RCAN1 |  |  | SFRP4 |  |  |
| TNFSF13B |  |  | HMGCS2 |  |  |
| GSR |  |  | MET |  |  |
| LOC100129518 |  |  | LNX1 |  |  |
| OLR1 |  |  | IGF2BP2 |  |  |
| SELL |  |  | S100A10 |  |  |
| C3orf14 |  |  | F8 |  |  |
| PTRH2 |  |  | RFTN1 |  |  |
| ICA1 |  |  | RAB7B |  |  |
| RND1 |  |  | FGL2 |  |  |
| SOCS1 |  |  | PPP1R1B |  |  |
| ZNF655 |  |  | IGLL3P |  |  |
| SLC2A5 |  |  | SNORA11E |  |  |
| OSBPL6 |  |  | CALB2 |  |  |
| CACNA1D |  |  | F13A1 |  |  |
| TCEAL8 |  |  | STEAP2 |  |  |
| BTG2 |  |  | LRRC17 |  |  |
| PEX11A |  |  | BICC1 |  |  |
| IGK |  |  | HCAR3 |  |  |
| SERTAD4 |  |  | SGCD |  |  |
| FANCD2 |  |  | GLT8D2 |  |  |
| WWTR1 |  |  | HTRA1 |  |  |
| CMPK2 |  |  | MEGF10 |  |  |
| VEZT |  |  | GLB1L2 |  |  |
| HINT3 |  |  | MGST1 |  |  |
| LINC00673 |  |  | ITGB8 |  |  |
| CYB561 |  |  | SPARC |  |  |
| C5orf46 |  |  |  |  |  |
| EPB41L3 |  |  |  |  |  |
| SPP1 |  |  |  |  |  |
| GM2A |  |  |  |  |  |
| MACC1 |  |  |  |  |  |
| MIEN1 |  |  |  |  |  |
| RARRES1 |  |  |  |  |  |
| FZD6 |  |  |  |  |  |
| ANXA9 |  |  |  |  |  |
| RNFT2 |  |  |  |  |  |
| EMB |  |  |  |  |  |
| B2M |  |  |  |  |  |
| PSMB8 |  |  |  |  |  |
| PSAT1 |  |  |  |  |  |
| TANC2 |  |  |  |  |  |
| ATP1B1 |  |  |  |  |  |
| PRLR |  |  |  |  |  |
| LOC100509457 |  |  |  |  |  |
| PREX1 |  |  |  |  |  |
| GDF15 |  |  |  |  |  |
| EGLN3 |  |  |  |  |  |
| POSTN |  |  |  |  |  |
| DPYSL3 |  |  |  |  |  |
| SCD |  |  |  |  |  |
| HIST1H3F |  |  |  |  |  |
| ITPR1 |  |  |  |  |  |
| LAMP3 |  |  |  |  |  |
| SLC16A6 |  |  |  |  |  |
| RPS24 |  |  |  |  |  |
| CAMK2N1 |  |  |  |  |  |
| CD40 |  |  |  |  |  |
| EME1 |  |  |  |  |  |
| PTK6 |  |  |  |  |  |
| LOC100505984 |  |  |  |  |  |
| HCK |  |  |  |  |  |
| CCR1 |  |  |  |  |  |
| HMGB3P1 |  |  |  |  |  |
| COL5A1 |  |  |  |  |  |
| RRAS2 |  |  |  |  |  |
| CTSV |  |  |  |  |  |
| CLN8 |  |  |  |  |  |
| SLC39A11 |  |  |  |  |  |
| CYTIP |  |  |  |  |  |
| SDC2 |  |  |  |  |  |
| ZYG11A |  |  |  |  |  |
| PEG10 |  |  |  |  |  |
| KLHDC7B |  |  |  |  |  |
| RHOH |  |  |  |  |  |
| BCAT1 |  |  |  |  |  |
| TRMT2B |  |  |  |  |  |
| FAM84A |  |  |  |  |  |
| TRIM16 |  |  |  |  |  |
| CXCL8 |  |  |  |  |  |
| ARSG |  |  |  |  |  |
| CD37 |  |  |  |  |  |
| PXMP4 |  |  |  |  |  |
| PLK1 |  |  |  |  |  |
| CAPS |  |  |  |  |  |
| EIF4B |  |  |  |  |  |
| CDCP1 |  |  |  |  |  |
| RGS5 |  |  |  |  |  |
| LDLRAD4 |  |  |  |  |  |
| S100A9 |  |  |  |  |  |
| CEACAM6 |  |  |  |  |  |
| RET |  |  |  |  |  |
| TSPAN1 |  |  |  |  |  |
| OASL |  |  |  |  |  |
| CYBA |  |  |  |  |  |
| PLCH1 |  |  |  |  |  |
| ADAMDEC1 |  |  |  |  |  |
| ITPR2 |  |  |  |  |  |
| BMPR1B |  |  |  |  |  |
| PPM1H |  |  |  |  |  |
| RNGTT |  |  |  |  |  |
| LRP8 |  |  |  |  |  |
| CLIC3 |  |  |  |  |  |
| WNT5A |  |  |  |  |  |
| UBE2L6 |  |  |  |  |  |
| MYBL1 |  |  |  |  |  |
| AIM2 |  |  |  |  |  |
| LOC102724387 |  |  |  |  |  |
| TBC1D9 |  |  |  |  |  |
| CTSO |  |  |  |  |  |
| IMPA2 |  |  |  |  |  |
| MED13L |  |  |  |  |  |
| SLC2A6 |  |  |  |  |  |
| RAB26 |  |  |  |  |  |
| CA2 |  |  |  |  |  |
| TTLL7 |  |  |  |  |  |
| SMARCA1 |  |  |  |  |  |
| GIMAP2 |  |  |  |  |  |
| STK26 |  |  |  |  |  |
| CNTNAP2 |  |  |  |  |  |
| MLLT11 |  |  |  |  |  |
| SLC12A2 |  |  |  |  |  |
| GPC4 |  |  |  |  |  |
| GBP1 |  |  |  |  |  |
| DOCK5 |  |  |  |  |  |
| TMSB15B |  |  |  |  |  |
| IGHV4-31 |  |  |  |  |  |
| SPAG4 |  |  |  |  |  |
| FTX |  |  |  |  |  |
| ITGAL |  |  |  |  |  |
| ARSD |  |  |  |  |  |
| TBC1D30 |  |  |  |  |  |
| COL4A1 |  |  |  |  |  |
| TPSAB1 |  |  |  |  |  |
| EFNA5 |  |  |  |  |  |
| SDR16C5 |  |  |  |  |  |
| RWDD2B |  |  |  |  |  |
| MYO1D |  |  |  |  |  |
| IFIT3 |  |  |  |  |  |
| QPRT |  |  |  |  |  |
| IDO1 |  |  |  |  |  |
| ITGA6 |  |  |  |  |  |
| EPYC |  |  |  |  |  |
| EVI2A |  |  |  |  |  |
| MMP12 |  |  |  |  |  |
| SLAMF7 |  |  |  |  |  |
| SOX9 |  |  |  |  |  |
| MAGI3 |  |  |  |  |  |
| GNB4 |  |  |  |  |  |
| ST6GALNAC5 |  |  |  |  |  |
| SEZ6L2 |  |  |  |  |  |
| NID2 |  |  |  |  |  |
| AUNIP |  |  |  |  |  |
| RTP4 |  |  |  |  |  |
| FAM214A |  |  |  |  |  |
| RASSF2 |  |  |  |  |  |
| GUCY1B3 |  |  |  |  |  |
| MB |  |  |  |  |  |
| ENO2 |  |  |  |  |  |
| MYO5B |  |  |  |  |  |
| UNC5B |  |  |  |  |  |
| IGHM |  |  |  |  |  |
| FAM84B |  |  |  |  |  |
| IL32 |  |  |  |  |  |
| PRR15L |  |  |  |  |  |
| ATL2 |  |  |  |  |  |
| NFS1 |  |  |  |  |  |
| DNAJC22 |  |  |  |  |  |
| XAF1 |  |  |  |  |  |
| OGFRL1 |  |  |  |  |  |
| NHSL1 |  |  |  |  |  |
| GZMA |  |  |  |  |  |
| RPS23 |  |  |  |  |  |
| IBSP |  |  |  |  |  |
| HOOK1 |  |  |  |  |  |
| IGLJ3 |  |  |  |  |  |
| MTAP |  |  |  |  |  |
| RNASE6 |  |  |  |  |  |
| ASCL2 |  |  |  |  |  |
| FOXD1 |  |  |  |  |  |
| MCOLN2 |  |  |  |  |  |
| JAK3 |  |  |  |  |  |
| PPP1R3B |  |  |  |  |  |
| ARHGAP30 |  |  |  |  |  |
| CEACAM1 |  |  |  |  |  |
| CDKN2A |  |  |  |  |  |
| CFAP44 |  |  |  |  |  |
| FCMR |  |  |  |  |  |
| ARMT1 |  |  |  |  |  |
| CDK6 |  |  |  |  |  |
| APOBEC3F |  |  |  |  |  |
| ITGB4 |  |  |  |  |  |
| LOC730101 |  |  |  |  |  |
| KIF16B |  |  |  |  |  |
| ACOT4 |  |  |  |  |  |
| PSME4 |  |  |  |  |  |
| CD274 |  |  |  |  |  |
| KLF4 |  |  |  |  |  |
| S100A14 |  |  |  |  |  |
| EPHB3 |  |  |  |  |  |
| GINS4 |  |  |  |  |  |
| RHOBTB3 |  |  |  |  |  |
| IGHV3-23 |  |  |  |  |  |
| MS4A4A |  |  |  |  |  |
| GCNT1 |  |  |  |  |  |
| EN1 |  |  |  |  |  |
| UCHL1 |  |  |  |  |  |
| LOC100509457 |  |  |  |  |  |
| TLR8 |  |  |  |  |  |
| JPH1 |  |  |  |  |  |
| C2 |  |  |  |  |  |
| CYP1B1 |  |  |  |  |  |
| GPX7 |  |  |  |  |  |
| LOXL1 |  |  |  |  |  |
| UBD |  |  |  |  |  |
| PRRX1 |  |  |  |  |  |
| PPP2R2C |  |  |  |  |  |
| FCGR2A |  |  |  |  |  |
| TM4SF1 |  |  |  |  |  |
| EEF1A2 |  |  |  |  |  |
| CD52 |  |  |  |  |  |
| TPSB2 |  |  |  |  |  |
| LIMCH1 |  |  |  |  |  |
| PCGF2 |  |  |  |  |  |
| LRP12 |  |  |  |  |  |
| PCDHA1 |  |  |  |  |  |
| ARG2 |  |  |  |  |  |
| ALDH1L2 |  |  |  |  |  |
| FUT8 |  |  |  |  |  |
| NUTM2B-AS1 |  |  |  |  |  |
| TPSB2 |  |  |  |  |  |
| SIX2 |  |  |  |  |  |
| PSMD3 |  |  |  |  |  |
| CABYR |  |  |  |  |  |
| IL2RG |  |  |  |  |  |
| L2HGDH |  |  |  |  |  |
| LOC100506123 |  |  |  |  |  |
| COL8A1 |  |  |  |  |  |
| CCNO |  |  |  |  |  |
| LOC728613 |  |  |  |  |  |
| FAS |  |  |  |  |  |
| MS4A6A |  |  |  |  |  |
| CDYL2 |  |  |  |  |  |
| SNX10 |  |  |  |  |  |
| BASP1 |  |  |  |  |  |
| TRAF4 |  |  |  |  |  |
| LYPD3 |  |  |  |  |  |
| OLFML2B |  |  |  |  |  |
| ARNTL2 |  |  |  |  |  |
| CCDC71L |  |  |  |  |  |
| LOC102725526 |  |  |  |  |  |
| ECM1 |  |  |  |  |  |
| PAPSS2 |  |  |  |  |  |
| BAMBI |  |  |  |  |  |
| KIAA1211 |  |  |  |  |  |
| TINCR |  |  |  |  |  |
| GUSBP3 |  |  |  |  |  |
| PCSK1N |  |  |  |  |  |
| GAL |  |  |  |  |  |
| KATNBL1 |  |  |  |  |  |
| CDT1 |  |  |  |  |  |
| KRT8 |  |  |  |  |  |
| FAM26F |  |  |  |  |  |
| IGHV4-31 |  |  |  |  |  |
| MNDA |  |  |  |  |  |
| ATP2A3 |  |  |  |  |  |
| GZMB |  |  |  |  |  |
| MROH1 |  |  |  |  |  |
| IFIH1 |  |  |  |  |  |
| S1PR3 |  |  |  |  |  |
| ERBB2 |  |  |  |  |  |
| C11orf80 |  |  |  |  |  |
| CD38 |  |  |  |  |  |
| CFI |  |  |  |  |  |
| HOXC10 |  |  |  |  |  |
| SCUBE3 |  |  |  |  |  |
| CPA3 |  |  |  |  |  |
| GALNT3 |  |  |  |  |  |
| NXT2 |  |  |  |  |  |
| SLC1A1 |  |  |  |  |  |
| PCDHB2 |  |  |  |  |  |
| MGST1 |  |  |  |  |  |
| MSL1 |  |  |  |  |  |
| EPCAM |  |  |  |  |  |
