# Supplementary material for: Multi-omics and pan-cancer analysis revealed common molecular signatures to disclose multitargeted anticancer agents through network pharmacology approach
Source: PLoS One. 2026 Jun 1;21(6):e0350614. doi: 10.1371/journal.pone.0350614 (PMC13225668; doi:10.1371/journal.pone.0350614)
Supplement: S3 Table — (DOCX) [file pone.0350614.s007.docx]

**S3 Table:** Docking scores of all protein–drug complexes.

| **Compound Name** | **Ligand ID** | **CCNB1** | **CDK1** | **AURKA** |
| --- | --- | --- | --- | --- |
| AMG-900 | 24856041 | -9.4 | -9.7 | -10.8 |
| GO-6976 | 3501 | -7.2 | -11.9 | -10.4 |
| ALSTERPAULLONE | 5005498 | -7.7 | -11.1 | -8.9 |
| SORAFENIB | 216239 | -8.3 | -10.4 | -9.1 |
| SP-600125 | 8515 | -8.8 | -9.7 | -8.7 |
| INDIRUBIN-3'-MONOXIME | 3707 | -6.9 | -9.7 | -8.1 |
| AG-24322 | 135413565 | -7.4 | -9.5 | -9.6 |
| PROTUBOXEPIN A | 53355697 | -6.8 | -9.5 | -8.6 |
| LY-2090314 | 10029385 | -9 | -9.4 | -8.8 |
| CHEMBL546797 | 910649 | -8.1 | -9.4 | -8.1 |
| CHEMBL578061 | 948954 | -6.9 | -9.2 | -8.4 |
| PROTOAPIGENONE | 11644907 | -6.6 | -9.2 | -8 |
| ENTRECTINIB | 25141092 | -7.8 | -9.1 | -10 |
| ILORASERTIB | 46207586 | -7.4 | -9.1 | -9.1 |
| RG-1530 | 135398512 | -7 | -9.1 | -8.5 |
| RGB-286638 | 11285002 | -7.9 | -9 | -10.6 |
| JNJ-7706621 | 5330790 | -7.7 | -9 | -8 |
| MK-6592 | 16731225 | -7.6 | -8.9 | -9.7 |
| MLN8054 | 11712649 | -7.7 | -8.9 | -9 |
| GW441756X | 16219401 | -6.9 | -8.9 | -8.6 |
| SERTRALINE HYDROCHLORIDE | 63009 | -6.8 | -8.9 | -8.5 |
| MK-5108 | 24748204 | -7.8 | -8.9 | -8.3 |
| AST-487 | 11409972 | -7.6 | -8.8 | -9 |
| NERVIANO | 46911003 | -7.4 | -8.7 | -9 |
| ALISERTIB | 24771867 | -7.8 | -8.7 | -8.7 |
| GSK-269962A | 16095342 | -8.3 | -8.6 | -9.3 |
| SOTRASTAURIN | 10296883 | -8.9 | -8.6 | -9.2 |
| FENOFIBRATE MICRONIZED | 3339 | -6.6 | -8.6 | -7.6 |
| HESPERADIN | 135421442 | -7.8 | -8.5 | -9.5 |
| KW-2449 | 11427553 | -8 | -8.5 | -9 |
| ENMD-2076 | 16041424 | -7.5 | -8.5 | -8.6 |
| SNS-314 | 24995524 | -7.1 | -8.5 | -8.4 |
| DANUSERTIB | 11442891 | -8.1 | -8.4 | -9.6 |
| NVP-TAE684 | 16038120 | -7.5 | -8.4 | -8.5 |
| CP-547632 | 9811611 | -6.8 | -8.4 | -7.8 |
| BMS-345541 | 9813758 | -5.8 | -8.4 | -7.3 |
| ZOTIRACICLIB | 16739650 | -7.8 | -8.3 | -9.6 |
| XL228 | 59757974 | -7.7 | -8.3 | -8.9 |
| R547 | 6918852 | -7.4 | -8.3 | -8.7 |
| PAZOPANIB | 10113978 | -7.6 | -8.2 | -9.5 |
| MILCICLIB | 16718576 | -8 | -8.2 | -9.3 |
| CENISERTIB | 11569967 | -7.7 | -8.2 | -9.3 |
| TAMATINIB | 11213558 | -7.7 | -8.2 | -8.7 |
| AZD-1080 | 135564570 | -7.3 | -8.2 | -8.4 |
| MKC-1 | 5327686 | -7.6 | -8.2 | -7.8 |
| PF-03814735 | 51346455 | -8.4 | -8.1 | -9.4 |
| PHA-767491 | 11715767 | -5.9 | -8.1 | -7.1 |
| TOZASERTIB | 5494449 | -7.4 | -8 | -9.3 |
| AT-9283 | 135398495 | -7.8 | -7.9 | -9.3 |
| ALVOCIDIB | 5287969 | -7 | -7.9 | -9.1 |
| ZM447439 | 9914412 | -7.9 | -7.9 | -8.9 |
| BARASERTIB-HQPA | 16007391 | -7 | -7.9 | -8.3 |
| LADUVIGLUSIB | 9956119 | -7.8 | -7.9 | -7.7 |
| PF-562271 | 11713159 | -8 | -7.8 | -9.4 |
| CINNARIZINE | 1547484 | -7.5 | -7.8 | -9.1 |
| RIVICICLIB | 24887371 | -7 | -7.8 | -8.9 |
| VIC 1911 | 71696703 | -8.4 | -7.8 | -8.5 |
| ROTENONE | 6758 | -6.7 | -7.8 | -8 |
| RUCAPARIB | 9931954 | -7.4 | -7.7 | -8.7 |
| CYC-116 | 6420138 | -7.3 | -7.7 | -8.3 |
| AZD5438 | 16747683 | -6.5 | -7.7 | -7.8 |
| ANACARDIC ACID | 167551 | -5 | -7.7 | -6.2 |
| SELICICLIB | 160355 | -6.8 | -7.6 | -7.2 |
| BETA-CARBOLINE | 64961 | -7.6 | -7.6 | -6.7 |
| (RS)-ROSCOVITINE | 5097 | -6.7 | -7.5 | -7.3 |
| BAY 61-3606 | 10200390 | -7.4 | -7.4 | -8.4 |
| ARUNCIN B | 53248678 | -5.4 | -7.4 | -5.7 |
| BI-847325 | 135567102 | -7.2 | -7.3 | -8.9 |
| PHA-793887 | 46191454 | -7.2 | -7.3 | -8.7 |
| DINACICLIB | 46926350 | -6.9 | -7.3 | -8.3 |
| EUPATORIN | 97214 | -6.4 | -7.3 | -8 |
| KENPAULLONE | 3820 | -7.1 | -7.2 | -9.4 |
| BAY 11-7082 | 5353431 | -4.7 | -7 | -5.8 |
| AT-7519 | 11338033 | -6.6 | -6.9 | -8.2 |
| RONICICLIB | 71494949 | -7.1 | -6.9 | -7.9 |
| TAMOXIFEN | 2733526 | -6.5 | -6.5 | -7.8 |
| CLOFIBRATE | 2796 | -5 | -6.5 | -6.3 |
| CLOTRIMAZOLE | 2812 | -6.4 | -6.4 | -8.2 |
| LAUROGUADINE | 3038489 | -6 | -6.2 | -7.1 |
| PATULIN | 4696 | -6.4 | -6 | -5.3 |
| DIMETHYLADENINE | 3134 | -6.7 | -5.4 | -5 |
| FLUOROURACIL | 3385 | -5.6 | -4.8 | -4.7 |
| XL228 | 757 | -4 | -3.3 | -3.6 |
