# Supplementary material for: Multi-omics and pan-cancer analysis revealed common molecular signatures to disclose multitargeted anticancer agents through network pharmacology approach
Source: PLoS One. 2026 Jun 1;21(6):e0350614. doi: 10.1371/journal.pone.0350614 (PMC13225668; doi:10.1371/journal.pone.0350614)
Supplement: S4 Table — (DOCX) [file pone.0350614.s008.docx]

**S4 Table:** Hydrogen bonding and hydrophobic interaction profiles of AMG-900 with AURKA, CCNB1, and CDK1 proteins obtained from molecular docking analysis.

| **Name of the compounds** | **Residues** | **Distance (Å)** | **Bond Category** | **Bond Type** |
| --- | --- | --- | --- | --- |
| CDK1-AMG-900) | LEU A: 135 | 5.53 | Hydrophobic | Pi-Sigma |
|  | LEU A: 135 | 5.49 | Hydrophobic | Pi-Sigma |
|  | ASP A: 86 | 4.07 | Hydrogen | Conventional Hydrogen bond |
|  | GLU A: 8 | 4.46 | Hydrogen | Conventional Hydrogen bond |
|  | LYS A: 9 | 6.28 | Hydrogen | Conventional Hydrogen bond |
|  | LYS A: 9 | 6.46 | Hydrogen | Conventional Hydrogen bond |
|  | ILE A: 10 | 5.22 | Hydrophobic | Pi-Sigma |
|  | ILE A: 10 | 5.35 | Hydrophobic | Pi-Sigma |
|  | ILE A: 10 | 5.23 | Hydrophobic | Pi-Sigma |
|  | LEU A: 83 | 5.61 | Hydrogen | Conventional Hydrogen bond |
|  | ALA A: 31 | 6.94 | Hydrophobic | Pi - Alkyl |
|  | VAL A: 18 | 5.44 | Hydrophobic | Pi - Alkyl |
|  | VAL A: 18 | 5.65 | Hydrophobic | Pi - Alkyl |
| AURKA-AMG-900 | VAL A: 147 | 5.2 | Hydrophobic | Pi-Sigma |
|  | VAL A: 147 | 5.65 | Hydrophobic | Pi-Sigma |
|  | ALA A:160 | 7.21 | Hydrophobic | Alkyl |
|  | LYS A: 143 | 3.78 | Hydrogen | Pi-alkyl |
|  | ASP A: 274 | 4.72 | Hydrogen | Conventional Hydrogen bond |
|  | ASP A: 274 | 3.47 | Hydrogen | Conventional Hydrogen bond |
|  | GLU A: 181 | 5.62 | Hydrogen | Conventional Hydrogen bond |
|  | GLU A: 181 | 4.44 | Hydrogen | Conventional Hydrogen bond |
|  | LYS A:162 | 5.66 | Hydrogen | Pi-Cation |
|  | LEU A:263 | 5.24 | Hydrophobic | Pi-Sigma |
|  | LEU A:263 | 5.88 | Hydrophobic | Pi-Sigma |
|  | LEU A:139 | 4.04 | Hydrophobic | Pi-alkyl |
|  | LEU A:139 | 5.24 | Hydrophobic | Pi-alkyl |
|  | LEU A:139 | 5.48 | Hydrophobic | Pi-alkyl |
| CCNB1-AMG-900 | ASP B:267 | 5.38 | Hydrogen | Pi-Anion |
|  | TYR B: 261 | 4.49 | Unfavorable | Conventional Hydrogen bond |
|  | TYR B: 261 | 5.26 | Unfavorable | Conventional Hydrogen bond |
|  | GLU B: 259 | 4.07 | Hydrogen | Conventional Hydrogen bond |
|  | GLU B: 259 | 4.95 | Hydrogen | Conventional Hydrogen bond |
|  | ARG B:216 | 6.44 | Unfavorable | Conventional Hydrogen bond |
|  | ARG B:216 | 4.4 | Unfavorable | Conventional Hydrogen bond |
|  | PRO B: 263 | 5.72 | Hydrophobic | Pi-Alkyl |
|  | PHE B: 215 | 4.37 | Hydrophobic | Pi-Pi T-shaped |
|  | PHE B: 215 | 6.29 | Hydrophobic | Pi-Pi T-shaped |
|  | PHE B: 270 | 4.18 | Hydrophobic | Pi-Pi Stacked |
|  | PHE B: 270 | 4.93 | Hydrophobic | Pi-Pi Stacked |
